# Network pharmacology modeling identifies synergistic Aurora B and ZAK interaction in triple-negative breast cancer

**DOI:** 10.1038/s41540-019-0098-z

**Published:** 2019-07-08

**Authors:** Jing Tang, Prson Gautam, Abhishekh Gupta, Liye He, Sanna Timonen, Yevhen Akimov, Wenyu Wang, Agnieszka Szwajda, Alok Jaiswal, Denes Turei, Bhagwan Yadav, Matti Kankainen, Jani Saarela, Julio Saez-Rodriguez, Krister Wennerberg, Tero Aittokallio

**Affiliations:** 1 Institute for Molecular Medicine Finland (FIMM), University of Helsinki, Helsinki, Finland; 2 Research Program in Systems Oncology, Faculty of Medicine, University of Helsinki, Helsinki, Finland; 3 Department of Mathematics and Statistics, University of Turku, Turku, Finland; 4 Center for Quantitative Medicine, University of Connecticut School of Medicine, Farmington, CT USA; 5 European Molecular Biology Laboratory, European Bioinformatics Institute (EMBL-EBI), Hinxton, UK; 6 Hematology Research Unit Helsinki, Department of Medicine and Clinical Chemistry, University of Helsinki and Helsinki University Central Hospital, Helsinki, Finland; 7 Medical and Clinical Genetics, University of Helsinki and Helsinki University Hospital, Helsinki, Finland; 8 Institute for Computational Biomedicine, Faculty of Medicine, Heidelberg University, Heidelberg, Germany; 9 Biotech Research & Innovation Centre (BRIC), University of Copenhagen, Copenhagen, Denmark

**Keywords:** Cancer, Computational biology and bioinformatics

## Abstract

Cancer cells with heterogeneous mutation landscapes and extensive functional redundancy easily develop resistance to monotherapies by emerging activation of compensating or bypassing pathways. To achieve more effective and sustained clinical responses, synergistic interactions of multiple druggable targets that inhibit redundant cancer survival pathways are often required. Here, we report a systematic polypharmacology strategy to predict, test, and understand the selective drug combinations for MDA-MB-231 triple-negative breast cancer cells. We started by applying our network pharmacology model to predict synergistic drug combinations. Next, by utilizing kinome-wide drug-target profiles and gene expression data, we pinpointed a synergistic target interaction between Aurora B and ZAK kinase inhibition that led to enhanced growth inhibition and cytotoxicity, as validated by combinatorial siRNA, CRISPR/Cas9, and drug combination experiments. The mechanism of such a context-specific target interaction was elucidated using a dynamic simulation of MDA-MB-231 signaling network, suggesting a cross-talk between p53 and p38 pathways. Our results demonstrate the potential of polypharmacological modeling to systematically interrogate target interactions that may lead to clinically actionable and personalized treatment options.

## Network pharmacology modeling reveals synergistic drug combinations

Triple negative breast cancer (TNBC) is a heterogeneous disease that easily develops drug resistance. To achieve more effective clinical responses, synergistic drug combinations that inhibit multiple survival pathways of cancer are urgently needed. However, pinpointing these drug combinations is difficult, as the number of possible combinations grows exponentially. Tang and co-workers from the University of Helsinki, University of Copenhagen, and University of Heidelberg developed a network pharmacology modeling approach to predict synergistic drug combinations and their underlying target interactions. With dynamic simulation of signaling pathways, they identified a synergistic target interaction that involved Aurora B and ZAK that play a critical role in regulating the survival of TNBC cells. These new combinatorial drug targets warrant further exploration of clinical benefits in treating TNBC.

## Introduction

Aberrant activation of protein targets such as kinases plays a fundamental role in cancer progression. Hundreds of chemical compounds that inhibit dysregulated targets have been under investigation in clinical trials.^1^ However, many such targeted compounds have resulted in a limited efficacy as the cancer cells are capable of exploiting complex genetic and epigenetic bypass mechanisms to escape the mono-targeted treatments. A polypharmacology-based paradigm has therefore been proposed for designing multi-targeted therapy to achieve more effective and sustained clinical responses.^2–4^ However, there remains a practical challenge of how to systematically identify synergistic target interactions that are amenable for combinatorial therapies. It has recently been shown that systems-level compound-target interaction networks that capture both on and off-target effects can reveal functional links between cancer vulnerabilities and target gene dependencies, hence supporting the concept of network pharmacology approach to systematically identify novel target interactions that may inhibit synergistically dysregulated cancer survival pathways.^5–7^

Triple-negative breast cancers (TNBC) constitute a heterogeneous group of breast cancers, defined histologically by the lack of expression of the estrogen receptor (ER), progesterone receptor (PR), and the human epidermal growth factor receptor 2 (HER2). TNBC patients tend to respond initially to a conventional chemotherapy, however, the risk of relapse is high, especially if the pathological complete response (pCR) cannot be achieved.^8^ Therefore, the prognosis for TNBC patients remains poor compared to other main subtypes of breast cancer. TNBC patients and cell line models often show heterogeneous responses to targeted drugs.^9,10^ The limited efficacy of single-targeted drugs is most likely due to multiple survival pathways being activated in TNBC. Further, many prognostic markers (e.g., *EGFR*) are not necessarily among drivers of the cancer initiation and progression.^11^ Thus, there is an urgent need to develop personalized approaches that can suggest more selective, multi-targeted therapies for treating TNBC patients.

Cancer cell lines are being widely used as models for comprehensive drug testing and preclinical investigations. Among the TNBC cell models, MDA-MB-231 has been shown to resemble the transcriptional profiles of the claudin-low tumor TNBC subtype, where the stem cell-like features are enriched.^12^ Further, MDA-MB-231 harbors a *TP53* missense mutation, which occurs in over 50% of human cancers, and is one of the key drivers that contribute to early tumorigenesis and tumor progression in TNBC.^13^ The complex cross-talks between p53 loss of function and other oncogenic pathways partly explain why therapeutic strategies to reactivate mutated p53 have shown only little efficacy in vivo.^14^ MDA-MB-231 also harbors *KRAS* and *BRAF* mutations. Therefore, better understanding of the cross-talks among multiple dysregulated pathways in MDA-MB-231 could lead to general principles toward designing of tailored combinatorial treatments for high-grade TNBCs, where increased RAS/RAF/MEK pathway activity is common.^15,16^

We have developed a network pharmacology model called TIMMA (Target Inhibition interaction using Minimization and Maximization Averaging) that utilizes set theory to predict synergistic drug combinations based on monotherapy drug sensitivity data and drug-target interaction data for a given cancer cell type.^17^ The prediction accuracy of TIMMA has been previously validated using multiple cell lines of different cancer types. For instance, we have tested the TIMMA predictions on MCF-7 breast cancer and BxPC-3 pancreatic cell lines, where the model was able to predict essential and synthetic lethal target pairs as validated by double siRNA knockdown experiments.^17^ In a follow-up study, the TIMMA predictions were also experimentally validated in drug combination experiments for a dedifferentiated liposarcoma (DDLS) cell line.^18^ More recently the TIMMA approach was extended to patient-derived cancer samples.^19^ However, since the TIMMA model predicts drug combinations in a data-driven manner, it lacks a systematic exploration of the mechanisms of action for the predicted drug combinations. Without efficient computational and experimental techniques to validate the underlying target interactions, it remains challenging to identify predictive biomarkers for the drug combination responses for personalized medicine applications.

In the present study, we developed and tested a systematic strategy that combines extensive computational and experimental techniques to explore and therefore better understand why specific drug combinations were predicted to be synergistic by TIMMA in MDA-MB-231 cells. The predicted drug and target combinations were experimentally validated using systematic drug combination and pairwise siRNA knockdown assays, respectively. Interestingly, we found complex interactions among three multi-target kinase inhibitors including midostaurin, nilotinib, and motesanib: while midostaurin and nilotinib synergistically inhibited cell growth, the midostaurin–motesanib combination led to antagonistic effect, resulting in a synthetic rescue of the cancer cells. Through a systematic investigation of the kinome-wide drug-target profiles, we identified a synergistic interaction between Aurora B, a key regulator of mitosis, and ZAK, a key regulator of p38 MAPK pathway. We confirmed the Aurora B and ZAK inhibition synergy using combinatorial siRNA, CRISPR/Cas9, and compound screens. Using a dynamic simulation of the MDA-MB-231-specific cancer signaling network, we further identified the context-dependent cross-talks between p53 and p38 pathways upon the inhibition of Aurora B and ZAK. Using patient data, we showed that *ZAK* expression is negatively correlated with the survival of breast cancer patients. In the TNBC patient subset, we further discovered a specific pattern of *AURKB* and *ZAK* upregulation with frequent *TP53* mutation, suggesting a clinical potential of combined Aurora B and ZAK inhibition for certain groups of TNBC patients. Taken together, our results demonstrated the potential of a systematic computational–experimental strategy to identify novel target interactions that may lead to clinically actionable and personalized combinatorial therapies in cancer.

## Results

### The network pharmacology model predictions are in agreement with drug and siRNA combination experiments

**Fig. 1:**
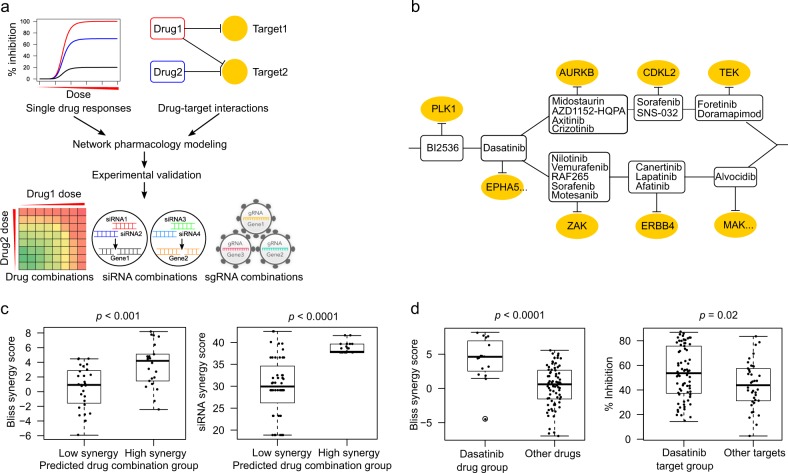
Network pharmacology modeling for MDA-MB-231 cancer cells. **a** Schematic outline of the computational–experimental approach to predicting and validating effective drug combinations and their underlying target interactions. The TIMMA algorithm takes as input single-drug sensitivity profiles and drug-target interaction profiles (here, among 41 kinase inhibitors and 385 kinase targets), and utilizes min–max averaging rules to search a target subset that is most predictive of the observed single-drug sensitivities in the given cells (see Methods). A drug combination is then treated as a combination of the selected targets, the combined effect of which can be quantitatively predicted based on the set relationships between the target profiles of the drugs. The outcome of the TIMMA model consists of a list of predicted drug synergy scores and a drug combination network for further experimental validation. **b** The drug combination network predicted for MDA-MB-231 cancer cells. The network consists of drugs (rectangular nodes) and their kinase targets (oval nodes). An effective drug combination can be inferred by checking whether the removal of them breaks the network into disjoint components (e.g., BI2536–dasatinib combination and dasatinib–midostaurin combination). The EPHA5 and MAK target nodes contain multiple kinases that are unique to dasatinib and alvocidib, respectively, but indistinguishable by their target profiles. **c** The predicted drug combinations and their target interactions were confirmed using pairwise drug combination screen (left) and double knock-down siRNA screen (right) using cell viability assay (CellTiter-Glo). Drug combinations with predicted synergy score higher than the average (0.3485) were classified as high synergy group. **d** The double knock-downs that involved a predicted target of dasatinib showed a stronger cell viability inhibition compared to the other target pairs (right), which may explain the stronger synergies observed in the dasatinib-involving drug combinations compared to non-dasatinib combinations (left). Statistical significance was evaluated using Wilcoxon rank sum test (two-sided)

To prioritize specific drug combinations for a particular cancer cell sample, we made use of our network pharmacology model TIMMA^17^ (Fig. 1a). TIMMA utilized set theory to predict synergistic drug combinations based on monotherapy drug sensitivity profiles and drug-target interaction data for a given cancer cell sample. The algorithm starts by identifying a set of essential drug targets that are most predictive of single-drug sensitivity. A drug combination is then treated as a combination of these essential targets, the effects of which can be estimated based on the set relationships between the drug combination target profiles and the single-drug target profiles. As a case study, here we applied the TIMMA model to single-drug sensitivity profiles of 41 kinase inhibitors in MDA-MB-231 cell line, combined with the kinome-wide drug-target interaction profiles for the 41 compounds covering 385 kinase targets (Supplementary Data 1; Methods). Based on these input data, TIMMA constructed a network pharmacology model comprising of 8 drug-target inhibition nodes among 19 drugs and 20 targets that were most predictive of the drug combination sensitivity in MDA-MB-231 (Fig. 1b). The sensitivity of a drug combination can be inferred from the topology of the network, by checking whether the drug combination inhibits nodes that lead to a breakdown of the network into disconnected subunits. To systematically validate the model predictions on MDA-MB-231, we carried out a drug combination screen involving 50 drug pairs, where several pairs were repeated using different concentration ranges, totaling in 70 combination matrices (Supplementary Data 2). The results showed that the predicted low and high synergy drug combinations were in line with the experimental synergy scores determined by the Bliss model (*p* = 0.0008, Wilcoxon rank sum test; Fig. 1c, left panel; Supplementary Data 2; Supplementary Fig. 1).

We further explored whether the selected targets in the network can explain the observed drug combination synergy. We found that the low and high synergy drug combinations also showed significant differences in the siRNA combination experiments (*n* = 69, *p* < 0.0001, Wilcoxon rank sum test; Fig. 1c, right panel; Supplementary Data 3), suggesting that the model-selected drug targets were able to explain the observed drug synergies. As a specific example, the MDA-MB-231 drug combination network positioned dasatinib and its kinase targets (EPHA5, TXK, BMX, CSK, EPHB1, and EPHB4) as a central hub, which was supported by the enrichment of dasatinib in the top-synergistic drug combinations (*p* < 0.0001, Wilcoxon rank sum test; Fig. 1d, left panel; Supplementary Data 2), as well as by the enrichment of dasatinib targets in the top-effective siRNA combinations (*n* = 110, *p* = 0.017, Wilcoxon rank sum test, Fig. 1d, right panel; Supplementary Data 4). On the other hand, sorafenib uniquely appeared in both of the parallel branches. The removal of dasatinib and sorafenib thus broke the network into three disconnected components, suggesting that such a combination may achieve a maximal sensitivity. In line with such a prediction from the network topology, we experimentally confirmed that the dasatinib–sorafenib combination had the strongest synergy among all the tested drug combinations (Bliss synergy score: 8.2, Supplementary Fig. 2, Supplementary Data 2). On the other hand, BI 2536 was positioned at the root of the network, indicating a strong efficacy for any combination that includes BI 2536 (Supplementary Fig. 3). The model linked BI 2536 sensitivity with the target PLK1, which is known to be critical for the growth of MDA-MB-231 and many other cancer cell lines.^20^ In summary, these results demonstrated that the data-driven drug combination network model can accurately prioritize potent combinations as well as suggest their underlying target interactions for further experimentation.

### Identifying Aurora B–ZAK and Aurora B–CSF1R interactions underlying the predicted drug combinations

**Fig. 2:**
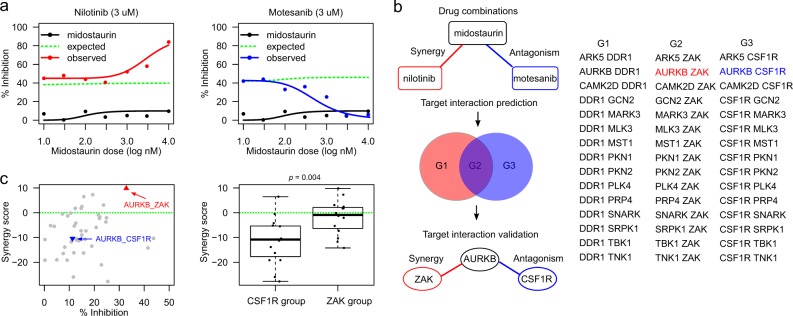
Identification of synergistic and antagonistic target interactions behind drug combinations. **a** Opposite drug combination effects for midostaurin–nilotinib (left panel) versus midostaurin–motesanib (right panel). Motesanib alone produced a minimal effect on cell viability (black curves). In the pairwise combinations, 3 µM nilotinib or motesanib was combined with midostaurin across seven concentrations, ranging from 10 to 10,000 nM. Compared to the reference dose-response curves of no synergy (green dotted lines), nilotinib potentiated midostaurin (red curve in the left panel), while motesanib antagonized midostaurin (blue curve in the right panel). **b** Identification of the target interactions behind the TIMMA-predicted midostaurin–nilotinib synergy and the midostaurin–motesanib antagonism. To explain the synergistic and antagonistic interactions, the possible target combinations were determined from the kinome-wide drug-target interactions^21^ and gene expression data,^22^ resulting in three groups of potential target pairs: Group 1 (G1) contains the target pairs that are unique to midostaurin–nilotinib combination. Group 2 (G2) contains the target pairs that are shared between midostaurin–nilotinib and midostaurin–motesanib combinations. Group 3 (G3) contains the target pairs that are unique to midostaurin–motesanib combination. A kinase was defined as target for a given drug if the dissociation constant (*K*_d_) is lower than 10-fold of the minimal *K*_d_ across all the kinases for this drug. Further, non-expressed targets were removed if their log2 gene expression values were lower than 6 in MDA-MB-231 cells, according to the mRNA expression data from the Cancer Cell Line Encyclopedia.^22^ All the target pairs were profiled in-house using the double siRNA knock-down experiments, resulting in the identification of the synergistic and antagonistic target interactions. **c** Left panel: the percentage inhibition and synergy scores for the target interactions in the siRNA combination experiments. AURKB–ZAK interaction (red triangle) showed top synergy among all the target pairs (*p* < 0.001), while the AURKB–CSF1R interaction (blue triangle) showed strong antagonistic effects (*p* < 0.05). Right panel: the synergy scores for the target interactions involving CSF1R and ZAK separately (*p* < 0.01). The green dotted line shows the baselines of zero synergy. Statistical significance was evaluated using Wilcoxon rank sum test (two-sided)

To focus on potentially more selective combinations (compared to promiscuous dasatinib and sorafenib), we next investigated specifically those drug combinations that inhibit the kinases Aurora B and ZAK, which appeared in the two parallel branches of the network (Fig. 1b). As predicted, we found that midostaurin and nilotinib synergistically decreased cell viability (Bliss synergy score: 4.48, Fig. 2a, left panel). We also found the same level of synergism in the combination of AZD1152-HQPA, a selective Aurora B inhibitor, with two ZAK inhibitors nilotinib (Bliss synergy score: 4.40) and motesanib (Bliss synergy score: 3.41), respectively. These results confirmed the Aurora B–ZAK inhibition synergy in MDA-MB-231 cells. In contrast, the midostaurin–motesanib combination, despite of inhibiting also Aurora B and ZAK, showed a strong antagonistic effect (Fig. 2a, right panel). Such an opposite interaction pattern that involved a common drug (midostaurin) combined with different inhibitors (nilotinib versus motesanib) suggests a polypharmacological complexity due to promiscuous and reversed target interactions. We therefore hypothesized that there may exist antagonistic target interactions that are underlying the midostaurin–motesanib combination (Fig. 2b, top panel).

To probe the target interaction space behind these drug combinations, we extracted the drug-target interaction profiles from a kinome-wide binding affinity assay,^21^ and defined a kinase as a target for a given drug if the dissociation constant (*K*_d_) is lower than 10-fold of the minimal (*K*_d_) across all the kinases for the particular drug. The drug-specific thresholds were used due to the very different inherent (on-target and off-target) activities between kinase inhibitors.^7^ Furthermore, we focused on the expressed targets by removing non-expressed targets, i.e., log2 gene expression values lower than 6, according to the transcriptomic profiles of MDA-MB-231 from Cancer Cell Line Encyclopedia^22^ (Fig. 2b; Supplementary Data 5). This filtering process led to the identification of 45 target pairs involving 18 genes that can be classified into three groups, depending on whether they are shared by the midostaurin–motesanib and midostaurin–nilotinib combinations (Fig. 2b, middle panel). We then carried out a siRNA combination screen testing systematically all the 45 target pairs. The results again confirmed a strong synergy between Aurora B and ZAK (Fig. 2c, left panel; Supplementary Data 6–7). On the other hand, we found that a majority of the antagonistic target interactions involved CSF1R, including the Aurora B–CSF1R pair (Fig. 2c, right panel). Although the Aurora B–CSF1R interaction was not the most antagonistic pair, the interplay among Aurora B, ZAK, and CSF1R in the midostaurin–motesanib combination suggested intriguing dual roles of Aurora B towards ZAK and CSF1R that lead to unexpected opposite interactions.

**Fig. 3:**
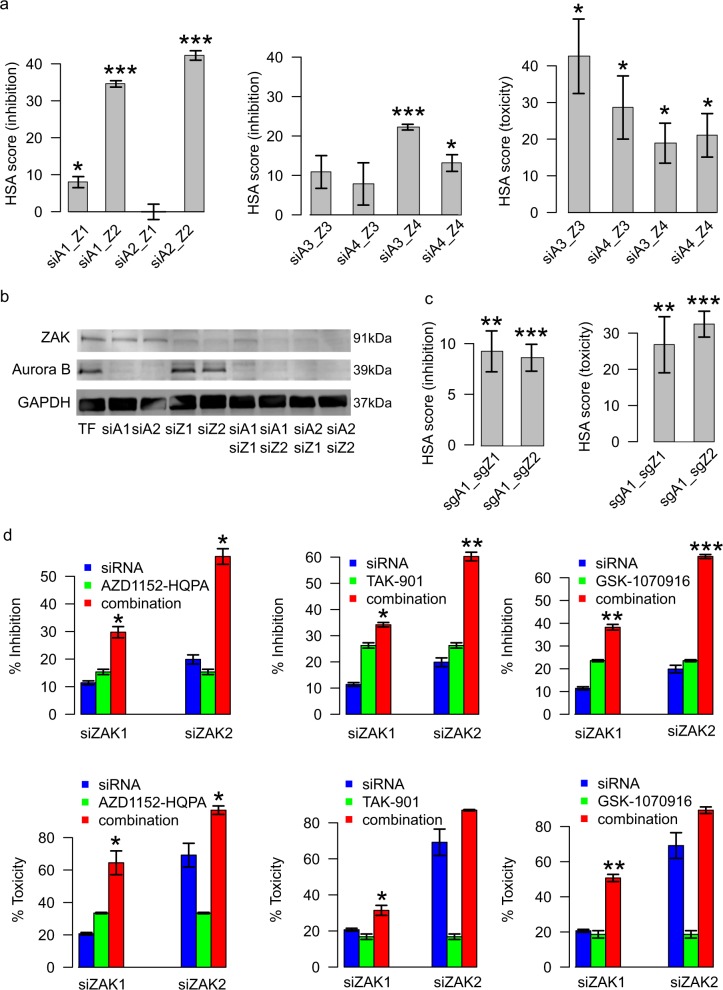
Experimental confirmation of the Aurora B and ZAK interactions in MDA-MB-231. **a** Validation of the AURKB–ZAK interactions using two Qiagen siRNAs (siA1 and siA2) and two Ambion siRNAs (siA3 and siA4) for *AURKB*, and similarly for *ZAK* (siZ1–siZ4). For each siRNA, 16 nM of final concentrations were used for both single siRNAs and double siRNAs (i.e., an 8+8 nM combination in double siRNAs). The highest single agent (HSA) synergy scores were calculated as the difference between the siRNA double knockdown effects minus the maximal effects of the single knockdowns in cell viability inhibition (CellTiter-Glo) and toxicity (CellTox Green) assays, respectively (see Methods for details). Standard error of means was calculated over three replicates. **b** Knockdown effect of *AURKB* and *ZAK* by each of the individual Qiagen siRNAs and their combinations using Western blot assays. Standard error of means was calculated over three replicates. **c** HSA synergy scores for *AURKB* and *ZAK* double knock-out using combinatorial sgRNAs (sgA1 for *AURKB* and sgZ1, sgZ2 for *ZAK*) in CRISPR/Cas9 system. Standard error of means was calculated over eight replicates. **d** Cell inhibition and toxicity effects were measured for Aurora B inhibitors combined with the two *ZAK* siRNAs. *p < 0.05; **p < 0.01; ***p < 0.001 (Wilcoxon rank sum test, two-sided). The labels on the *x*-axis indicate the different siRNA combinations

To further confirm the synergistic interaction between Aurora B and ZAK in MDA-MB-231 cells, we performed a non-pooled siRNA knock-down combination screen, using four independent siRNAs from two providers (Qiagen and Ambion) (Supplementary Data 8). Although there were differences between the individual siRNA efficacies, the majority of double knock-downs showed significant synergy in both cell viability (CellTiter-Glo) and cell toxicity (CellTox Green) assays (Fig. 3a; Supplementary Fig. 4). Further, the knock-down efficiency of the Qiagen siRNAs for *AURKB* and *ZAK* was confirmed both at the transcript and protein levels (Fig. 3b, Supplementary Data 9). To confirm whether such synergies are also observed with loss-of-function gene knock-outs, we performed combinatorial CRISPR/Cas9 cell viability and toxicity screens, and found a similar level of synergistic effects in three out of the four individual *AURKB* and *ZAK* double sgRNAs (Fig. 3c; Supplementary Fig. 5). Due to the lack of highly selective ZAK inhibitors, we further evaluated *ZAK* siRNAs in combination with three selective Aurora B kinase inhibitors (AZD1152-HQPA, TAK-901, and GSK-1070916). Although the siRNA-specific differences were again present, significantly higher inhibition measured by CellTiter-Glo and cytotoxicity effects by CellTox Green were observed in all the combination experiments, compared to the use of inhibitors alone (Fig. 3d).

To confirm that the predicted synergistic interaction between Aurora B and ZAK is cell context-specific, we applied the TIMMA prediction model also to MDA-MB-361, an ER-positive, HER2-positive, and PR-negative breast cancer cell line, as well as to another triple negative cell line, MDA-MB-436 (Supplementary Data 10–11). In line with the model predictions, the AURKB inhibitors combined with *ZAK* siRNAs showed synergy in MDA-MB-436 (predicted synergy score = 0.28), while the interaction became antagonistic in MDA-MB-361 (predicted synergy score = 0.17) (Supplementary Fig. 6), suggesting a context-specificity of the identified target interactions.

### Understanding Aurora B–ZAK and Aurora B–CSF1R interactions by dynamic simulation of signaling pathways

**Fig. 4:**
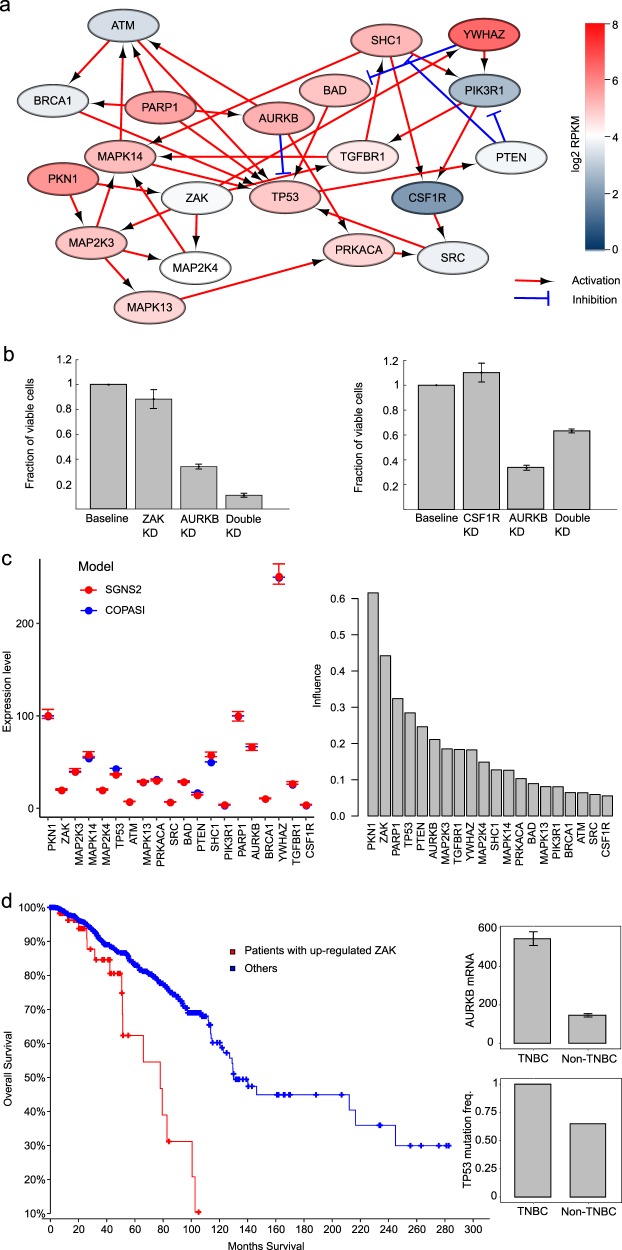
Dynamic modeling of MDA-MB-231 signaling network supports the context-specific combination effects. **a** Signaling network based on selected interaction partners of Aurora B, ZAK, and CSF1R. The node colors indicate the log_2_ mRNA expression levels of the genes. Arrow-heads represent activation and bar-headed edges represent inhibition of the target proteins, retrieved from OmniPath.^23^ Red-circled area highlights the p38 pathway that may be activated by ZAK and blue-circled area suggested the role of TGF-β pathway that involves CSF1R. **b** Simulated cell viability in response to single and double gene knock-downs. The fraction of viable cells decreased further when both AURKB and ZAK were silenced, while a simultaneous knock-down of AURKB and CSF1R increased the cell proliferation compared to the knock-down of AURKB alone. Standard error of means was calculated over ten replicates. **c** Left panel: the steady state expression levels of genes inferred by SGNS2 and COPASI. Right panel: the average influence of the *K*_d_ (degradation rate) and *K*_p_ (production rate) parameters of each gene on the expression level of all the genes in the signaling network. **d** Left panel: the overall survival curves for breast cancer patients with higher ZAK gene expression (*z*-score > 1.5 in RNA-Seq data, *n* = 59) versus the others (*n* = 1036). Right panel: the *AURKB* gene expression and *TP53* mutation frequency differences between TNBC and non-TNBC patients. Error bars represent standard errors

To investigate the potential mechanisms of how ZAK and CSF1R partners mediate their dual roles with Aurora B in the synergistic and antagonistic interactions, we compiled a signaling network consisting of proteins that have previously been reported to interact with either Aurora B, ZAK, or CSF1R, for which the directions and signs of the network connections were retrieved from OmniPath,^23^ a comprehensive collection of human signaling pathways curated from the literature (Supplementary Data 12; Methods). To make the signaling network specific to MDA-MB-231, genome-wide gene expression profiles from a recent RNA-seq study^24^ were utilized to further pinpoint 20 proteins that were differentially expressed in MDA-MB-231 cell compared to other cancer cell lines (Supplementary Fig. 7, Supplementary Data 13). The constructed signaling network positioned wildtype p53 as the central hub, which interacts with Aurora B directly, while the links with ZAK and CSF1R were established via the p38 MAPK pathway and TGF-β pathway, respectively (Fig. 4a). While these links cannot be claimed to be causal, we speculated that the observed synergistic and antagonistic interactions may be related to the cross-talk between p53 and p38 pathways. To model the signaling network, we further determined the degradation and production rates for each gene based on their Reads Per Kilobase of transcript, per Million mapped reads (RPKM) values.^24^ To accurately capture the effect of inherent stochasticity in gene expression, we simulated the model using SGNS2 (Stochastic Genetic Network Simulator).^25,26^ We chose SGNS2 because of its computational efficiency and ability to model partitioning at division.^27^ In our model, cell growth was assumed to be TP53 dependent. More specifically, in the absence or low expression of TP53, MDA-MB-231 cells grew exponentially and divided according to their experimentally observed doubling time. With an increased TP53 expression, its effect on cell growth was modeled using a Hill function. The simulation results suggested that the knock-out of AURKB and ZAK leads to loss of cell viability, while the knock-out of AURKB and CSF1R leads to gains in cell viability (Fig. 4b).

To investigate the robustness of the simulation results and the sensitivity of the model parameters, we next transformed the stochastic model into an ordinary differential equation (ODE)-based model in COPASI,^28^ which is a widely used open-source simulator for biochemical reaction networks. We chose COPASI as it provides optimized functionality for sensitivity analysis,^28,29^ which is currently lacking in SGNS2. To verify the proper transformation of the model from stochastic to ODE-based, we compared the steady state expression levels of genes in both of the models. The model comparison results showed that the steady state expression levels of genes inferred by the ODE model were similar to those obtained using the stochastic simulation model, suggesting the validity of the signaling network simulation results (Fig. 4c, left panel). Furthermore, we also found that the degradation and production rate parameters for ZAK were among the top ones that significantly affected the expressions of other genes (Fig. 4c, right panel, Supplementary Data 14), suggesting the importance of ZAK, for which more experimental validation is needed. The other sensitive genes included TP53, which is closely related to AURKB, and those genes that have no input connections (e.g., PKN1 and PARP1). On the other hand, CSF1R showed a minimal influence on the gene expressions of the whole network, thus suggesting that its inhibition should not affect the survival of the MDA-MB-231 cancer cells. This further supports the observed antagonistic effect of CSF1R and AURKB, where the inhibition of both rescued the cancer cells.

We further analyzed the gene expression data of all the breast cancer cell lines available from the Klijn et al. study.^24^ The AURKB and ZAK gene expression levels were significantly higher in TNBC than in non-TNBC cell lines (two-sided Wilcoxon rank sum test, *p* < 10^−5^, Supplementary Fig. 8, Supplementary Data 15), suggesting that AURKB and ZAK targets are up-regulated in TNBC. Finally, we retrieved the TCGA patient tumor data from the cBioPortal platform to query the clinical frequency and relevance of the identified *AURKB*, *ZAK*, and *TP53* status in breast cancer patients.^30^ Interestingly, we found that a higher expression of *ZAK* is associated with poor survival of breast cancer patients (log-rank test, *p* < 0.0001, *n* = 1105, Fig. 4d). This observation was corroborated by a recent study of the key role of *ZAK* in promoting the epithelial–mesenchymal transition in cancer progression, which showed that *ZAK* overexpression is significantly associated with poor survival in a number of human cancer types including breast cancer.^31^ In the TNBC subset of breast cancer patients (*n* = 117), the *TP53* mutation frequency and the *AURKB* gene expression were found to be significantly higher compared to non-TNBC patients (Wilcoxon rank sum two-sided test, *p* < 0.0001). Furthermore, we found that the median survival was lower in the subgroup of TNBC patients with *AURKB* *z*-score > 2, *ZAK* *z*-score > 0, and *TP53* mutation (51.1 months versus 114.1 months, *p* = 0.04, Fleming–Harrington test with weights *p* = 1 and *q* = 1), but not in the subgroup of TNBC with *AURKB* *z*-score > 2, *CSF1R* *z*-score < 0, and *TP53* mutation (Supplementary Fig. 9). The up-regulation of MAP2K3 and MAPK11, the key regulators of p38 pathway, was also associated with lower survival rates (Supplementary Fig. 10). The aberrant status of *TP53*, *AURKB*, and *ZAK* might therefore suggest clinical, selective benefits of the combinatorial *AURKB* and *ZAK* inhibition to perturb the p53 and p38 pathways in TNBC patients.

## Discussion

Given the vast search space of possible drug combinations, combined with the heterogeneity of survival dependencies in many cancer types, efficient prioritization of the most potential drug combinations for a given cancer type or patient subgroup pose a clinical and experimental challenge. By integrating drug-target interaction profiles and single-drug sensitivities, we have previously developed a logic-based network pharmacology modeling approach, called TIMMA, for systematic prioritization of effective drug combinations for a given cancer sample.^17–19^ The TIMMA model generates a data-driven hypothesis for selecting potential drug combinations, while downstream analyses are required to leverage the polypharmacologic target information as well as molecular profiling data to pinpoint the actual target interactions. The novel contribution of the present study was therefore to apply a series of computational and experimental methods to systematically explore the predicted drug combinations and further validate their target interactions. Experimental validation techniques included combinatorial siRNA, CRISPR/Cas9, and drug synergy screening experiments, combined with computational mining of the kinome-wide drug-target interaction data and gene expression profiles to pinpoint the underlying target interactions.

In the last step, dynamic modeling of signaling pathways using stochastic simulation algorithm (SSA) was implemented to understand the mechanisms of action of the identified target interactions. Using the dynamic modeling approach, we constructed a MDA-MB-231 signaling network to simulate the effect of perturbing the genes of interests on the cell viability. The qualitative similarity of the model predictions to the experimental observations suggests that the constructed signaling network comprises of key protein–protein interactions that may mediate the synergistic and antagonistic relationship of Aurora B with ZAK and CSF1R, respectively. While we chose to use dynamic signaling model, given the proper use of prior based on available data and interactions, we believe that a causal Bayesian network modeling or any other mechanistic modeling approach would produce similar insights. This proof-of-concept study introduced a systematic experimental–computational pipeline to identify combinatorial vulnerabilities of MDA-MB-231 cells, but it can be also applied to other cell lines and patient-derived samples.

Among the identified drug combinations, we specifically explored the underlying target interactions that resulted in opposite cell growth phenotypes: the inhibition of Aurora B and ZAK showed a synergistic effect in MDA-MB-231, while inhibiting CSF1R rescued the cells from the Aurora B inhibition. Dynamic simulations of the signaling pathways indicated that Aurora B, ZAK, and CSF1R might be involved in the cell division processes related to p53, while the cross-talks with p38 MAPK or TGF-β pathways seem to lead to different states in the cell cycle. Since direct targeting of mutant p53 has not yet been successful,^32^ the Aurora B and ZAK inhibition may be considered as an indirect strategy to effectively restore the normal function of p53. On the contrary, the Aurora B and CSF1R inhibition showed antagonistic interaction, which is supported by the study^33^ of Patsialou et al., 2014, in which the CSF1R signaling was shown to mediate a switch between the invasion and proliferation states via the TGF-β pathway in MDA-MB-231, and thus may provide new insights into the tumor progression.

Synergistic drug combinations are rare in the clinic. Most drug combinations have been approved as they showed higher clinical efficacy than monotherapies. However, due to the large variability in the monotherapy responses, it is not surprising that a drug combination where two drugs act independently is sufficient to achieve a higher clinical efficacy in a patient population.^34^ However, to achieve a more effective cancer treatment, we aimed here to identify personalized drug combinations that work synergistically. TNBC is known to be a collection of very heterogeneous diseases, both in terms of genetic background and therapy responses, making it a highly challenging case for combinatorial therapy prediction. Instead of relying on established molecular subtypes of TNBC, we made use of the comprehensive drug sensitivity profiling, which is likely to provide more actionable starting point for precision medicine of TNBC.^10^ We have previously shown that the TNBC cell lines present with highly heterogeneous responses to anticancer drugs.^10^ Therefore, when applied to patient-derived samples, we argue that personalized combinatorial treatment options need to be tailored based on individual ex vivo drug sensitivity profiles for each primary patient sample separately, and ideally compared to healthy control profiles, whenever available. As was recently demonstrated in a case study on leukemia patients,^19^ polypharmacological modeling approaches such as the one proposed here has several benefits for clinical translation: (i) it identifies patient-selective target combinations, rather than broadly toxic effects that often lead to severe side-effects, (ii) compared to genomics-only approach, the predictions on the target interactions are pharmaceutically actionable, (iii) it provides unbiased, experimentally testable predictions for treatment decision making when the patient-derived and comparable control cells are available.

We used combinatorial drug screening, complemented with RNAi and CRISPR/Cas9 knockout experiments for systematic and detailed validation of the model predictions, due to the known limitations and complementary nature of these assays.^35^ Despite the experimental variation that was observed in siRNA and CRISPR/Cas9 systems, we confirmed that the Aurora B and ZAK interaction could explain the synergy between midostaurin and nilotinib. The Aurora B and CSF1R interaction also explained the antagonism between midostaurin and motesanib, consistent with its role in breast cancer development.^36^ The successful model predictions for experimentally-validated drug combinations and their target interactions exemplified the rationale of leveraging polypharmacology data and network modeling to identify cell-specific drug combinations. The dynamic network simulations also provided clues about the potential mechanisms behind the observed synergistic effects between AURKB and ZAK interaction in this particular cell-context. Even though the parameter sensitivity analysis showed that our signaling network model is fairly robust (with a small set of sensitive parameters such as the degradation and production rates of ZAK), in future, a more systematic analysis of parameter sensitivity will be required to generate data-driven hypothesis about the mechanisms of action behind other interactions in different cancer contexts. To validate the data-driven hypothesis about the p38 and p53 pathway interaction, a more comprehensive analysis in other TNBC breast cancer cell lines is needed.

As a future development, integration of the drug combination networks with known signaling interactions between the selected drug targets and cancer-driving mutations should be explored in a combined manner to make even more accurate drug combination and target interaction predictions for individualized anticancer treatments. Although we focused here on MDA-MB-231 as our primary model system, the data-driven network pharmacology approach is widely applicable to a rational design and understanding of unexpected drug combinations also in other cancer cell lines or patient-derived samples, once systematic functional assay data together with molecular and genomic profiles become increasingly available.

## Methods

### Cell lines

Human breast cancer cell lines MDA-MB-231, MDA-MB-361, and MDA-MB-436 were obtained from ATCC and were maintained in 10% FBS-DMEM (Life Technologies) at 37 °C with 5% CO_2_ in a humidified incubator, according to provider’s instructions.

### Kinase inhibitors collection and the TIMMA network pharmacology modeling

Utilizing drug-target interaction data and single-drug sensitivities, the TIMMA algorithm started by identifying a set of essential targets that are most predictive of single-drug sensitivity (Supplementary Data 16). The mathematical details of the TIMMA modeling can be found in Tang et al.^17^ Briefly, for a drug combination with a target profile *d*, the drug combination sensitivity *y*_*d*_ can be predicted based on the set relationships between *d* and the target profiles for the *N* single drugs *d*_*i*_, *i* ∈ *N*:

1 $$\left\{ {\begin{array}{*{20}{c}} {y_d = \frac{{\mathop {\sum}\limits_{i \in N} {I(d\, =\, d_i)y_i} }}{{\mathop {\sum}\limits_{i \in N} {I(d\, =\, d_i)} }},\;{\mathrm{if}}\;\mathop {\sum}\limits_{i \in N} {I(d = d_i)} \ne 0} \\ {y_d = (y_{\min } + y_{\max })/2,{\mathrm{if}}\;\mathop {\sum}\limits_{i \in N} {I(d = d_i)} = 0} \end{array}} \right.$$

where $$I(d = d_i) = \left\{ {\begin{array}{*{20}{c}} {1,{\mathrm{if}}\;d = d_i} \\ {0,{\mathrm{if}}\;d \ne d_i{\mathrm{ }}} \end{array}} \right.,$$

$$y_{\min } = \frac{{y_h + \mathop {\sum}\limits_{j \in N,j \ne h} {I(d \supset d_j \cap } d_j \supset d_h \cap y_j < y_h)y_j}}{{1 + \mathop {\sum}\limits_{j \in N,j \ne h} {I(d_{new} \supset d_j \cap } d_j \supset d_h \cap y_j < y_h)}},h = \mathop {{\arg \max }}\limits_{i \in N} (I(d \supset d_i)y_i)$$

$$y_{\max } = \frac{{y_l + \mathop {\sum}\limits_{j \in N,j \ne l} {I(d_{new} \subset d_j \cap } d_j \subset d_l \cap y_j > y_l)y_j}}{{1 + \mathop {\sum}\limits_{j \in N,j \ne l} {I(d_{new} \subset d_j \cap } d_j \subset d_l \cap y_j > y_l)}},\mathop {{l = \arg \min }}\limits_{j \in N} (I(d \subset d_j)y_j)$$

The single drug sensitivity data *y*_*i*_, *i* ∈ *N* for the MDA-MB-231 cell line were quantified as drug sensitivity score (DSS)^37^ derived from cell viability readout, which was further normalized into the [0, 1] interval where a higher value indicates a more sensitive drug. The drug-target interaction data for the 41 kinase inhibitors were retrieved originally from the KINOMEscan binding affinity assay platform, covering a panel of 385 kinases covering the majority of catalytically active human protein kinases.^21^ For each drug, targets that showed 50-fold or less of the minimal binding affinity *K*_d_ levels were considered as the positive targets (including both on and off-targets) for the TIMMA modeling (Supplementary Data 1). These targets were further narrowed down by applying a more stringent 10-fold threshold to identify the target interactions for the midostaurin and nilotinib synergy as well as for the midostaurin and motesanib antagonism (Supplementary Data 5).

### Validation of the drug combination network for MDA-MB-231 using drug combination and siRNA combination screens

For the primary screening of kinase targets predicted by the drug combination network (Fig. 1b), three different siRNAs (Qiagen) for each gene were pooled together at a total concentration of 6 nM. A total of 18 kinase targets were evaluated both individually and in pairwise combination in the siRNA screens, for which the percentage inhibition data were retrieved from Dataset S8 in Tang et al.^17^ For each drug pair (*d*_1_, *d*_2_), the siRNA-based synergy score was calculated by averaging the multiplicative synergy scores of the corresponding target pairs:

2 $$S_{{\mathrm{siRNA}}} = \frac{1}{n}\mathop {\sum}\nolimits_{i{\it{\epsilon }}d_1,j{\it{\epsilon }}d_2} {\left( {y_{i,j} - y_iy_j} \right),}$$

where *i*,*j* are the targets of drugs *d*_1_,*d*_2_, respectively and *y*_*i.j*_, *y*_*i*_ and *y*_j_ are the % inhibition levels of the double knock-down and single knock-down; *n* is the total number of target pairs underlying the drug pair.

To further validate the drug combination predictions, 50 drug pairs (of which 16 drug pairs were tested in duplicates and 2 drug pairs in triplicates with different concentration ranges, see Supplementary Data 2 and Supplementary Fig. 1) were screened in an 8 × 8 dose-response matrix assay with positive and negative controls. We did not test all the drugs in the network in pairwise combinations. For example, BI 2536 alone already inhibited the cancer cell survival, and therefore was expected to result in a limited synergy in combinations with other drugs. For the rest of the nodes in the network, we rather focused on more critical targets and interactions including the hub nodes and node combinations that break down the connectivity structure of the graph.

The cell growth phenotype was measured as percentage inhibition using a luminescent cell viability assay (CellTiter-Glo, Promega), following the protocols that were described previously.^10^ Scoring of drug combinations was done by first fitting the dose-response curves using the four-parameter logistic models using the drc package in R.^38^ The volumes between the observed response surface and the expected response surface using the Bliss model were used to quantify the average synergistic effects of a drug combination based on the 7 × 7 dose combinations of the two drugs:

3 $$S_{{\mathrm{Bliss}}} = \frac{1}{{7 \times 7}}\mathop {\sum}\limits_{i,j}^7 {\left( {y_{{\mathrm{combination}},i,j} - y_iy_j} \right)} ,$$

where *y*_combination_ is the percentage inhibition value of drug 1 and drug 2 combined at concentration *i* and *j*, while *y*_*i*_ and *y*_*j*_ are the percentage inhibition values of the single drugs at *i* and *j*, respectively. The Bliss synergy scores were also visualized in a three-dimensional landscape on the dose-response matrix (Supplementary Fig. 2), similar to the method that was used in Yadav et al.^39^

### Validation of AURKB and ZAK interaction using RNAi combination screens

For the confirmation screening of target interactions for midostaurin–nilotinib and midostaurin–motesanib, we utilized the kinome-wide drug-target profiles^21^ to derive the common target pairs for these two drug combinations, as well as those that are exclusively present for only single drug treatment. We then utilized the gene expression data to filter out those inactivated targets in MDA-MB-231 cells. 45 target pairs remained which can be classified into three categories in the Venn diagram shown in Fig. 2b. Three siRNAs (Qiagen) for each target gene were selected and tested individually in the combinations against MDA-MB-231 in 384-well plate format with 500 cells per well. 16 nM (total concentration) of siRNAs were used in both single and double RNAi mediated knock-downs, with three replicates. The percentage inhibition values for each gene pair were calculated as the average over all the 3 × 3 siRNA combinations.

For the confirmation screening of AURKB–ZAK interaction, three siRNAs for each target gene were purchased from Qiagen and Ambion, respectively. Two Qiagen siRNAs and two Ambion siRNAs that showed more consistent phenotypes to each other were selected in the subsequent data analyses. For the Ambion siRNAs, CellTox Green assays (Promega) were further implemented to access the cytotoxicity of the siRNA knock-downs, following the protocols that were described previously.^10^

Since the same concentrations were used in single and double siRNA knock-downs, the highest single agency (HSA) synergy score was used to assess the degree of synergy:

4 $$S_{{\mathrm{HSA}}} = y_{{\mathrm{combination}}} - {\mathrm{max}}(y_1,y_2).$$

The statistical significance was based on testing whether the synergy score is different from zero based on Wilcoxon rank sum test (two-sided).

### RT-qPCR and western blot analysis

siRNA transfected cells were lysed using RealTime ready Cell Lysis Kit (Roche) and cDNA was synthesized using Transcriptor Universal cDNA Master kit (Roche). PCR reactions with LightCycler 480 SYBR Green I Master mix (Roche) were detected with LightCycler 480 II Instrument (Roche). The primer sequences for *AURKB*, *ZAK* and the housekeeping gene *HMBS* are shown in Supplementary Data 9.

To obtain sufficient amount of material for the experiments, 96-well plates with 6000 cells/well were used and siRNA transfections were performed at 64 nM due to the increase of cell concentrations. The percentage expression of the target gene after siRNA mediated knock-down was calculated as:

5 $${\mathrm{\% }}\;{\mathrm{gene}}\;{\mathrm{expression}} = 100 \times 0.5^{({\mathrm{Cp}}_1 - {\mathrm{Cp}}_2)}/0.5^{({\mathrm{Cp}}_3 - {\mathrm{Cp}}_4)}$$

where Cp_1_ and Cp_2_ are the cross-point numbers of the gene after and before knock-down; Cp_3_ and Cp_4_ are the cross-point numbers of the housekeeping gene.

For western blot, siRNA transfected cells were lysed in 4% sodium dodecyl sulfate (SDS), 0.1 M Tris–HCl pH 7.5, 0.1 M dithiothreitol (DTT). Protein lysates were run on a 12% polyacrylamide gel with SDS and transferred to a polyvinylidene difluoride (PVDF) membrane that was blocked with 5% bovine serum albumin (BSA) in Tris-buffered saline (TBS). After blocking the membrane, it was incubated with primary antibodies anti-ZAK (Bethyl Laboratories, A301-993A, mouse monoclonal antibody), anti-Aurora B (Novus Biologicals, NB110-55480, rabbit monoclonal antibody), GAPDH (Sigma-Aldrich, G8795, mouse monoclonal antibody) in 5% BSA in Tris-buffered saline and 0.05% Tween 20 (TBS-T). The membrane was incubated with secondary antibodies anti-mouse IRDye 680 and anti-rabbit IRDye 800CW (Odyssey; LI-COR Biosciences) in 5% BSA TBS-T. Odyssey Imaging System (LI-COR Biosciences) was used to visualize the proteins. All blots derived from the same experiment and were processed in parallel.

### sgRNA combination screens

Two oligonucleotides encoding sgRNAs against *AURKB* and *ZAK* were ordered from SigmaAldrich (Supplementary Data 9). The sgRNAs were then crossed in the combinations. Oligonucleotides were cloned into LentiGuide plasmid (Addgene Plasmid #52963) as previously described.^40^ For packaging, LentiGuide or LentiCas9 (Addgene Plasmid #52962) lentiviral plasmids and packaging plasmids pCMV-VSV-G (Addgene Plasmid #8454) and pCMV-dR8.2 dvpr (Addgene Plasmid #8455) were transfected into 293T cells. Transfection was done using Lipofectamine 2000 (Thermo Fisher Scientific) reagent according to the manufacturer’s instruction. Virus supernatants were collected 24 h post transfection.

Cells were seeded in 24-well plate format at a density of 5 × 10^4^ cells/cm^2^. Two hours after seeding culture media was replaced with media containing lentiviral particles (lentiCas9, MOI = 5) and polybrene (8 μg/ml). Next day, media was supplemented with Blasticidine (6 μg/ml) and cells were selected for 7 days. Cas9 expressing cell lines were seeded in a 24-well plate format (5 × 10^4^ cells/cm^2^) and incubated with lentivirus particles expressing sgRNA (MOI = 10 for each virus) and polybrene (8 μg/ml). Next day, the media was replaced with MDA-MB-231 growth media containing 1 μg/ml puromycin. Cells were allowed to grow for 3 days. After that cells were seeded in 96-well format (1000 cells per well) and grown for 5 days. Cell viability and cell death were measured with CellTiter-Glo (CTG) Luminescent Cell Viability Assay and CellTox Green Cytotoxicity Assay (Promega Inc.) correspondingly. The cell viability was calculated as the ratios of CellTiter-Glo readouts between day 5 and day 1 after transfection:

6 $${\mathrm{Cell}}\;{\mathrm{viability}} = \frac{{{\mathrm{CellTiter}} - {\mathrm{Glo}}^{{\mathrm{day}}5}}}{{{\mathrm{CellTiter}} - {\mathrm{Glo}}^{{\mathrm{day}}1}}}$$

The percentage of inhibition was derived by normalizing the viability to the positive and negative control sgRNAs.

The cytotoxicity values were calculated as the ratio of cell death to cell viability, hence more cell death and less viable cells indicate stronger cytotoxicity. The cell death was measured as the CellTox Green readout at day 5; the cell viability was calculated as the CellTiter-Glo readout at day 5 divided by the initial readouts at day 1:

7 $${\mathrm{Cytotoxicity}} = \frac{{{\mathrm{Cell}}\;{\mathrm{death}}}}{{{\mathrm{Cell}}\;{\mathrm{viability}}}} = \frac{{{\mathrm{CellTox}} - {\mathrm{Green}}^{{\mathrm{day}}5}}}{{{\mathrm{CellTiter}} - {\mathrm{Glo}}^{{\mathrm{day}}5}/{\mathrm{CellTiter}} - {\mathrm{Glo}}^{{\mathrm{day}}1}}}$$

### RNAi + drug combination screens

MDA-MB-231, MDA-MB-361, and MDA-MB-436 were screened against 1000 nM of AZD1152-HQPA and GSK-1070916, 100 nM of TAK-901, in combination with 16 nM of *ZAK* siRNA. TAK-901 and GSK-1070916 were purchased from Selleck (S2718, S2740) while AZD1152-HQPA was purchased from ChemieTek (CT-A1152H).

Percentage inhibition values in the CellTiter-Glo assay and percentage cytotoxicity values in the CellTox Green assay were calculated by using DMSO as negative control and 100 µM benzethonium chloride (BzCl) as positive control. The HSA synergy score was calculated.

### Construction of the MDA-MB-231 signaling network model

Differentially-expressed genes in MDA-MB-231 were determined by calculating the distance:

8 $$D = \frac{{{\mathrm{abs}}\left( {{\mathrm{Expression}}_{{\mathrm{MDA}} - {\mathrm{MB}} - 231} - {\mathrm{Mean}}\left( {{\mathrm{Expresssion}}_{{\mathrm{all}}}} \right)} \right)}}{{{\mathrm{Mean}}\left( {{\mathrm{Expresssion}}_{{\mathrm{all}}}} \right)}}$$

which captures the normalized deviation of the gene expression in MDA-MB-231 from the average over the 675 cell lines that were reported in Klijn et al.^24^ Only those genes with *D* > 1 were included into the network construction (Supplementary Fig. 7). The topology of the signaling network was built by retrieving the interaction patterns (i.e., activation or inhibition) between the proteins that are connected to AURKB, ZAK, and CSF1R from OmniPath^23^ (Supplementary Data 12).

The simulation model has two main components: (i) a gene interaction mechanism; (ii) and a mechanism of cell growth and division. For the first component, the interactions between genes are modeled as a source to target reaction. We considered three types of interactions, namely activation, inhibition, and degradation. While activation and inhibition were inferred from OmniPath, the degradation was added to achieve a steady state gene expression levels. Examples depicting reactions for these interactions are:

9 $${\mathrm{A}}\mathop{\longrightarrow}\limits^{{{\mathrm{B}}k_{\mathrm{p}}}}{\mathrm{B}}\left( {{\mathrm{gene}}\;{\mathrm{A}}\;{\mathrm{activates}}\;{\mathrm{gene}}\;{\mathrm{B}}} \right)$$

10 $${\mathrm{B}} + {\mathrm{C}}\mathop{\longrightarrow}\limits^{{{\mathrm{C}}k_{\mathrm{d}}}}\emptyset \left( {{\mathrm{gene}}\;{\mathrm{B}}\;{\mathrm{inhibits}}\;{\mathrm{gene}}\;{\mathrm{C}}} \right)$$

11 $${\mathrm{A}}\mathop{\longrightarrow}\limits^{{{\mathrm{A}}k_{\mathrm{d}}}}\emptyset \left( {{\mathrm{gene}}\;{\mathrm{A}}\;{\mathrm{or}}\;{\mathrm{associated}}\;{\mathrm{mRNA}}\;{\mathrm{degrades}}\;{\mathrm{on}}\;{\mathrm{its}}\;{\mathrm{own}}} \right)$$

The propensities of each reaction are assumed to be driven by the gene expression profile of the source gene. To obtain the reported expression levels for each gene during simulation (Supplementary Data 13), we manually tuned the ratio of its degradation rate (*K*_*d*_) to production rate (*K*_p_) (Supplementary Data 16).

The second component of the model consists of cell growth and division. We modeled the division as an instantaneous event that occurs once the cell reaches a growth threshold, a variable that increases exponentially. We assumed that this growth threshold is affected by *TP53* and is modeled by:

12 $$\emptyset \mathop{\longrightarrow}\limits^{{{\mathrm{pGrowth}} \ast \left( {\frac{{{\mathrm{ln}}2}}{{t_{{\mathrm{div}}}}}} \right) \ast f({\mathrm{TP}}53)}}{\mathrm{pGrowth}}$$

The rate constant of this equation is such that in the absence or low expression of *TP53* expression, cells grow exponentially in time (*t*_div_) to match the doubling time of MDA-MB-231 cells. The effect of *TP53* on cell growth was modeled using a Hill function *f*(TP53), with the threshold *Q* = 65 and exponent *β* = 7, which regulate the degree of growth rate reduction when *TP53* is expressed.

It should be noted that on a division event, the components of the mother cell are partitioned between the daughter cells according to an unbiased, independent partitioning scheme (implemented in SGNS2^25^). This results in a binomial distribution of molecules inherited by resulting daughter cells.

### Dynamic simulation of the signaling network

Once the signaling model is constructed based on the network topology, the effect of knock-out of any genes was predicted using SGNS2,^25^ an open-source simulator of chemical reaction systems according to the SSA.^26^ To measure the viability of the cells in normal MDA-MB-231 model as well as in knock-out models, we counted the average number of viable cells during the last 50 h of simulation period from 10 runs. To further investigate the sensitivity of the parameters, we transformed the SGNS2 model to an ODE-based model using the same interactions and parameters. After verifying the accurate transformation using the steady state gene expression levels for genes in both models, we computed the sensitivities of each parameter using COPASI.^28^

### Reporting summary

Further information on research design is available in the Nature Research Reporting Summary linked to this article.

## Supplementary Information

Supplementary Figures 1–10

Reporting Summary

Supplementary Data 1

Supplementary Data 2

Supplementary Data 3

Supplementary Data 4

Supplementary Data 5

Supplementary Data 6

Supplementary Data 7

Supplementary Data 8

Supplementary Data 9

Supplementary Data 10

Supplementary Data 11

Supplementary Data 12

Supplementary Data 13

Supplementary Data 14

Supplementary Data 15

Supplementary Data 16

## Data Availability

All the data that are used for the study are available in the Supplementary Datasets 1–15.

## Supplementary Information

**Supplementary information** accompanies the paper on the *npj Systems Biology and Applications* website (10.1038/s41540-019-0098-z).

## Code availability

Supplementary Dataset 16 contains the Matlab and R codes for analyzing the drug sensitivity, drug-target interaction, and drug combination data, as well as the model files for the dynamic simulation using the SGNS2 tool (available at https://github.com/SGNS2).
