# Supplementary material for: Network pharmacology modeling identifies synergistic Aurora B and ZAK interaction in triple-negative breast cancer
Source: NPJ Syst Biol Appl. 2019 Jul 8;5:20. doi: 10.1038/s41540-019-0098-z (PMC6614366; doi:10.1038/s41540-019-0098-z)
Supplement: Supplementary file 1 — Supplementary Figures 1–10 [file 41540_2019_98_MOESM1_ESM.pdf]

Supplementary Figure 1. Dose-response matrices and Bliss drug synergy landscapes for the 50 drug pairs. The total number of drug combinations is 70 due to replicates.

Supplementary Figure 2. Histograms of the Bliss drug synergy scores, where the dasatinib-sorafenib combination is highlighted as a red bar.

Supplementary Figure 3. Interpretation of the network connectivity for the MDA-MB-231 drug combination network. (A) The predicted sensitivities for drug pairs that include BI 2536 (BI 2536 drug group) versus the others. (B) The predicted sensitivities for drug pairs that break the network (Group 1) versus the others (Group 2).

Supplementary Figure 4. %inhibition and %toxicity for *AURKB* siRNAs, *ZAK* siRNAs and their combinations. (A) Qiagen siRNAs and CellTiter-Glo readouts. (B) Ambion siRNAs and CellTiter-Glo readouts. (C) Ambion siRNAs and CellTox Green readouts. (D) The western blot gel images.

Supplementary Figure 5. %inhibition and %toxicity for *AURKB* sgRNAs, *ZAK* sgRNAs and sgRNA combinations in CRISPR/Cas9 screen.

Supplementary Figure 6. % inhibition and % toxicity for the Aurora B kinase inhibitors + *ZAK* siRNAs combination experiments for MDA-MB-361 and MDA-MB-436 cells.

Supplementary Figure 7. The RPKM values for the genes in the MDA-MB-231 specific signaling network, in relation to the distribution of all 675 cell lines reported in Klijn et al.[24].

Supplementary Figure 8. The ZAK and AURKB gene expression levels in TNBC versus non-TNBC cell lines from the Klijn et al. study[24].

Supplementary Figure 9. The Kaplan-Meier plot for the overall survival of TNBC patients with up-regulated AURKB expression (Z-score >2), down-regulated CSF1R (Z-score <0) and TP53 mutation (median months survival = 114, n = 17), as compared to the other TNBC patients (median

months survival not reached, n = 100). P-value = 0.68 by Fleming-Harrington test with weights p = 1 and q = 1.

Supplementary Figure 10. The Kaplan-Meier plot for the overall survival of TNBC patients with up-regulated MAP2K3 or MAPK11 (median months survival = 37.8, n = 20, Gene expression Z-score > 2) as compared to the other TNBC patients (median months survival = 114.1, n = 96).

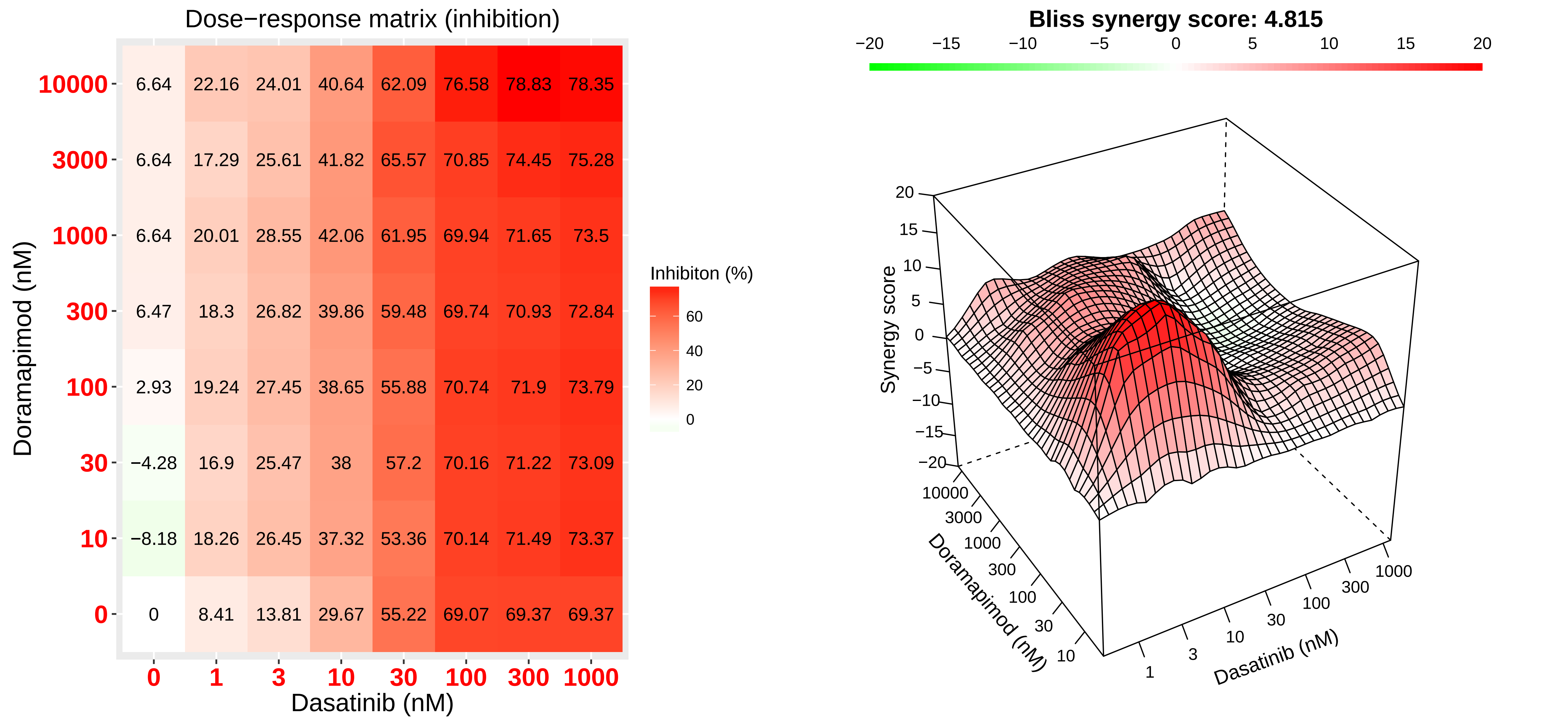
Dose−response matrix (inhibition)
10000 6.64 22.16 24.01 40.64 62.09 76.58 78.83 78.35
3000 6.64 17.29 25.61 41.82 65.57 70.85 74.45 75.28
1000 6.64 20.01 28.55 42.06 61.95 69.94 71.65 73.5
300 6.47 18.3 26.82 39.86 59.48 69.74 70.93 72.84
100 2.93 19.24 27.45 38.65 55.88 70.74 71.9 73.79
30 −4.28 16.9 25.47 38 57.2 70.16 71.22 73.09
10 −8.18 18.26 26.45 37.32 53.36 70.14 71.49 73.37
0 0 8.41 13.81 29.67 55.22 69.07 69.37 69.37
0 1 3 10 30 100 300 1000
Dasatinib (nM)
Doramapimod (nM)
Inhibiton (%)
60
40
20
0
Bliss synergy score: 4.815
−20 −15 −10 −5 0 5 10 15 20
Synergy score
20 15 10 5 0 −5 −10 −15 −20
Doramapimod (nM)
10000 3000 1000 300 100 30 10
Dasatinib (nM)
1 3 10 30 100 300 1000

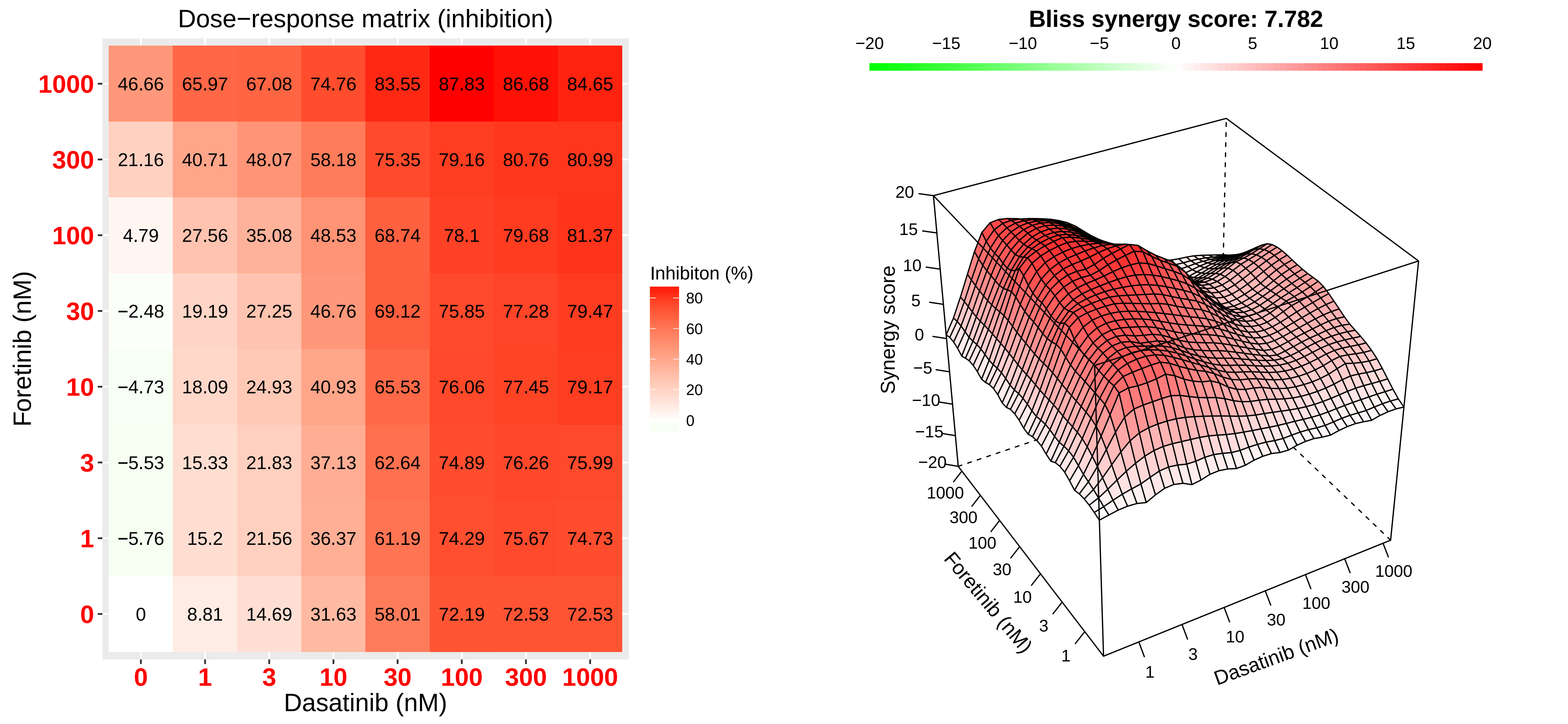
Dose−response matrix (inhibition)
1000 46.66 65.97 67.08 74.76 83.55 87.83 86.68 84.65
300 21.16 40.71 48.07 58.18 75.35 79.16 80.76 80.99
100 4.79 27.56 35.08 48.53 68.74 78.1 79.68 81.37
30 −2.48 19.19 27.25 46.76 69.12 75.85 77.28 79.47
10 −4.73 18.09 24.93 40.93 65.53 76.06 77.45 79.17
3 −5.53 15.33 21.83 37.13 62.64 74.89 76.26 75.99
1 −5.76 15.2 21.56 36.37 61.19 74.29 75.67 74.73
0 0 8.81 14.69 31.63 58.01 72.19 72.53 72.53
Foretinib (nM)
0 1 3 10 30 100 300 1000
Dasatinib (nM)
Inhibiton (%)
80
60
40
20
0
Bliss synergy score: 7.782
−20 −15 −10 −5 0 5 10 15 20
Synergy score
20 15 10 5 0 −5 −10 −15 −20
Foretinib (nM)
1000 300 100 30 10 3 1
Dasatinib (nM)
1 3 10 30 100 300 1000

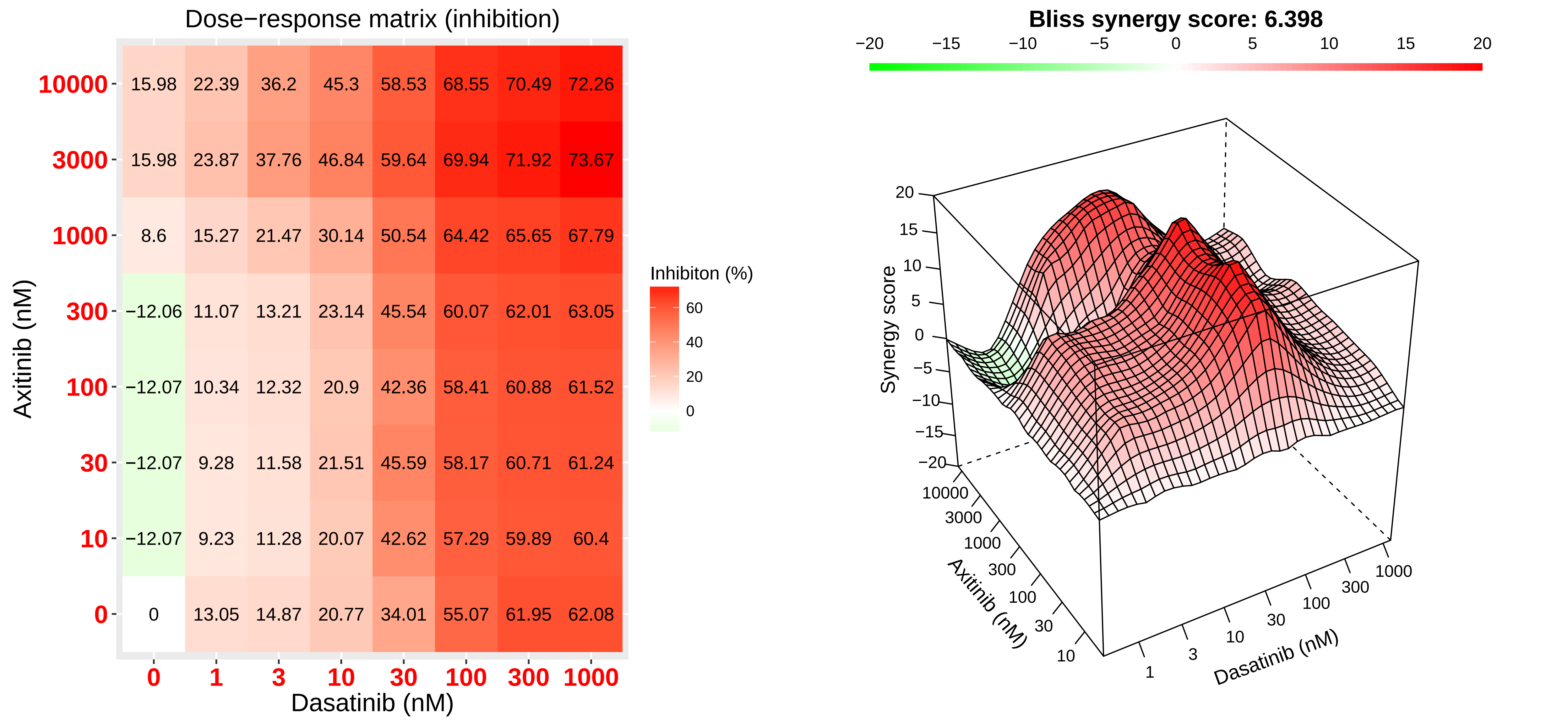
Dose−response matrix (inhibition)
10000 15.98 22.39 36.2 45.3 58.53 68.55 70.49 72.26
3000 15.98 23.87 37.76 46.84 59.64 69.94 71.92 73.67
1000 8.6 15.27 21.47 30.14 50.54 64.42 65.65 67.79
300 −12.06 11.07 13.21 23.14 45.54 60.07 62.01 63.05
100 −12.07 10.34 12.32 20.9 42.36 58.41 60.88 61.52
30 −12.07 9.28 11.58 21.51 45.59 58.17 60.71 61.24
10 −12.07 9.23 11.28 20.07 42.62 57.29 59.89 60.4
0 0 13.05 14.87 20.77 34.01 55.07 61.95 62.08
Axitinib (nM)
0 1 3 10 30 100 300 1000
Dasatinib (nM)
Inhibiton (%)
60
40
20
0
Bliss synergy score: 6.398
−20 −15 −10 −5 0 5 10 15 20
Synergy score
20 15 10 5 0 −5 −10 −15 −20
10000 3000 1000 300 100 30 10
Axitinib (nM)
1 3 10 30 100 300 1000
Dasatinib (nM)

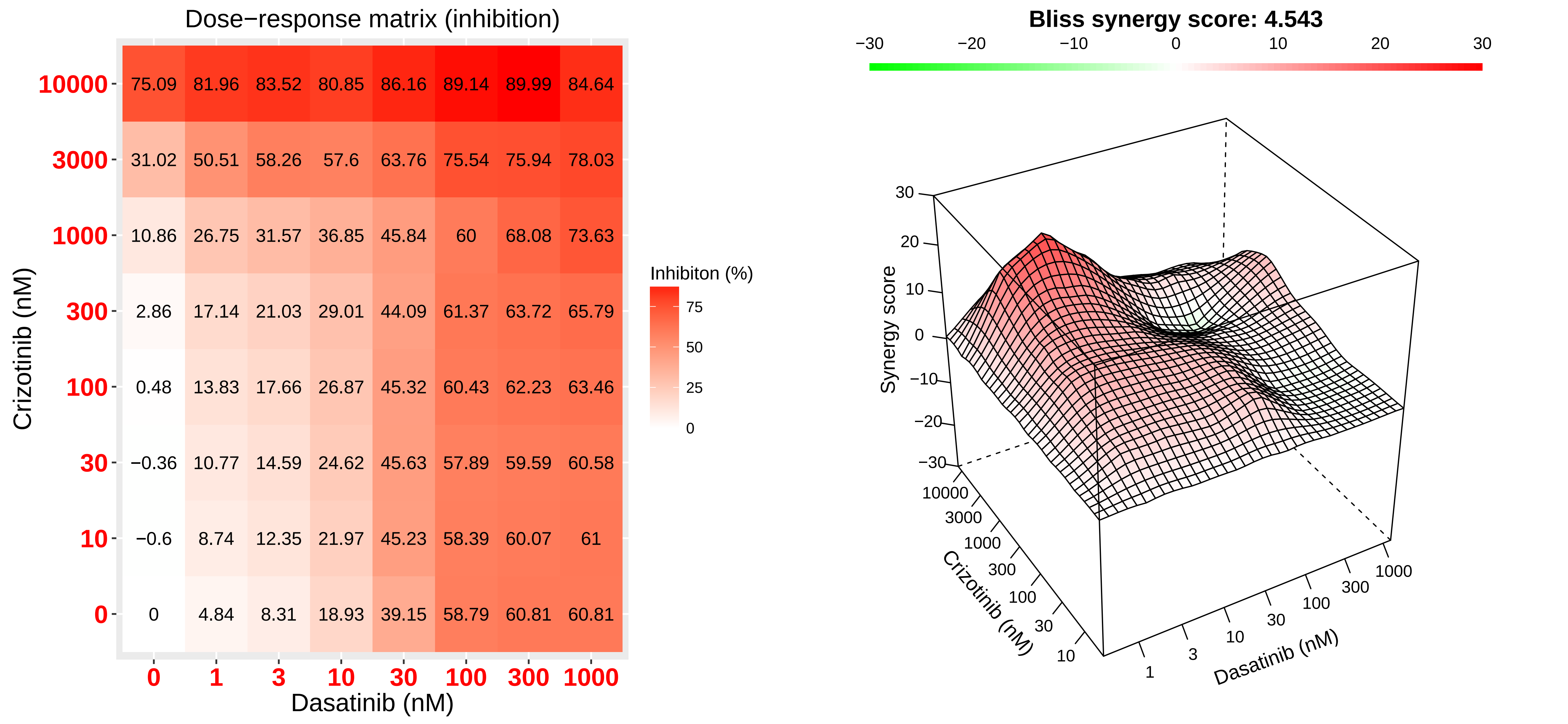

Dose−response matrix (inhibition)
10000
75.09 81.96 83.52 80.85 86.16 89.14 89.99 84.64
3000
31.02 50.51 58.26 57.6 63.76 75.54 75.94 78.03
1000
10.86 26.75 31.57 36.85 45.84 60 68.08 73.63
300
2.86 17.14 21.03 29.01 44.09 61.37 63.72 65.79
100
0.48 13.83 17.66 26.87 45.32 60.43 62.23 63.46
30
−0.36 10.77 14.59 24.62 45.63 57.89 59.59 60.58
10
−0.6 8.74 12.35 21.97 45.23 58.39 60.07 61
0
0 4.84 8.31 18.93 39.15 58.79 60.81 60.81
Crizotinib (nM)
0 1 3 10 30 100 300 1000
Dasatinib (nM)
Inhibiton (%)
75
50
25
0
Bliss synergy score: 4.543
−30 −20 −10 0 10 20 30
Synergy score
30 20 10 0 −10 −20 −30
10000 3000 1000 300 100 30 10
Crizotinib (nM)
1 3 10 30 100 300 1000
Dasatinib (nM)

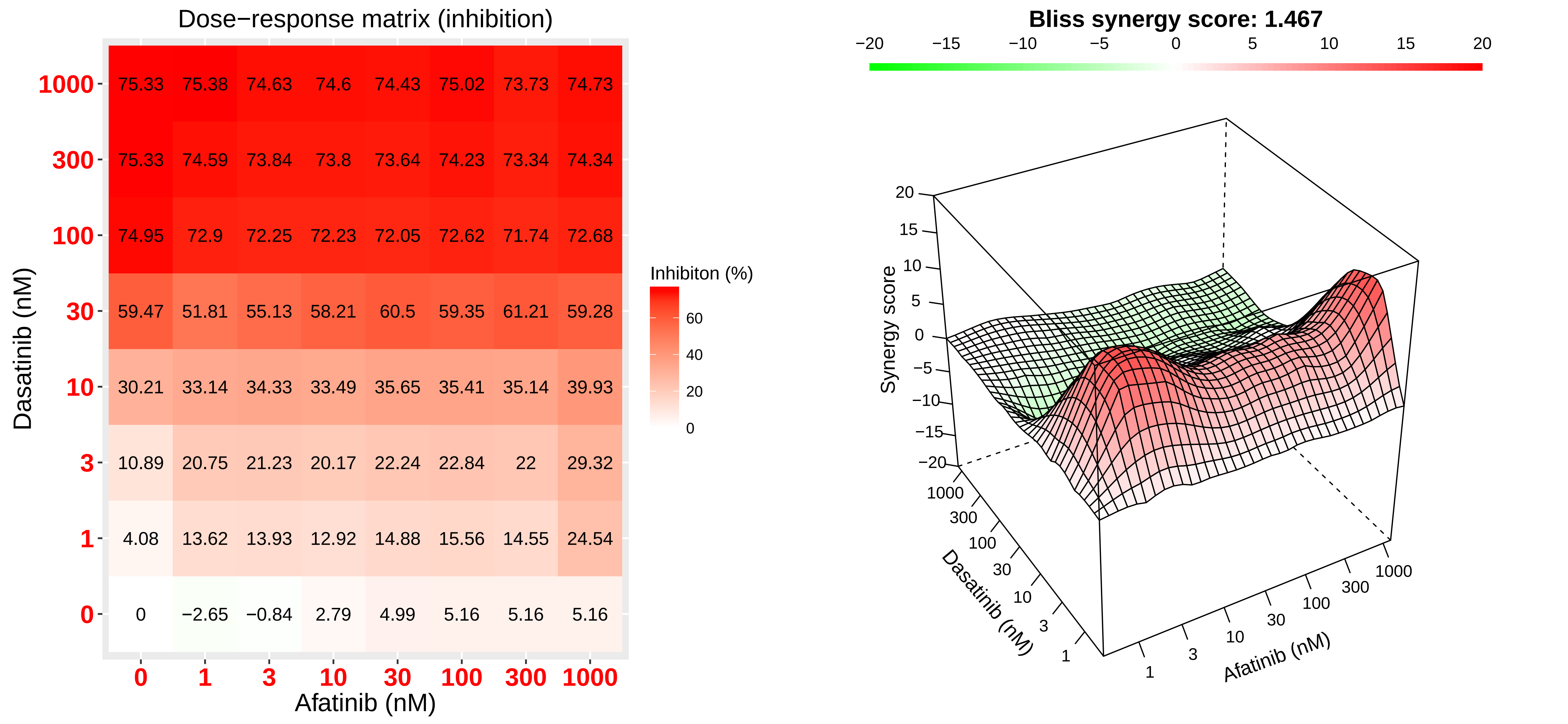

Dose−response matrix (inhibition)
1000 75.33 75.38 74.63 74.6 74.43 75.02 73.73 74.73
300 75.33 74.59 73.84 73.8 73.64 74.23 73.34 74.34
100 74.95 72.9 72.25 72.23 72.05 72.62 71.74 72.68
30 59.47 51.81 55.13 58.21 60.5 59.35 61.21 59.28
10 30.21 33.14 34.33 33.49 35.65 35.41 35.14 39.93
3 10.89 20.75 21.23 20.17 22.24 22.84 22 29.32
1 4.08 13.62 13.93 12.92 14.88 15.56 14.55 24.54
0 0 −2.65 −0.84 2.79 4.99 5.16 5.16 5.16
0 1 3 10 30 100 300 1000
Afatinib (nM)
Dasatinib (nM)
Inhibiton (%)
60
40
20
0
Bliss synergy score: 1.467
−20 −15 −10 −5 0 5 10 15 20
Synergy score
20 15 10 5 0 −5 −10 −15 −20
Dasatinib (nM)
1000 300 100 30 10 3 1
Afatinib (nM)
1 3 10 30 100 300 1000

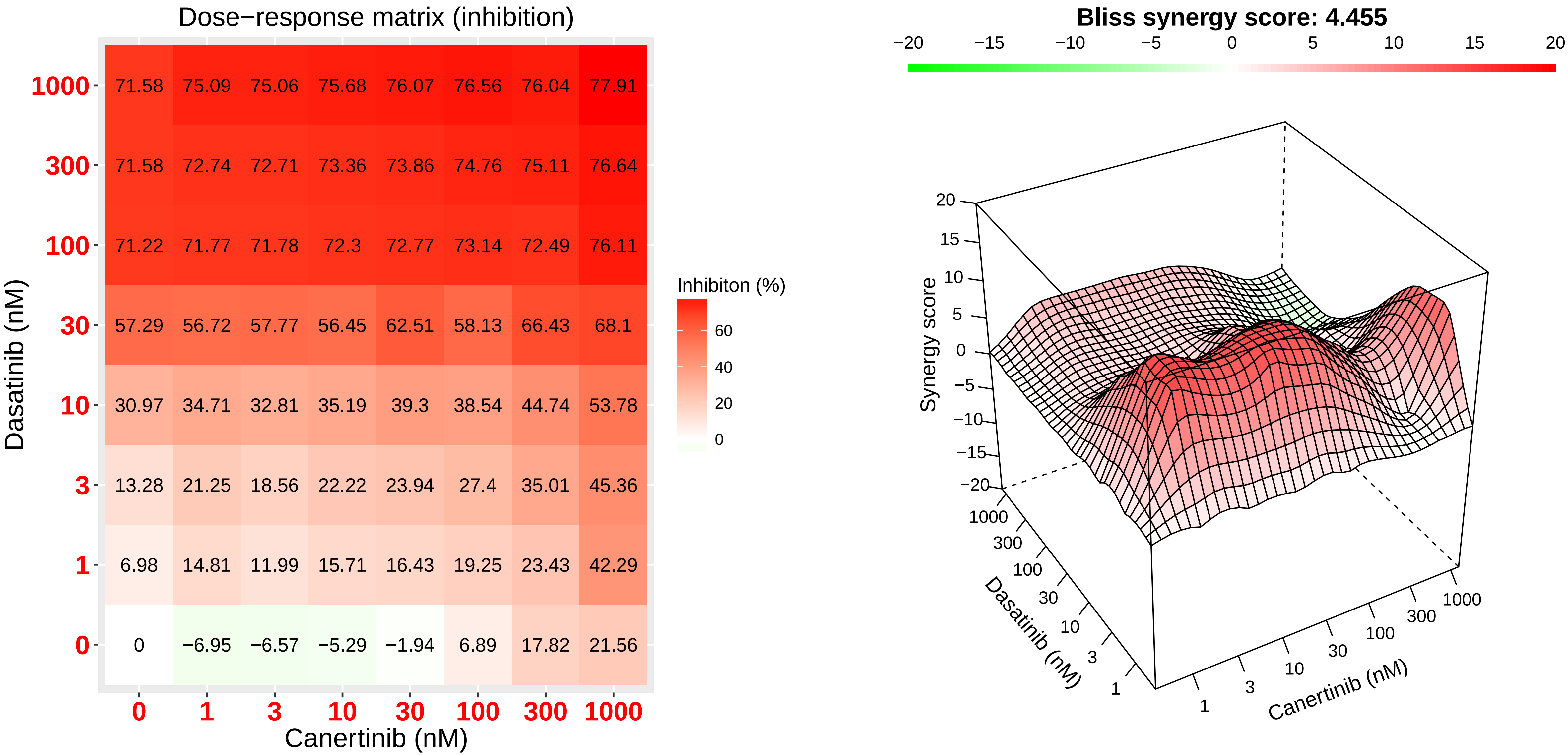
Dose−response matrix (inhibition)
Dasatinib (nM)
1000
71.58 75.09 75.06 75.68 76.07 76.56 76.04 77.91
300
71.58 72.74 72.71 73.36 73.86 74.76 75.11 76.64
100
71.22 71.77 71.78 72.3 72.77 73.14 72.49 76.11
30
57.29 56.72 57.77 56.45 62.51 58.13 66.43 68.1
10
30.97 34.71 32.81 35.19 39.3 38.54 44.74 53.78
3
13.28 21.25 18.56 22.22 23.94 27.4 35.01 45.36
1
6.98 14.81 11.99 15.71 16.43 19.25 23.43 42.29
0
0 −6.95 −6.57 −5.29 −1.94 6.89 17.82 21.56
0 1 3 10 30 100 300 1000
Canertinib (nM)
Inhibiton (%)
60
40
20
0
Bliss synergy score: 4.455
−20 −15 −10 −5 0 5 10 15 20
Synergy score
20
15
10
5
0
−5
−10
−15
−20
Dasatinib (nM)
1000
300
100
30
10
3
1
Canertinib (nM)
1
3
10
30
100
300
1000

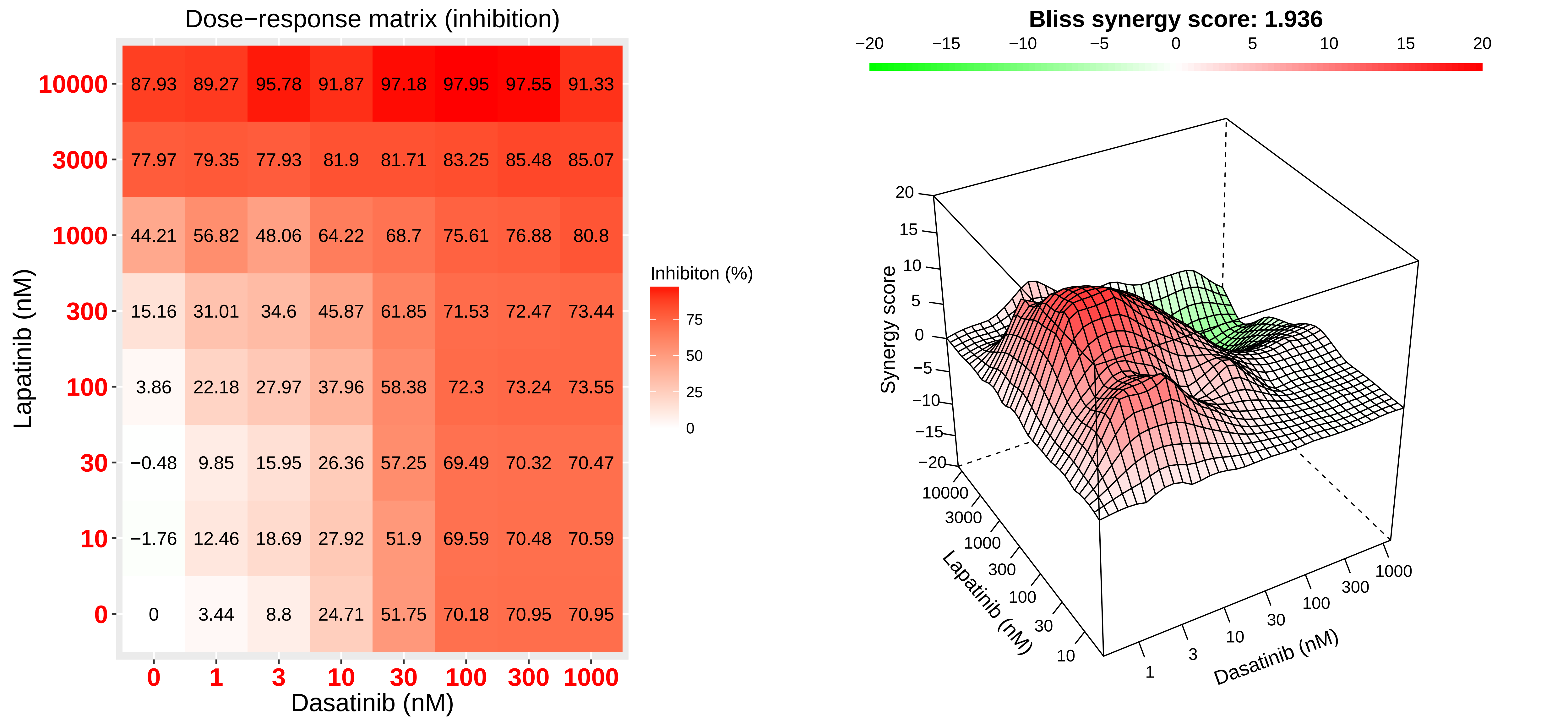
Dose−response matrix (inhibition)
Lapatinib (nM)
Dasatinib (nM)
0 1 3 10 30 100 300 1000
10000 87.93 89.27 95.78 91.87 97.18 97.95 97.55 91.33
3000 77.97 79.35 77.93 81.9 81.71 83.25 85.48 85.07
1000 44.21 56.82 48.06 64.22 68.7 75.61 76.88 80.8
300 15.16 31.01 34.6 45.87 61.85 71.53 72.47 73.44
100 3.86 22.18 27.97 37.96 58.38 72.3 73.24 73.55
30 −0.48 9.85 15.95 26.36 57.25 69.49 70.32 70.47
10 −1.76 12.46 18.69 27.92 51.9 69.59 70.48 70.59
0 0 3.44 8.8 24.71 51.75 70.18 70.95 70.95
Inhibiton (%)
0 25 50 75
Bliss synergy score: 1.936
−20 −15 −10 −5 0 5 10 15 20
Synergy score
20 15 10 5 0 −5 −10 −15 −20
Lapatinib (nM)
10000 3000 1000 300 100 30 10
Dasatinib (nM)
1 3 10 30 100 300 1000

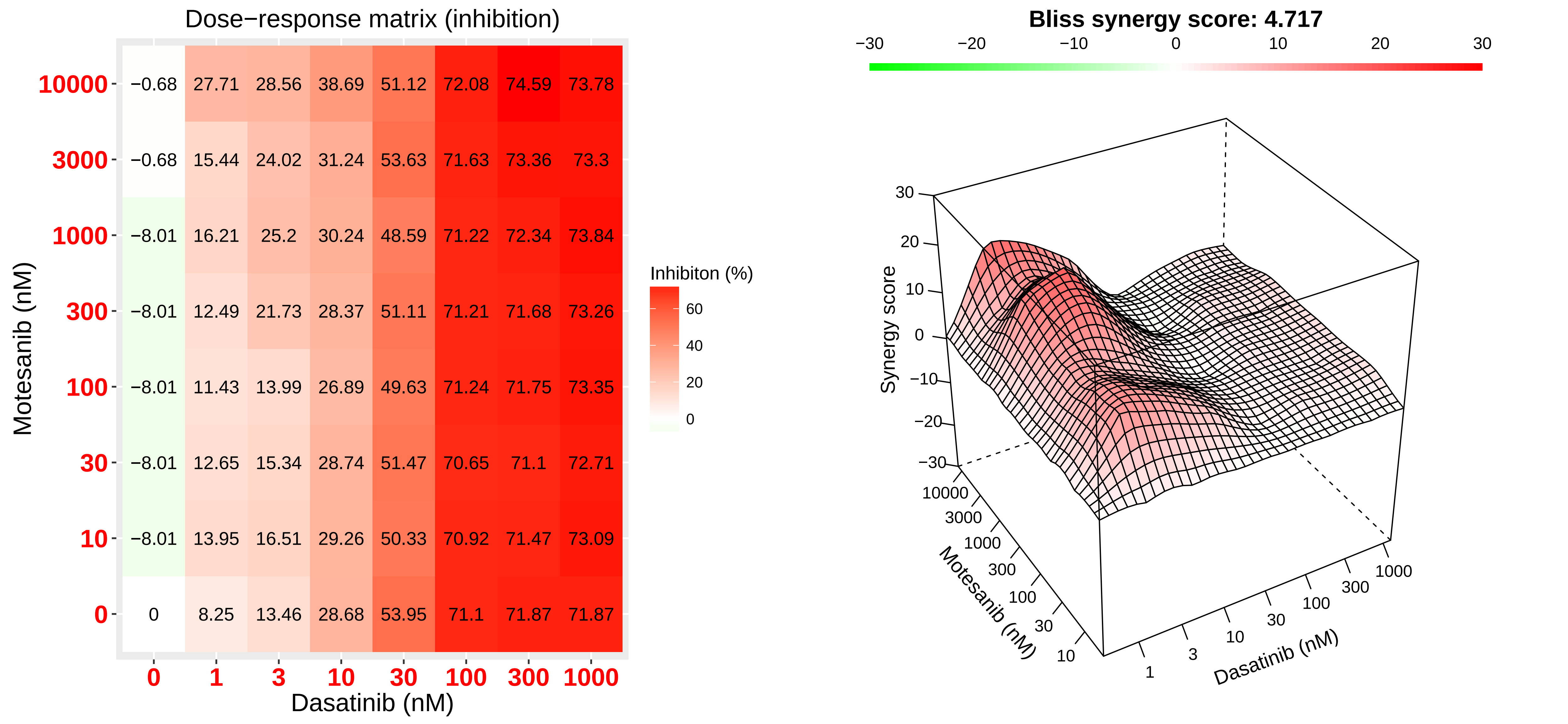

Dose−response matrix (inhibition)
10000 −0.68 27.71 28.56 38.69 51.12 72.08 74.59 73.78
3000 −0.68 15.44 24.02 31.24 53.63 71.63 73.36 73.3
1000 −8.01 16.21 25.2 30.24 48.59 71.22 72.34 73.84
300 −8.01 12.49 21.73 28.37 51.11 71.21 71.68 73.26
100 −8.01 11.43 13.99 26.89 49.63 71.24 71.75 73.35
30 −8.01 12.65 15.34 28.74 51.47 70.65 71.1 72.71
10 −8.01 13.95 16.51 29.26 50.33 70.92 71.47 73.09
0 0 8.25 13.46 28.68 53.95 71.1 71.87 71.87
0 1 3 10 30 100 300 1000
Dasatinib (nM)
Motesanib (nM)
Inhibiton (%)
60
40
20
0
Bliss synergy score: 4.717
−30 −20 −10 0 10 20 30
Synergy score
30
20
10
0
−10
−20
−30
10000
3000
1000
300
100
30
10
Motesanib (nM)
1
3
10
30
100
300
1000
Dasatinib (nM)

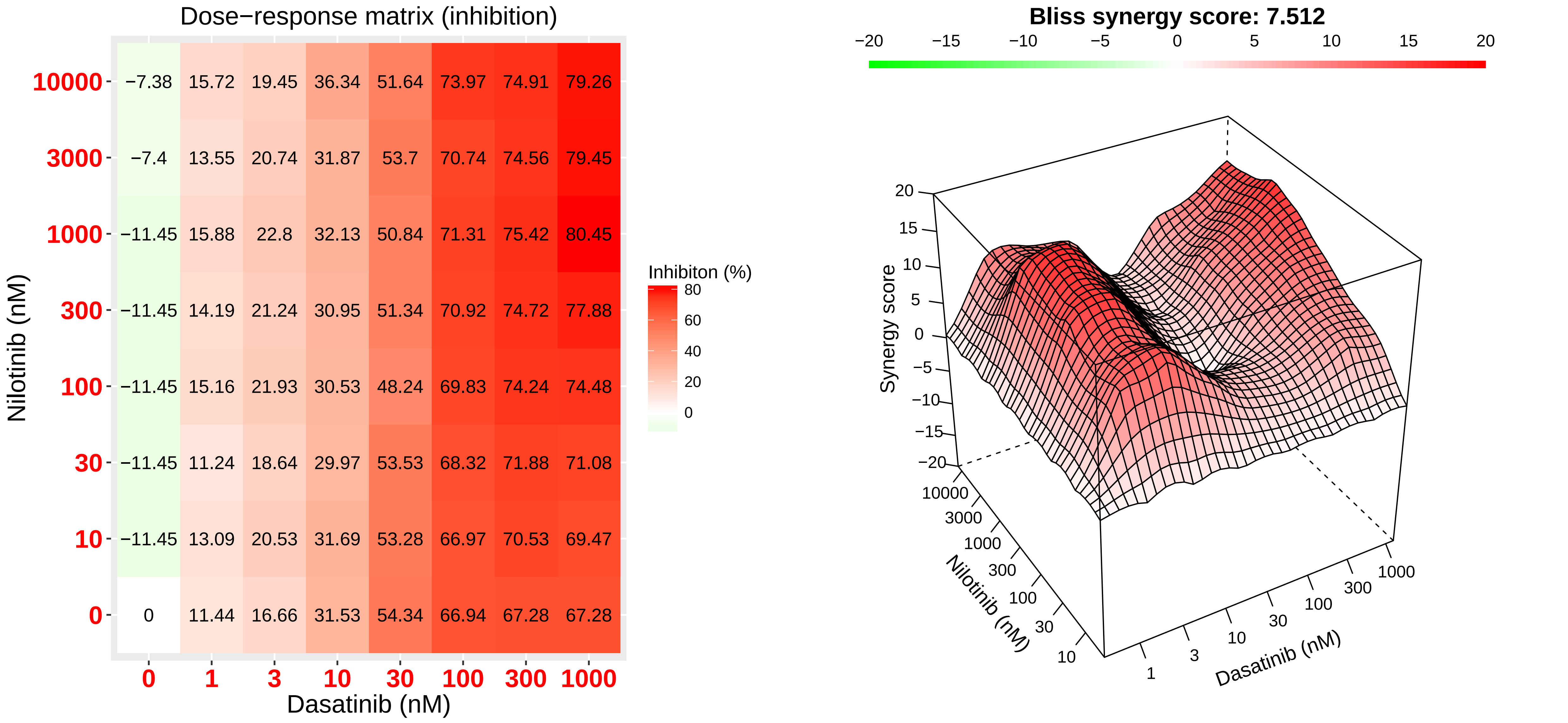
Dose−response matrix (inhibition)
10000 −7.38 15.72 19.45 36.34 51.64 73.97 74.91 79.26
3000 −7.4 13.55 20.74 31.87 53.7 70.74 74.56 79.45
1000 −11.45 15.88 22.8 32.13 50.84 71.31 75.42 80.45
300 −11.45 14.19 21.24 30.95 51.34 70.92 74.72 77.88
100 −11.45 15.16 21.93 30.53 48.24 69.83 74.24 74.48
30 −11.45 11.24 18.64 29.97 53.53 68.32 71.88 71.08
10 −11.45 13.09 20.53 31.69 53.28 66.97 70.53 69.47
0 0 11.44 16.66 31.53 54.34 66.94 67.28 67.28
0 1 3 10 30 100 300 1000
Dasatinib (nM)
Nilotinib (nM)
Inhibiton (%)
80
60
40
20
0
Bliss synergy score: 7.512
−20 −15 −10 −5 0 5 10 15 20
Synergy score
20
15
10
5
0
−5
−10
−15
−20
10000
3000
1000
300
100
30
10
Nilotinib (nM)
1
3
10
30
100
300
1000
Dasatinib (nM)

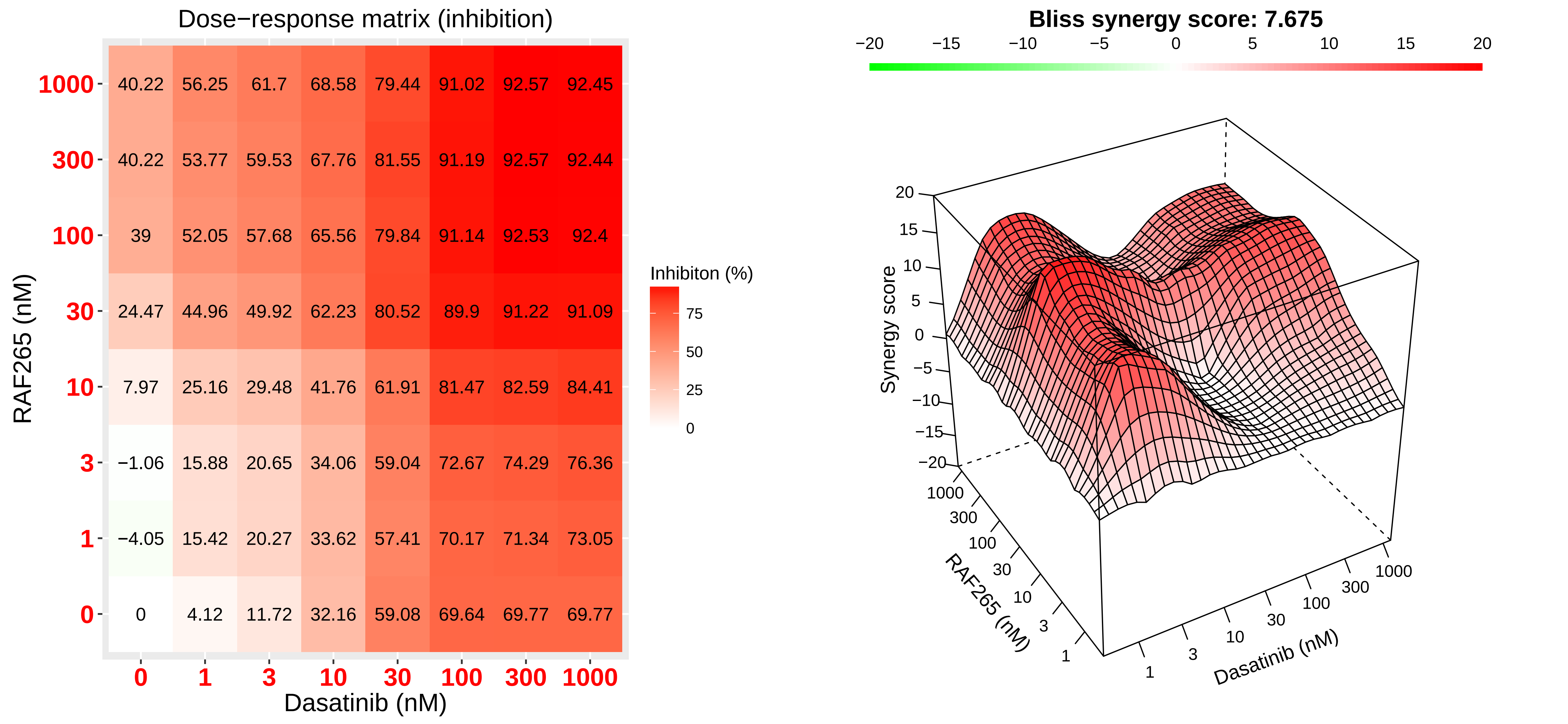
Dose−response matrix (inhibition)
1000 40.22 56.25 61.7 68.58 79.44 91.02 92.57 92.45
300 40.22 53.77 59.53 67.76 81.55 91.19 92.57 92.44
100 39 52.05 57.68 65.56 79.84 91.14 92.53 92.4
30 24.47 44.96 49.92 62.23 80.52 89.9 91.22 91.09
10 7.97 25.16 29.48 41.76 61.91 81.47 82.59 84.41
3 −1.06 15.88 20.65 34.06 59.04 72.67 74.29 76.36
1 −4.05 15.42 20.27 33.62 57.41 70.17 71.34 73.05
0 0 4.12 11.72 32.16 59.08 69.64 69.77 69.77
0 1 3 10 30 100 300 1000
RAF265 (nM)
Dasatinib (nM)
Inhibiton (%)
75
50
25
0
Bliss synergy score: 7.675
−20 −15 −10 −5 0 5 10 15 20
Synergy score
20 15 10 5 0 −5 −10 −15 −20
RAF265 (nM)
1000 300 100 30 10 3 1
Dasatinib (nM)
1 3 10 30 100 300 1000

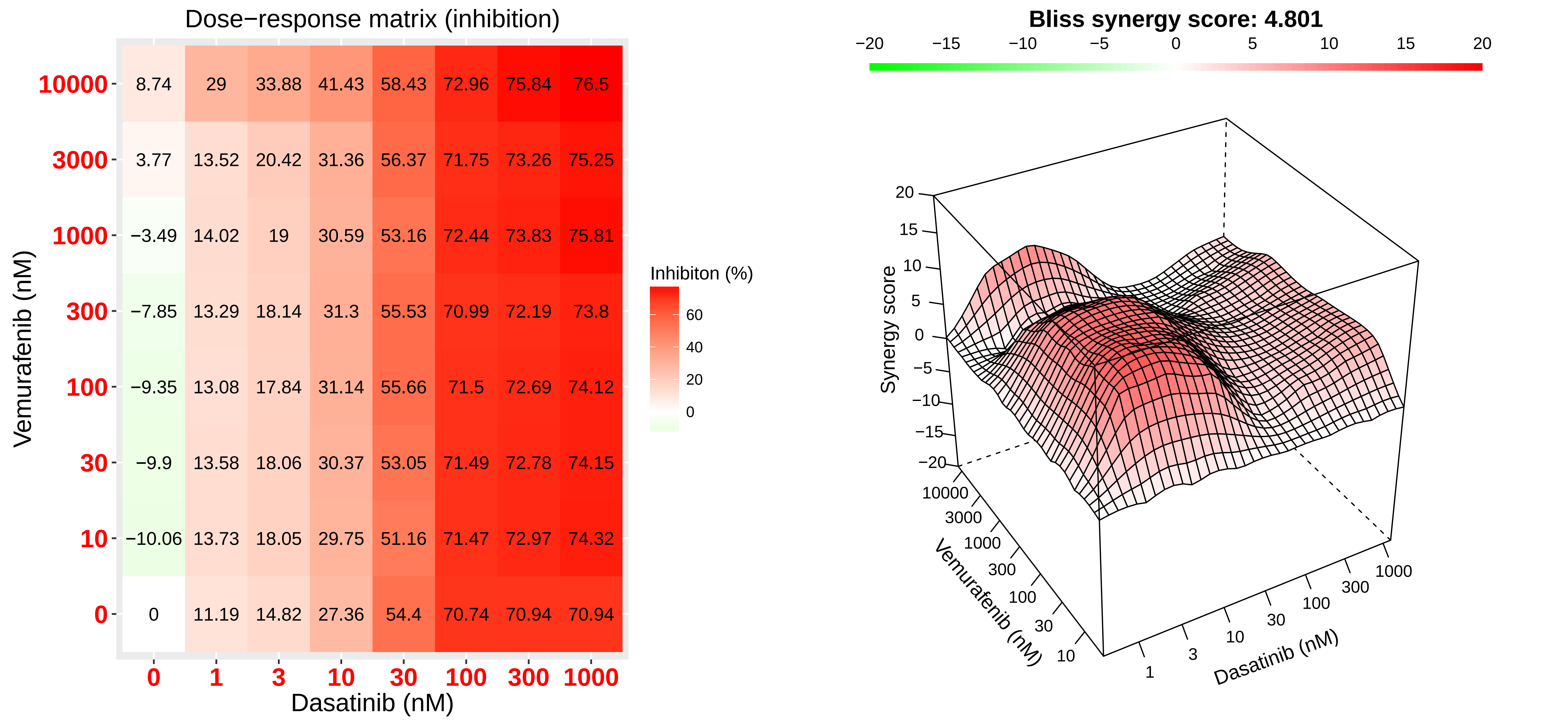

Dose−response matrix (inhibition)
10000
3000
1000
300
100
30
10
0
Vemurafenib (nM)
8.74 29 33.88 41.43 58.43 72.96 75.84 76.5
3.77 13.52 20.42 31.36 56.37 71.75 73.26 75.25
−3.49 14.02 19 30.59 53.16 72.44 73.83 75.81
−7.85 13.29 18.14 31.3 55.53 70.99 72.19 73.8
−9.35 13.08 17.84 31.14 55.66 71.5 72.69 74.12
−9.9 13.58 18.06 30.37 53.05 71.49 72.78 74.15
−10.06 13.73 18.05 29.75 51.16 71.47 72.97 74.32
0 11.19 14.82 27.36 54.4 70.74 70.94 70.94
0 1 3 10 30 100 300 1000
Dasatinib (nM)
Inhibiton (%)
60
40
20
0
Bliss synergy score: 4.801
−20 −15 −10 −5 0 5 10 15 20
Synergy score
20
15
10
5
0
−5
−10
−15
−20
10000
3000
1000
300
100
30
10
Vemurafenib (nM)
1
3
10
30
100
300
1000
Dasatinib (nM)

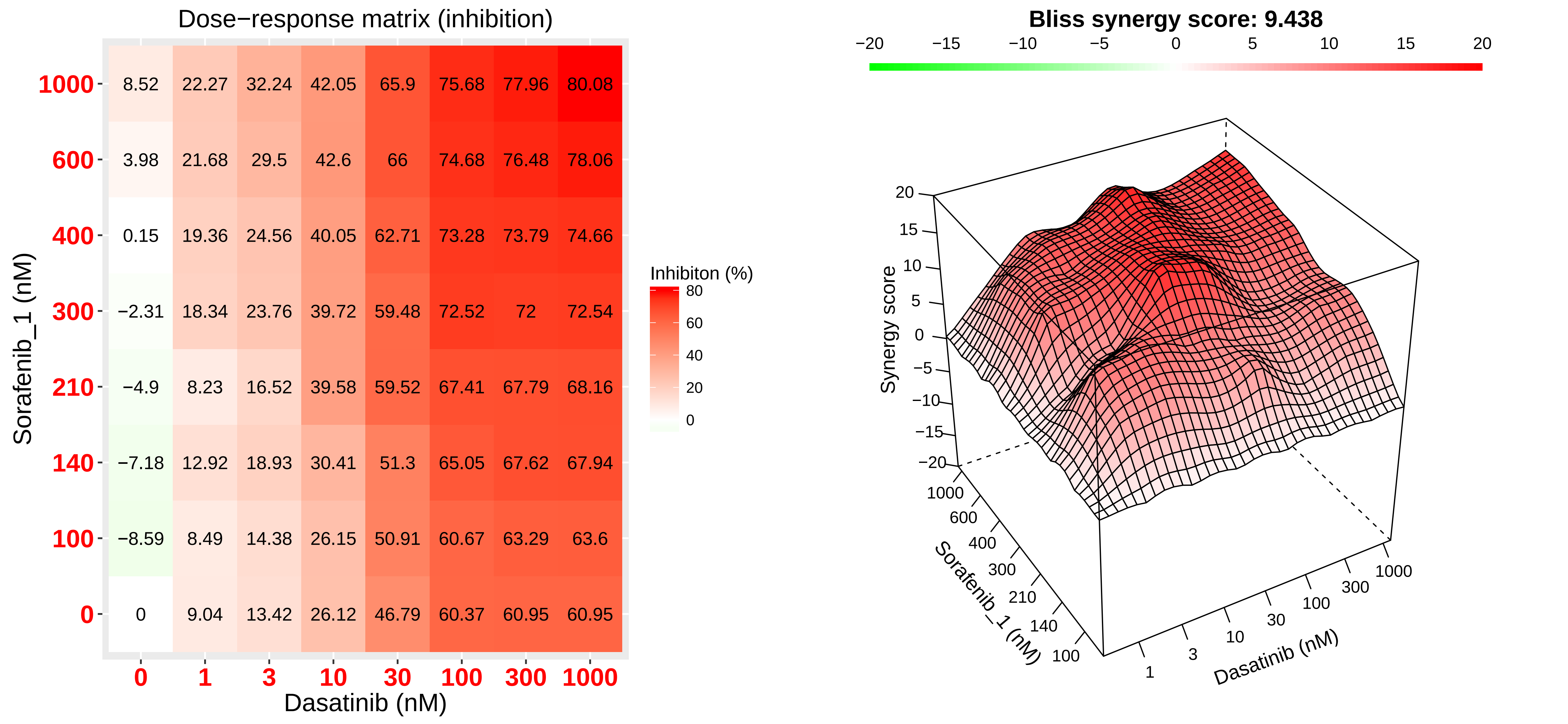
Dose−response matrix (inhibition)
1000
8.52 22.27 32.24 42.05 65.9 75.68 77.96 80.08
600
3.98 21.68 29.5 42.6 66 74.68 76.48 78.06
400
0.15 19.36 24.56 40.05 62.71 73.28 73.79 74.66
300
−2.31 18.34 23.76 39.72 59.48 72.52 72 72.54
210
−4.9 8.23 16.52 39.58 59.52 67.41 67.79 68.16
140
−7.18 12.92 18.93 30.41 51.3 65.05 67.62 67.94
100
−8.59 8.49 14.38 26.15 50.91 60.67 63.29 63.6
0
0 9.04 13.42 26.12 46.79 60.37 60.95 60.95
Sorafenib_1 (nM)
0 1 3 10 30 100 300 1000
Dasatinib (nM)
Inhibiton (%)
80
60
40
20
0
Bliss synergy score: 9.438
−20 −15 −10 −5 0 5 10 15 20
Synergy score
20
15
10
5
0
−5
−10
−15
−20
1000
600
400
300
210
140
100
Sorafenib_1 (nM)
1
3
10
30
100
300
1000
Dasatinib (nM)

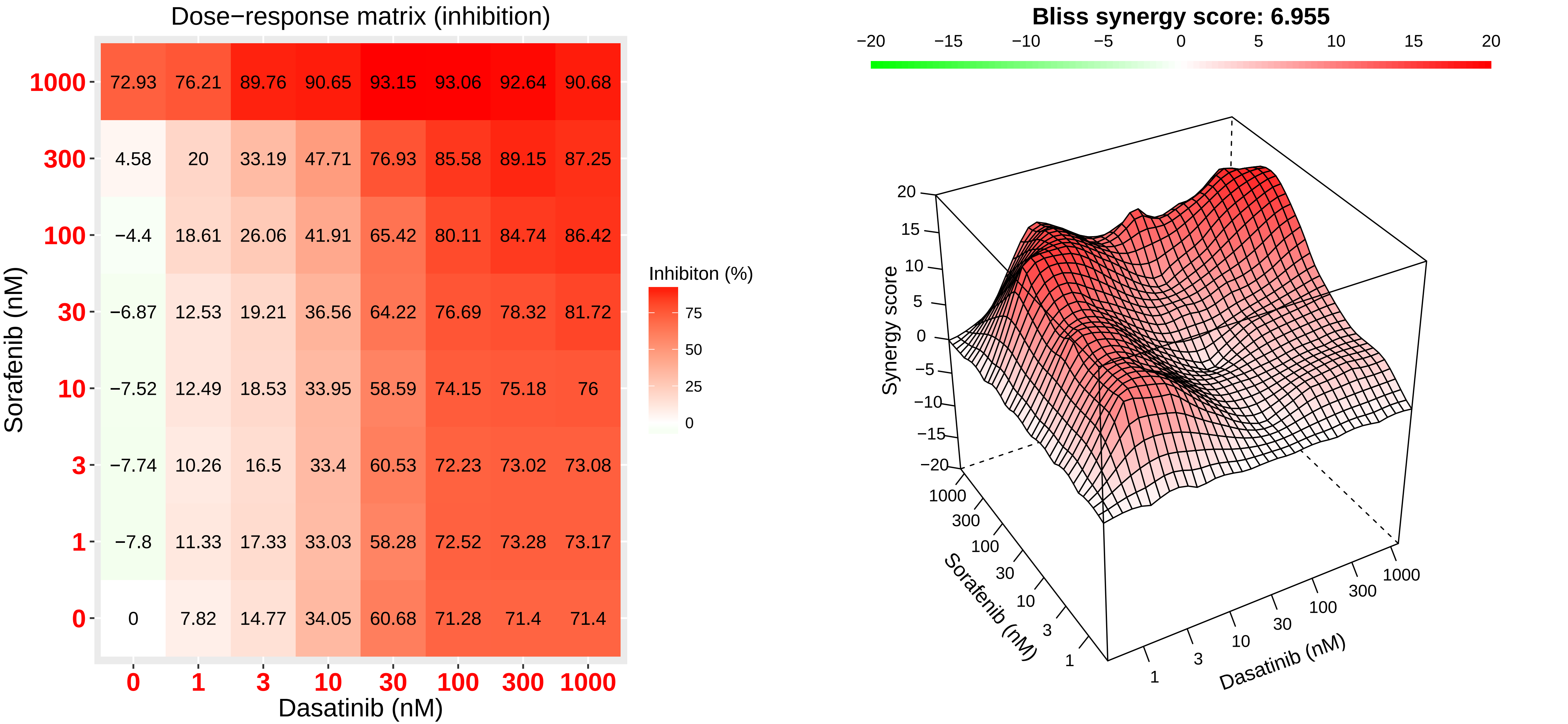

Dose−response matrix (inhibition)
1000
300
100
30
10
3
1
0
72.93 76.21 89.76 90.65 93.15 93.06 92.64 90.68
4.58 20 33.19 47.71 76.93 85.58 89.15 87.25
−4.4 18.61 26.06 41.91 65.42 80.11 84.74 86.42
−6.87 12.53 19.21 36.56 64.22 76.69 78.32 81.72
−7.52 12.49 18.53 33.95 58.59 74.15 75.18 76
−7.74 10.26 16.5 33.4 60.53 72.23 73.02 73.08
−7.8 11.33 17.33 33.03 58.28 72.52 73.28 73.17
0 7.82 14.77 34.05 60.68 71.28 71.4 71.4
Sorafenib (nM)
0 1 3 10 30 100 300 1000
Dasatinib (nM)
Inhibiton (%)
75
50
25
0
Bliss synergy score: 6.955
−20 −15 −10 −5 0 5 10 15 20
Synergy score
20
15
10
5
0
−5
−10
−15
−20
1000
300
100
30
10
3
1
Sorafenib (nM)
1 3 10 30 100 300 1000
Dasatinib (nM)

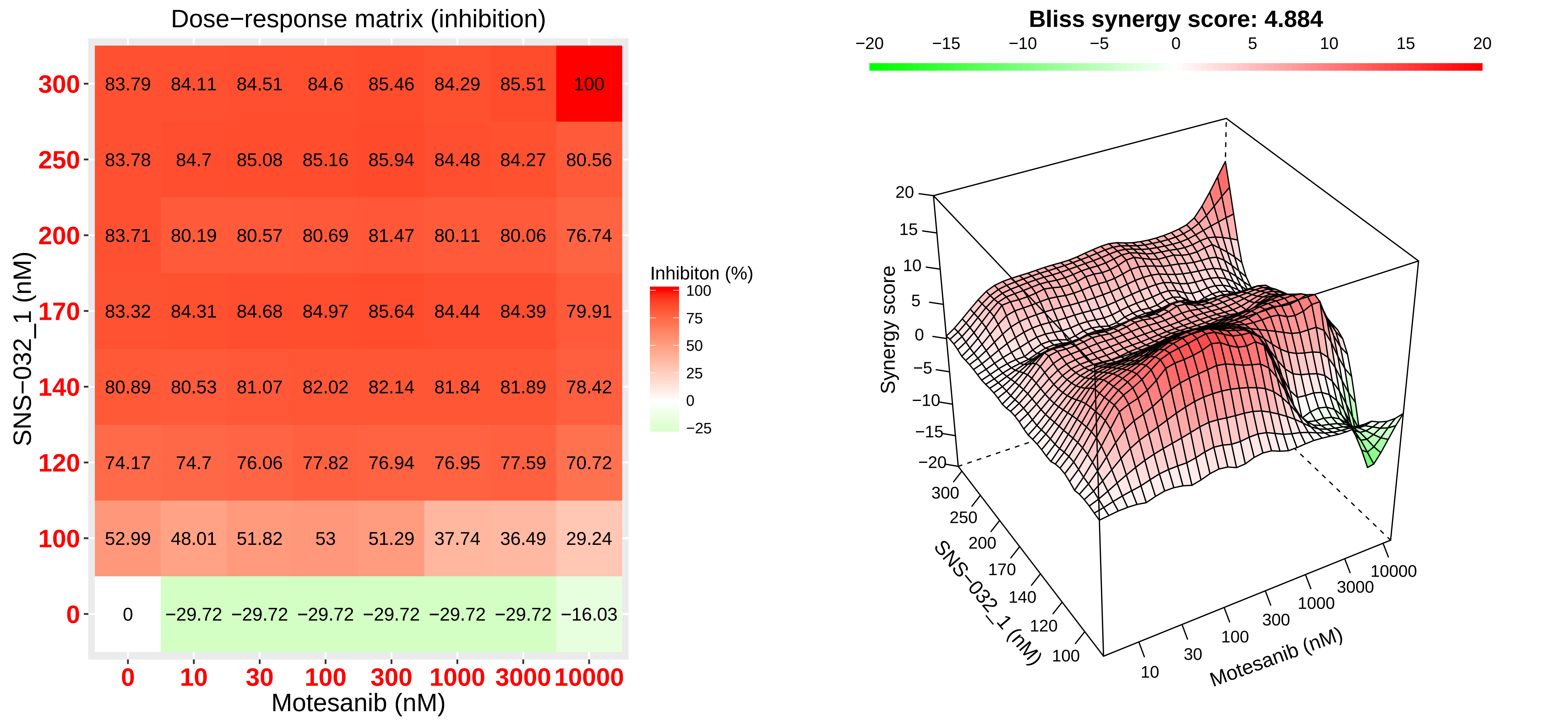
Dose−response matrix (inhibition)
300
83.79 84.11 84.51 84.6 85.46 84.29 85.51 100
250
83.78 84.7 85.08 85.16 85.94 84.48 84.27 80.56
200
83.71 80.19 80.57 80.69 81.47 80.11 80.06 76.74
170
83.32 84.31 84.68 84.97 85.64 84.44 84.39 79.91
140
80.89 80.53 81.07 82.02 82.14 81.84 81.89 78.42
120
74.17 74.7 76.06 77.82 76.94 76.95 77.59 70.72
100
52.99 48.01 51.82 53 51.29 37.74 36.49 29.24
0
0 −29.72 −29.72 −29.72 −29.72 −29.72 −29.72 −16.03
SNS−032_1 (nM)
0 10 30 100 300 1000 3000 10000
Motesanib (nM)
Inhibiton (%)
100
75
50
25
0
−25
Bliss synergy score: 4.884
−20 −15 −10 −5 0 5 10 15 20
Synergy score
20 15 10 5 0 −5 −10 −15 −20
300 250 200 170 140 120 100
SNS−032_1 (nM)
10 30 100 300 1000 3000 10000
Motesanib (nM)

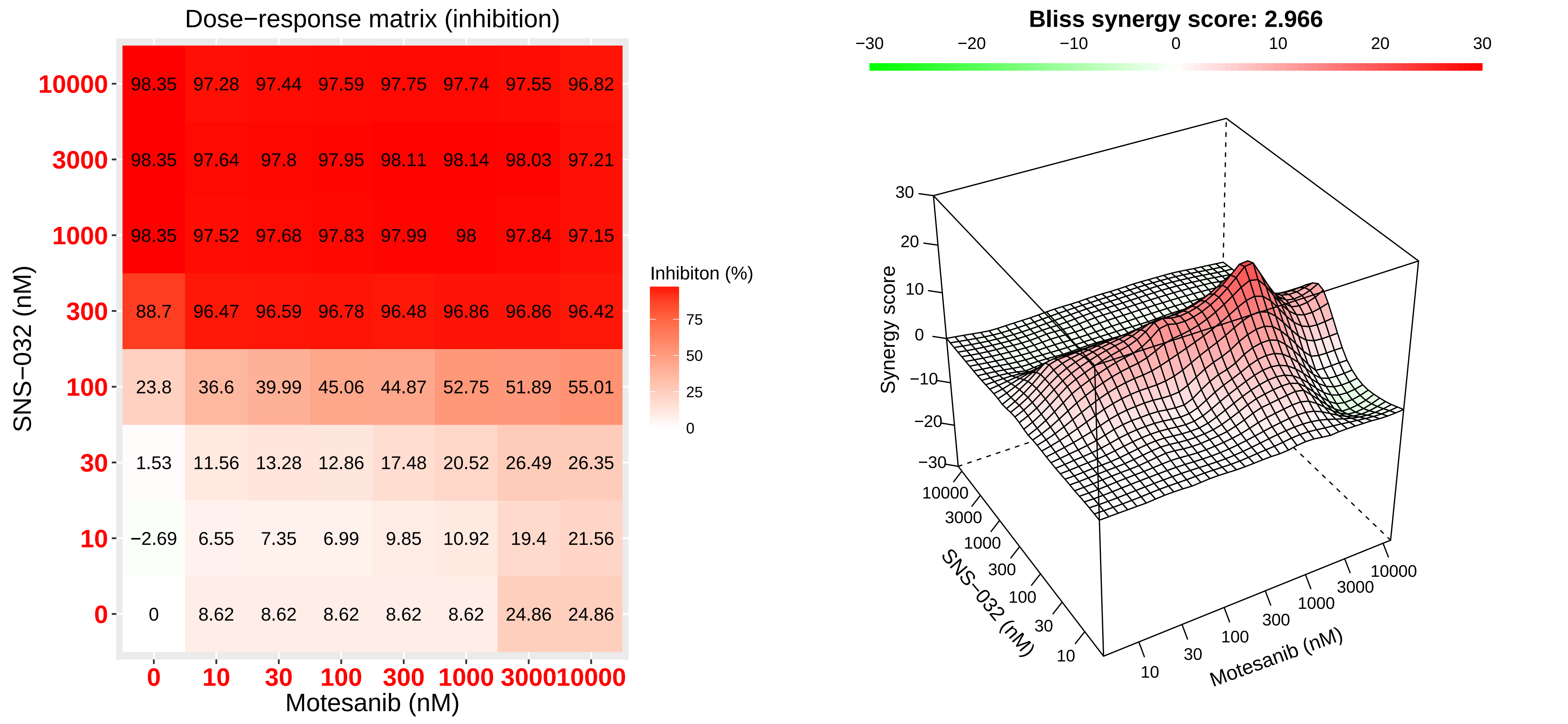
Dose−response matrix (inhibition)
10000 98.35 97.28 97.44 97.59 97.75 97.74 97.55 96.82
3000 98.35 97.64 97.8 97.95 98.11 98.14 98.03 97.21
1000 98.35 97.52 97.68 97.83 97.99 98 97.84 97.15
300 88.7 96.47 96.59 96.78 96.48 96.86 96.86 96.42
100 23.8 36.6 39.99 45.06 44.87 52.75 51.89 55.01
30 1.53 11.56 13.28 12.86 17.48 20.52 26.49 26.35
10 −2.69 6.55 7.35 6.99 9.85 10.92 19.4 21.56
0 0 8.62 8.62 8.62 8.62 8.62 24.86 24.86
0 10 30 100 300 1000 3000 10000
Motesanib (nM)
SNS−032 (nM)
Inhibiton (%)
75
50
25
0
Bliss synergy score: 2.966
−30 −20 −10 0 10 20 30
Synergy score
30
20
10
0
−10
−20
−30
10000 3000 1000 300 100 30 10
SNS−032 (nM)
10 30 100 300 1000 3000 10000
Motesanib (nM)

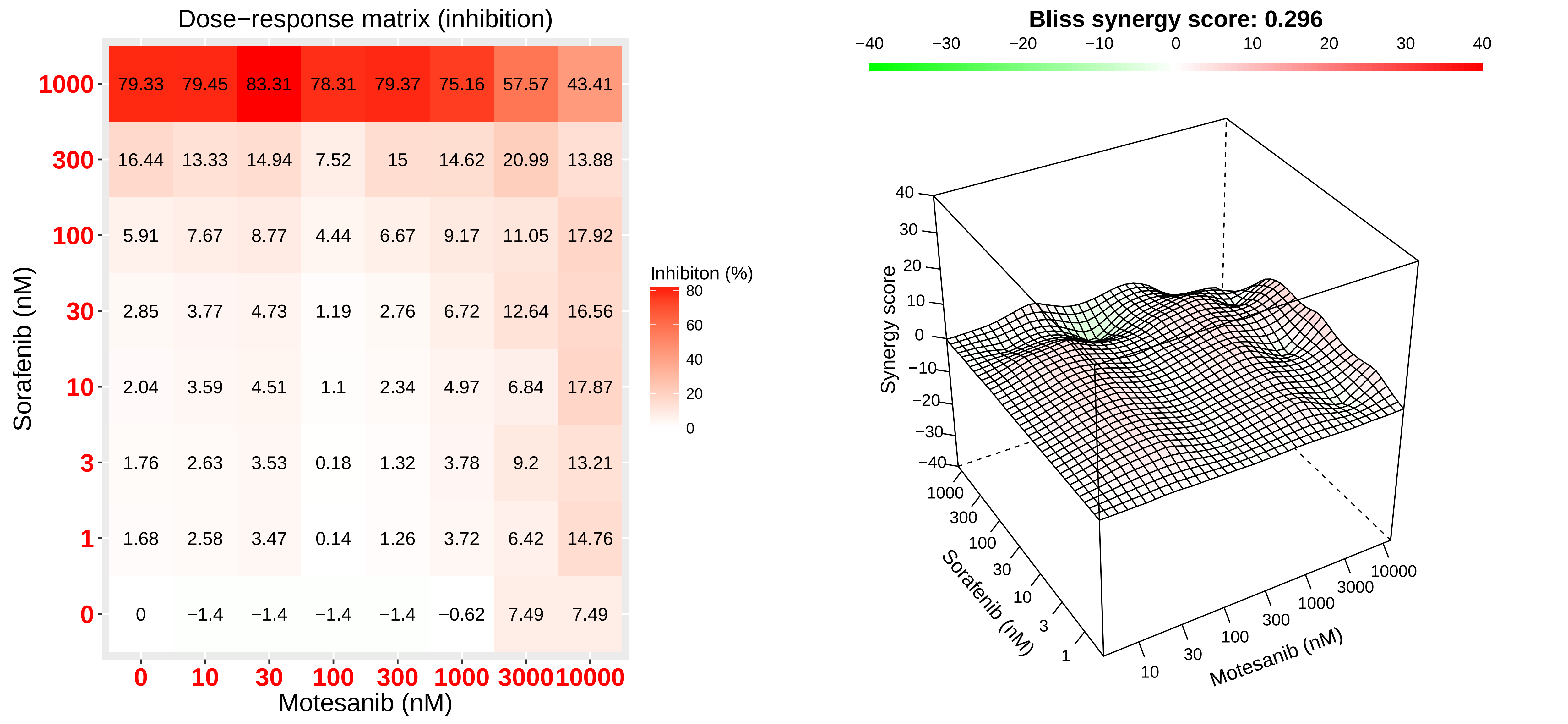
Dose−response matrix (inhibition)
1000
79.33 79.45 83.31 78.31 79.37 75.16 57.57 43.41
300
16.44 13.33 14.94 7.52 15 14.62 20.99 13.88
100
5.91 7.67 8.77 4.44 6.67 9.17 11.05 17.92
30
2.85 3.77 4.73 1.19 2.76 6.72 12.64 16.56
10
2.04 3.59 4.51 1.1 2.34 4.97 6.84 17.87
3
1.76 2.63 3.53 0.18 1.32 3.78 9.2 13.21
1
1.68 2.58 3.47 0.14 1.26 3.72 6.42 14.76
0
0 −1.4 −1.4 −1.4 −1.4 −0.62 7.49 7.49
Sorafenib (nM)
0 10 30 100 300 1000 3000 10000
Motesanib (nM)
Inhibiton (%)
80
60
40
20
0
Bliss synergy score: 0.296
−40 −30 −20 −10 0 10 20 30 40
Synergy score
40 30 20 10 0 −10 −20 −30 −40
1000 300 100 30 10 3 1
Sorafenib (nM)
10 30 100 300 1000 3000 10000
Motesanib (nM)

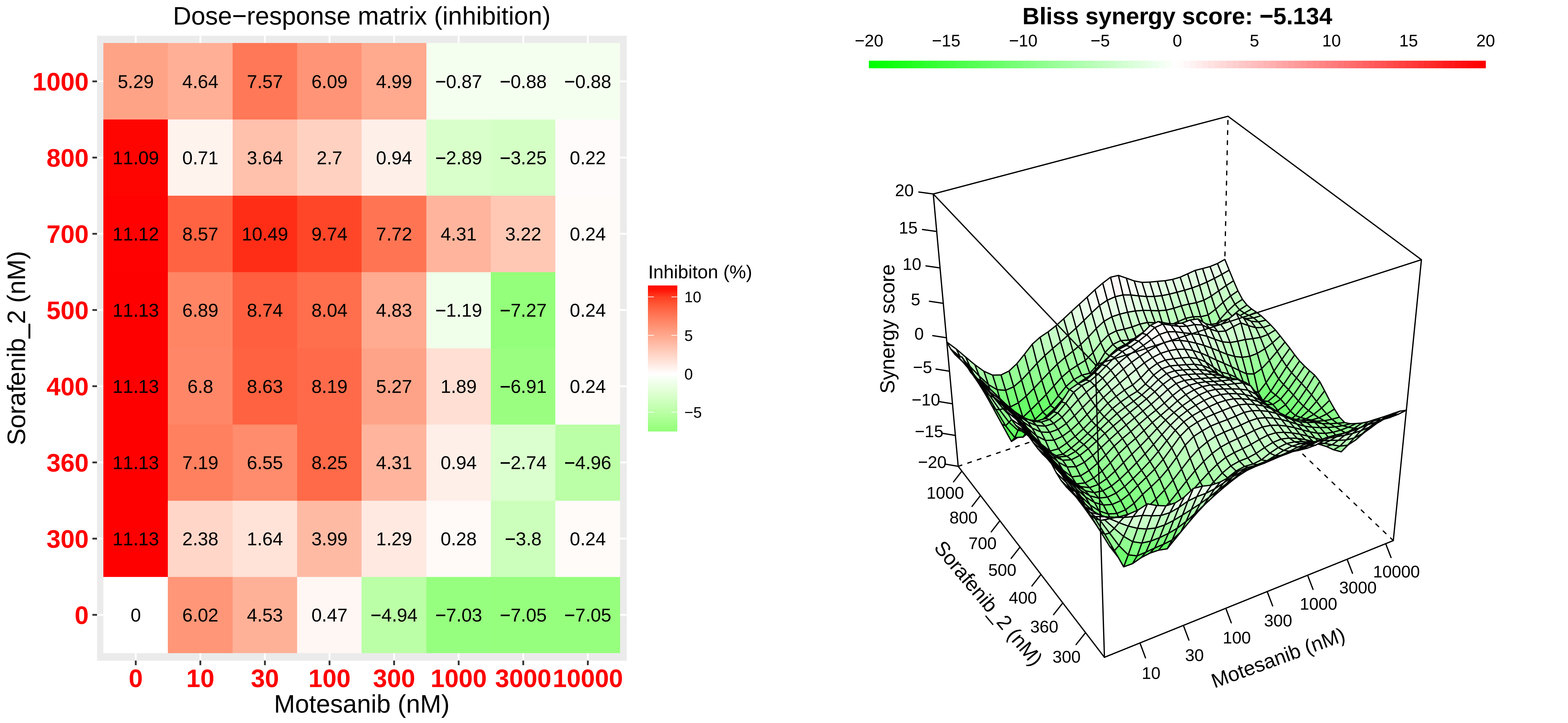
Dose−response matrix (inhibition)
1000
5.29 4.64 7.57 6.09 4.99 −0.87 −0.88 −0.88
800
11.09 0.71 3.64 2.7 0.94 −2.89 −3.25 0.22
700
11.12 8.57 10.49 9.74 7.72 4.31 3.22 0.24
500
11.13 6.89 8.74 8.04 4.83 −1.19 −7.27 0.24
400
11.13 6.8 8.63 8.19 5.27 1.89 −6.91 0.24
360
11.13 7.19 6.55 8.25 4.31 0.94 −2.74 −4.96
300
11.13 2.38 1.64 3.99 1.29 0.28 −3.8 0.24
0
0 6.02 4.53 0.47 −4.94 −7.03 −7.05 −7.05
Sorafenib_2 (nM)
0 10 30 100 300 1000 3000 10000
Motesanib (nM)
Inhibiton (%)
10
5
0
−5
Bliss synergy score: −5.134
−20 −15 −10 −5 0 5 10 15 20
Synergy score
20
15
10
5
0
−5
−10
−15
−20
1000
800
700
500
400
360
300
Sorafenib_2 (nM)
10
30
100
300
1000
3000
10000
Motesanib (nM)

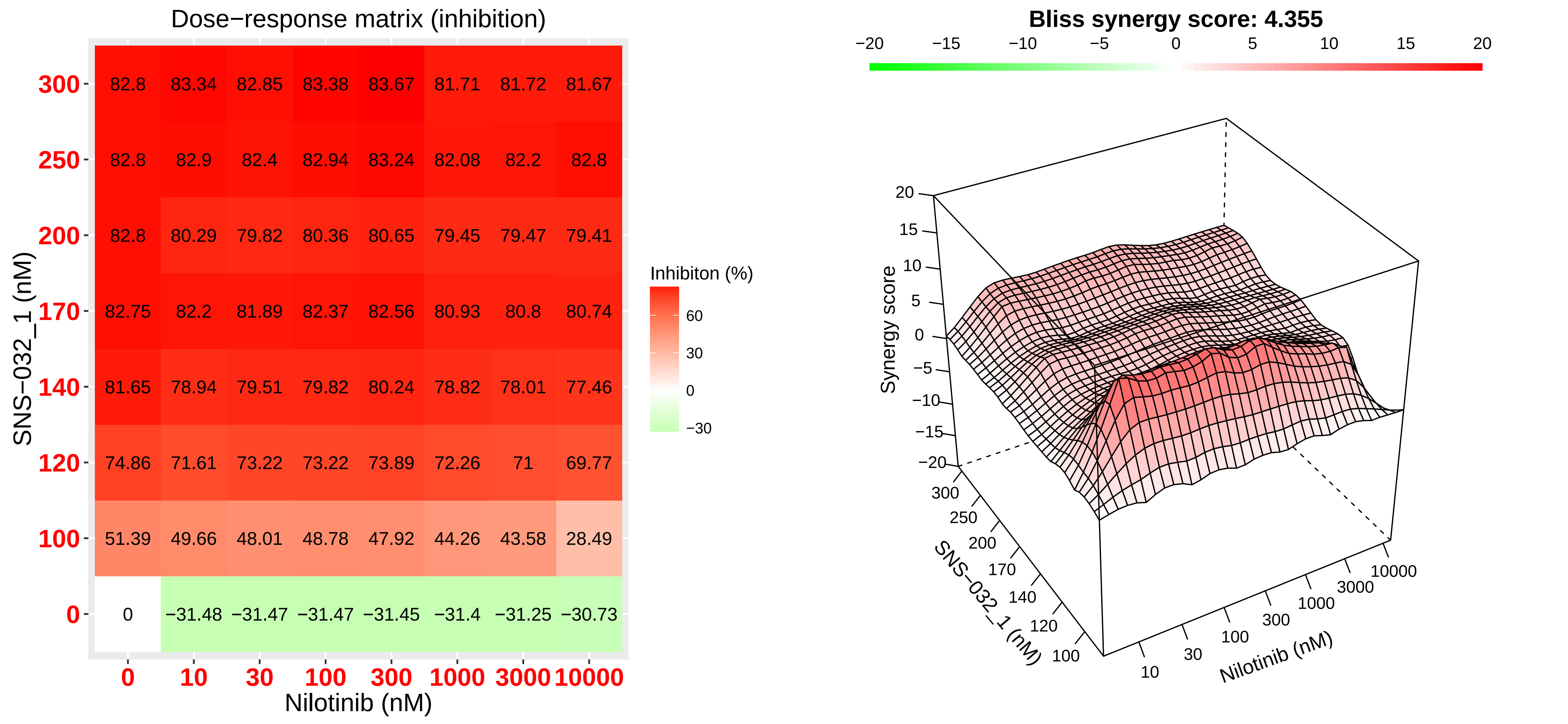
Dose−response matrix (inhibition)
300 82.8 83.34 82.85 83.38 83.67 81.71 81.72 81.67
250 82.8 82.9 82.4 82.94 83.24 82.08 82.2 82.8
200 82.8 80.29 79.82 80.36 80.65 79.45 79.47 79.41
170 82.75 82.2 81.89 82.37 82.56 80.93 80.8 80.74
140 81.65 78.94 79.51 79.82 80.24 78.82 78.01 77.46
120 74.86 71.61 73.22 73.22 73.89 72.26 71 69.77
100 51.39 49.66 48.01 48.78 47.92 44.26 43.58 28.49
0 0 −31.48 −31.47 −31.47 −31.45 −31.4 −31.25 −30.73
0 10 30 100 300 1000 3000 10000
Nilotinib (nM)
SNS−032_1 (nM)
Inhibiton (%)
60
30
0
−30
Bliss synergy score: 4.355
−20 −15 −10 −5 0 5 10 15 20
Synergy score
20 15 10 5 0 −5 −10 −15 −20
SNS−032_1 (nM)
300 250 200 170 140 120 100
Nilotinib (nM)
10 30 100 300 1000 3000 10000

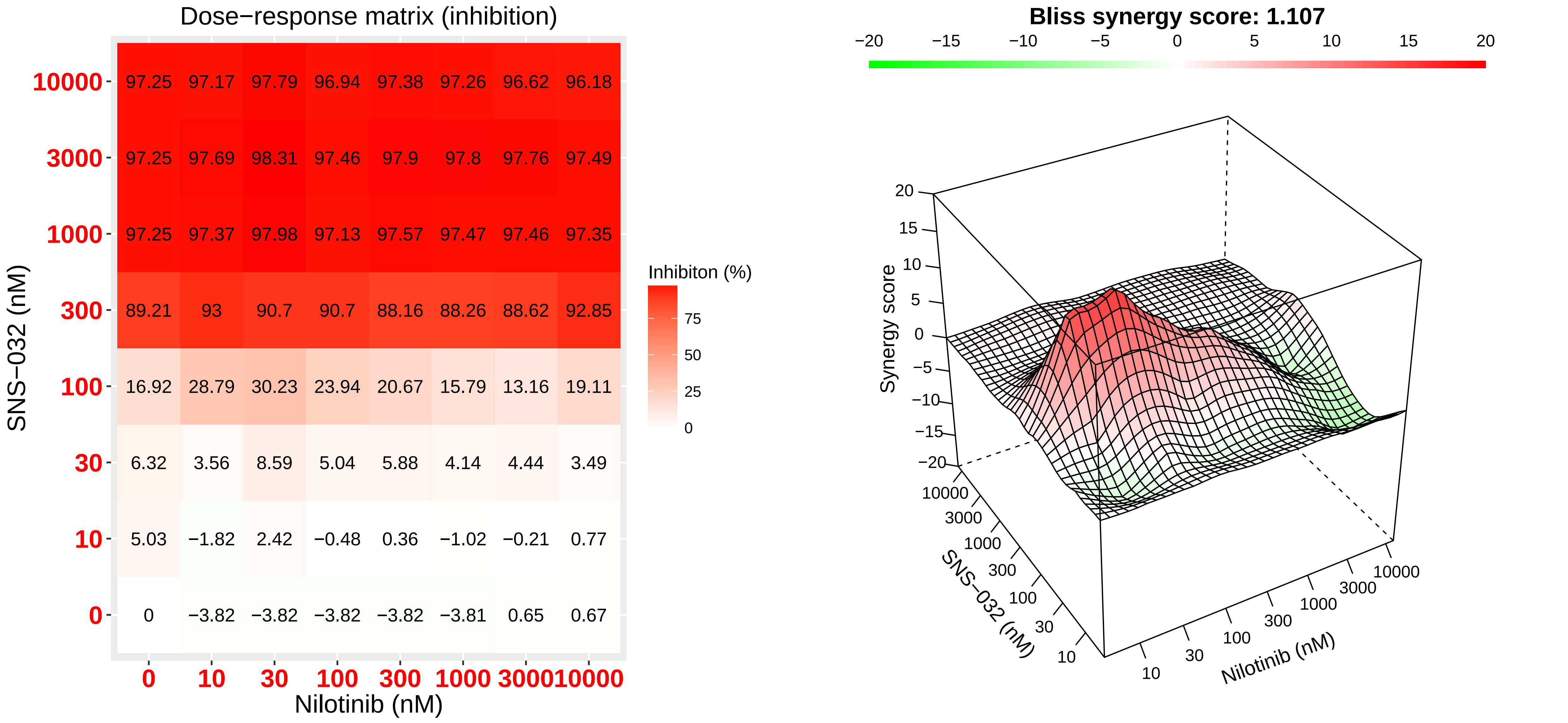

Dose−response matrix (inhibition)
SNS−032 (nM)
Nilotinib (nM)
0 10 30 100 300 1000 3000 10000
10000: 97.25 97.17 97.79 96.94 97.38 97.26 96.62 96.18
3000: 97.25 97.69 98.31 97.46 97.9 97.8 97.76 97.49
1000: 97.25 97.37 97.98 97.13 97.57 97.47 97.46 97.35
300: 89.21 93 90.7 90.7 88.16 88.26 88.62 92.85
100: 16.92 28.79 30.23 23.94 20.67 15.79 13.16 19.11
30: 6.32 3.56 8.59 5.04 5.88 4.14 4.44 3.49
10: 5.03 −1.82 2.42 −0.48 0.36 −1.02 −0.21 0.77
0: 0 −3.82 −3.82 −3.82 −3.82 −3.81 0.65 0.67
Inhibiton (%)
75
50
25
0
Bliss synergy score: 1.107
−20 −15 −10 −5 0 5 10 15 20
Synergy score
20 15 10 5 0 −5 −10 −15 −20
10000 3000 1000 300 100 30 10
SNS−032 (nM)
10 30 100 300 1000 3000 10000
Nilotinib (nM)

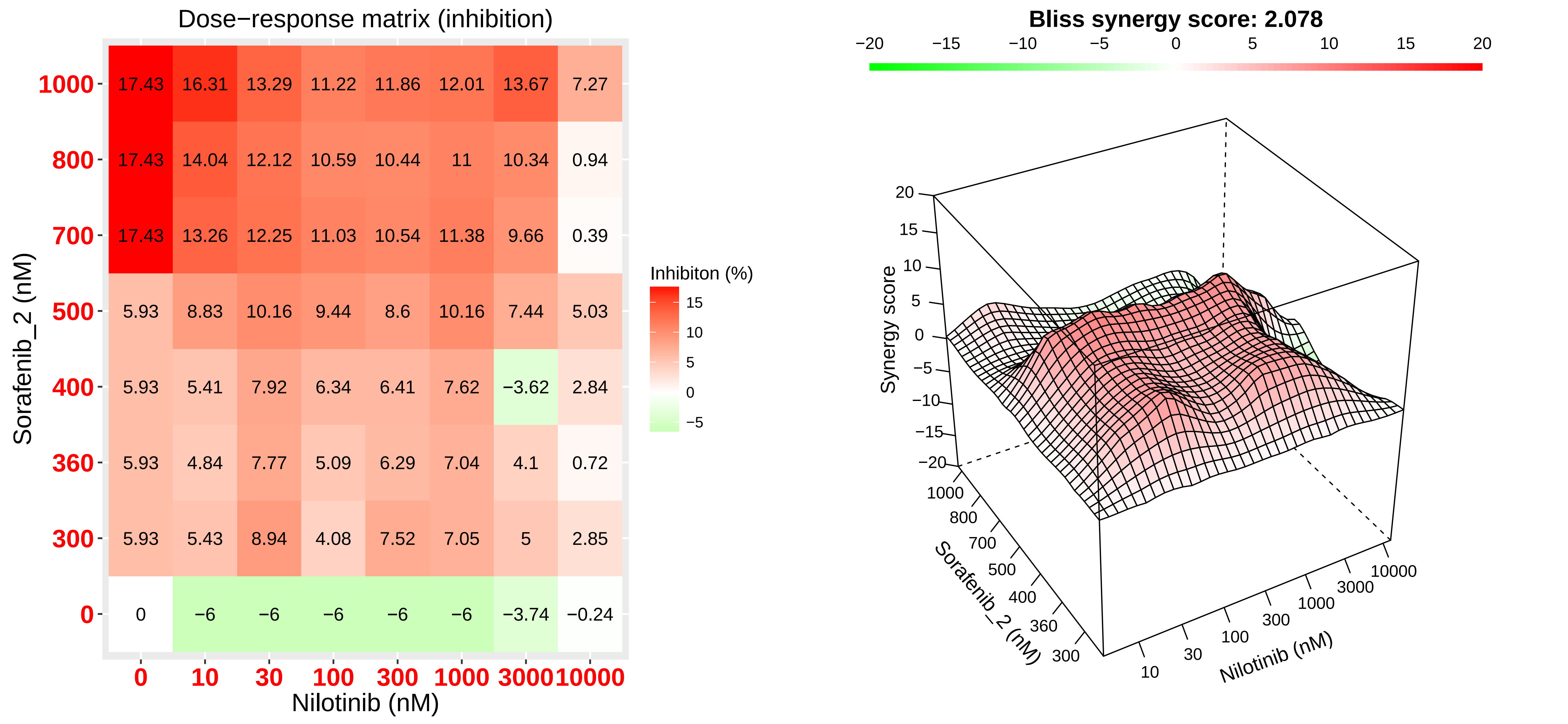
Dose−response matrix (inhibition)
1000
17.43 16.31 13.29 11.22 11.86 12.01 13.67 7.27
800
17.43 14.04 12.12 10.59 10.44 11 10.34 0.94
700
17.43 13.26 12.25 11.03 10.54 11.38 9.66 0.39
500
5.93 8.83 10.16 9.44 8.6 10.16 7.44 5.03
400
5.93 5.41 7.92 6.34 6.41 7.62 −3.62 2.84
360
5.93 4.84 7.77 5.09 6.29 7.04 4.1 0.72
300
5.93 5.43 8.94 4.08 7.52 7.05 5 2.85
0
0 −6 −6 −6 −6 −6 −3.74 −0.24
0 10 30 100 300 1000 3000 10000
Nilotinib (nM)
Sorafenib_2 (nM)
Inhibiton (%)
15
10
5
0
−5
Bliss synergy score: 2.078
−20 −15 −10 −5 0 5 10 15 20
Synergy score
20 15 10 5 0 −5 −10 −15 −20
1000 800 700 500 400 360 300
Sorafenib_2 (nM)
10 30 100 300 1000 3000 10000
Nilotinib (nM)

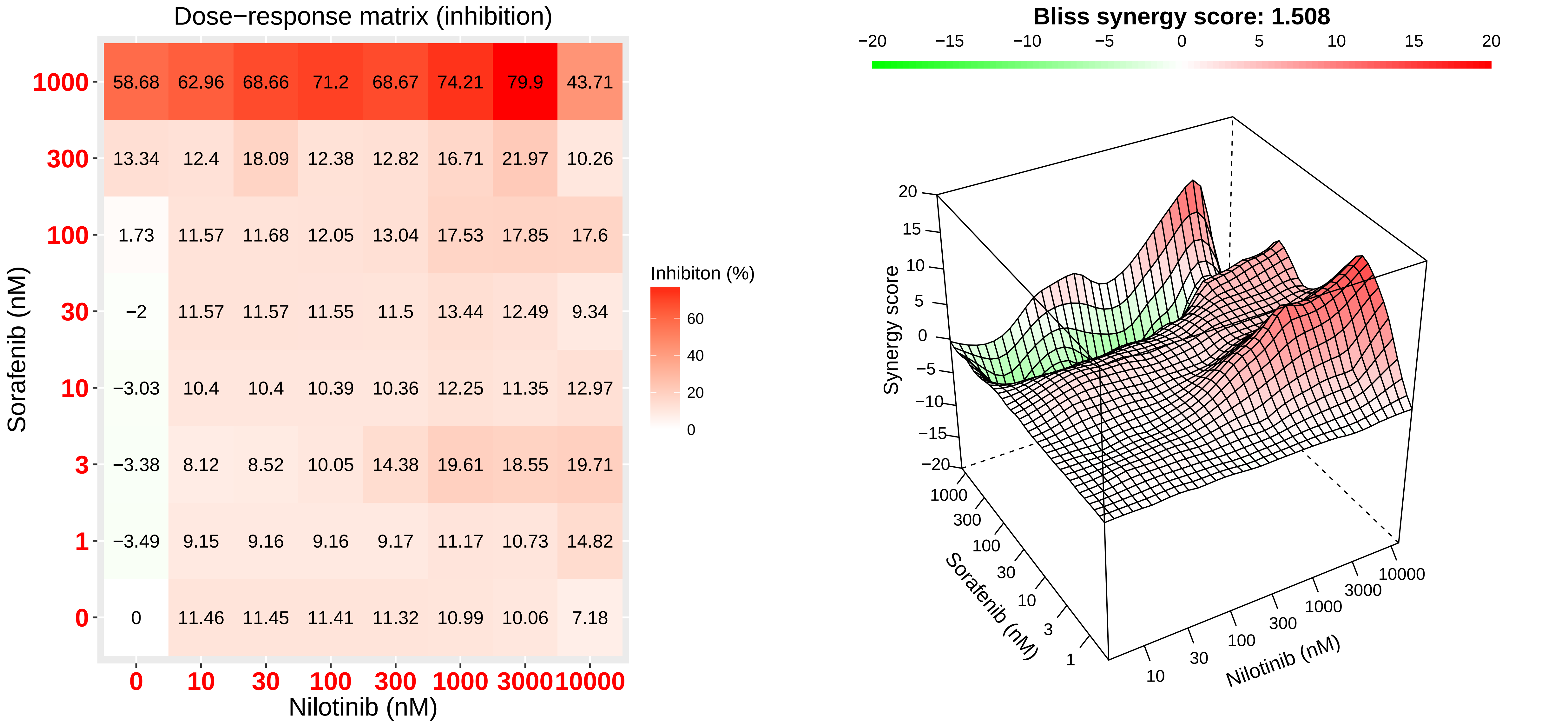
Dose−response matrix (inhibition)
Sorafenib (nM)
Nilotinib (nM)
1000 58.68 62.96 68.66 71.2 68.67 74.21 79.9 43.71
300 13.34 12.4 18.09 12.38 12.82 16.71 21.97 10.26
100 1.73 11.57 11.68 12.05 13.04 17.53 17.85 17.6
30 −2 11.57 11.57 11.55 11.5 13.44 12.49 9.34
10 −3.03 10.4 10.4 10.39 10.36 12.25 11.35 12.97
3 −3.38 8.12 8.52 10.05 14.38 19.61 18.55 19.71
1 −3.49 9.15 9.16 9.16 9.17 11.17 10.73 14.82
0 0 11.46 11.45 11.41 11.32 10.99 10.06 7.18
0 10 30 100 300 1000 3000 10000
Inhibiton (%)
60
40
20
0
Bliss synergy score: 1.508
−20 −15 −10 −5 0 5 10 15 20
Synergy score
20 15 10 5 0 −5 −10 −15 −20
Sorafenib (nM)
1000 300 100 30 10 3 1
Nilotinib (nM)
10 30 100 300 1000 3000 10000

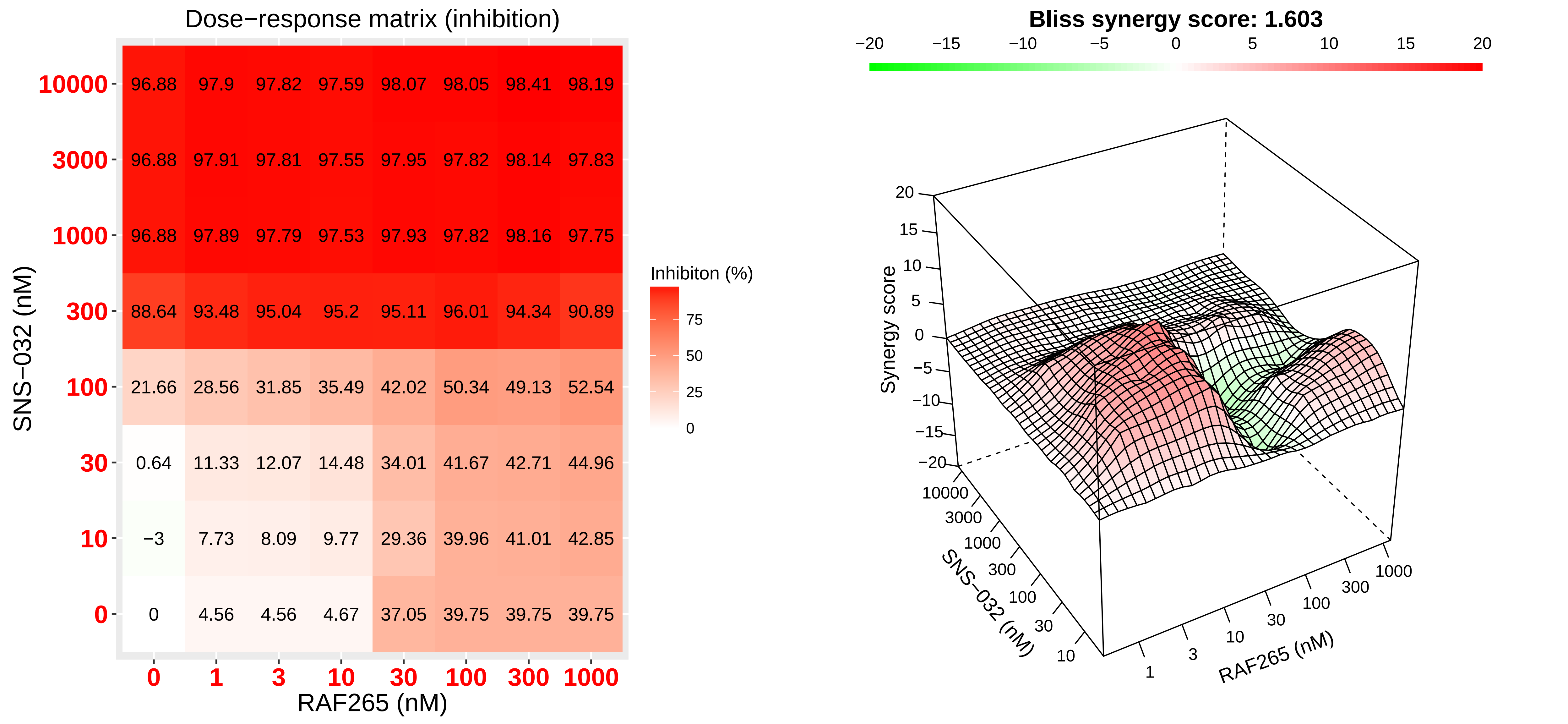
Dose−response matrix (inhibition)
10000 96.88 97.9 97.82 97.59 98.07 98.05 98.41 98.19
3000 96.88 97.91 97.81 97.55 97.95 97.82 98.14 97.83
1000 96.88 97.89 97.79 97.53 97.93 97.82 98.16 97.75
300 88.64 93.48 95.04 95.2 95.11 96.01 94.34 90.89
100 21.66 28.56 31.85 35.49 42.02 50.34 49.13 52.54
30 0.64 11.33 12.07 14.48 34.01 41.67 42.71 44.96
10 −3 7.73 8.09 9.77 29.36 39.96 41.01 42.85
0 0 4.56 4.56 4.67 37.05 39.75 39.75 39.75
0 1 3 10 30 100 300 1000
RAF265 (nM)
SNS−032 (nM)
Inhibiton (%)
75
50
25
0
Bliss synergy score: 1.603
−20 −15 −10 −5 0 5 10 15 20
Synergy score
20
15
10
5
0
−5
−10
−15
−20
10000
3000
1000
300
100
30
10
SNS−032 (nM)
1
3
10
30
100
300
1000
RAF265 (nM)

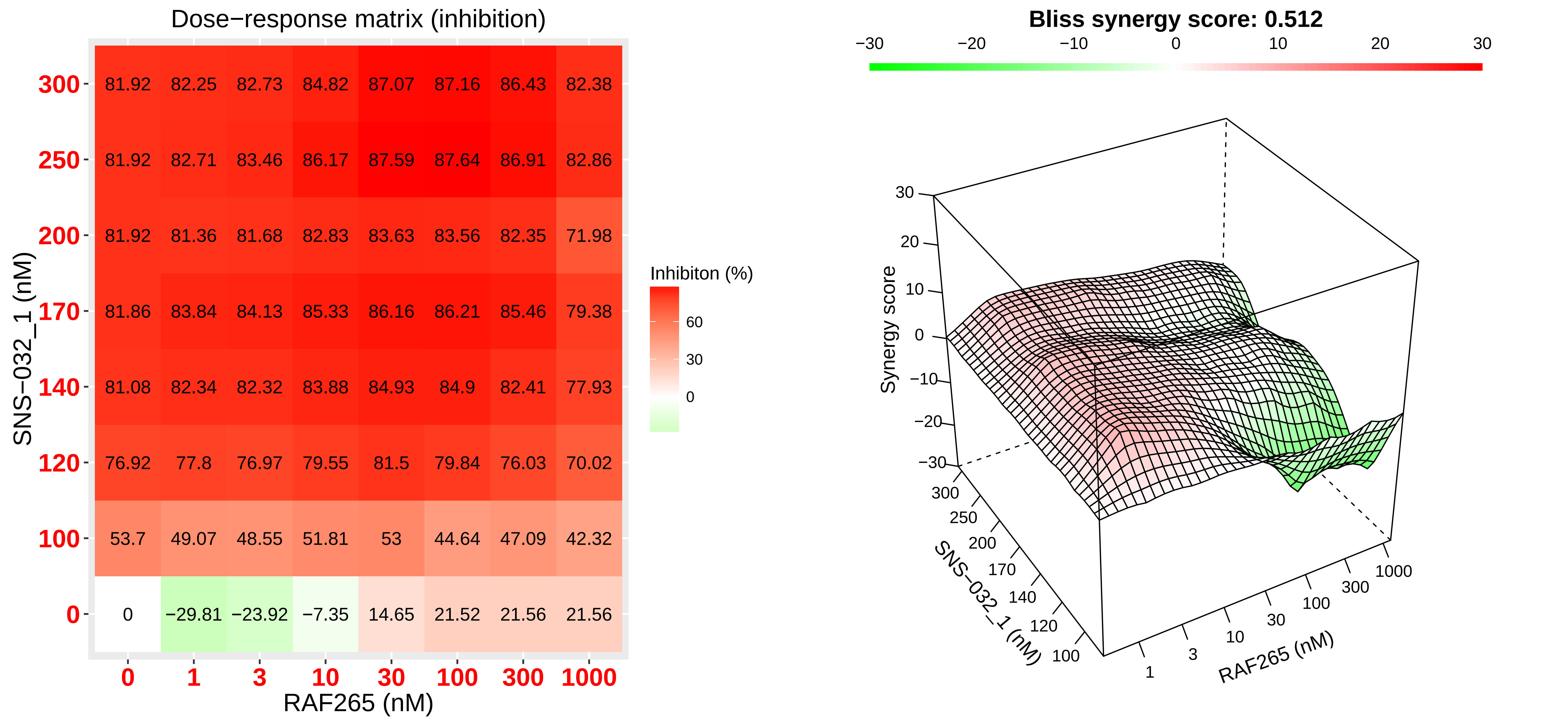

Dose−response matrix (inhibition)
SNS−032_1 (nM)
RAF265 (nM)
300: 81.92 82.25 82.73 84.82 87.07 87.16 86.43 82.38
250: 81.92 82.71 83.46 86.17 87.59 87.64 86.91 82.86
200: 81.92 81.36 81.68 82.83 83.63 83.56 82.35 71.98
170: 81.86 83.84 84.13 85.33 86.16 86.21 85.46 79.38
140: 81.08 82.34 82.32 83.88 84.93 84.9 82.41 77.93
120: 76.92 77.8 76.97 79.55 81.5 79.84 76.03 70.02
100: 53.7 49.07 48.55 51.81 53 44.64 47.09 42.32
0: 0 −29.81 −23.92 −7.35 14.65 21.52 21.56 21.56
0 1 3 10 30 100 300 1000
Inhibiton (%)
60
30
0
Bliss synergy score: 0.512
−30 −20 −10 0 10 20 30
Synergy score
30 20 10 0 −10 −20 −30
SNS−032_1 (nM)
300 250 200 170 140 120 100
RAF265 (nM)
1 3 10 30 100 300 1000

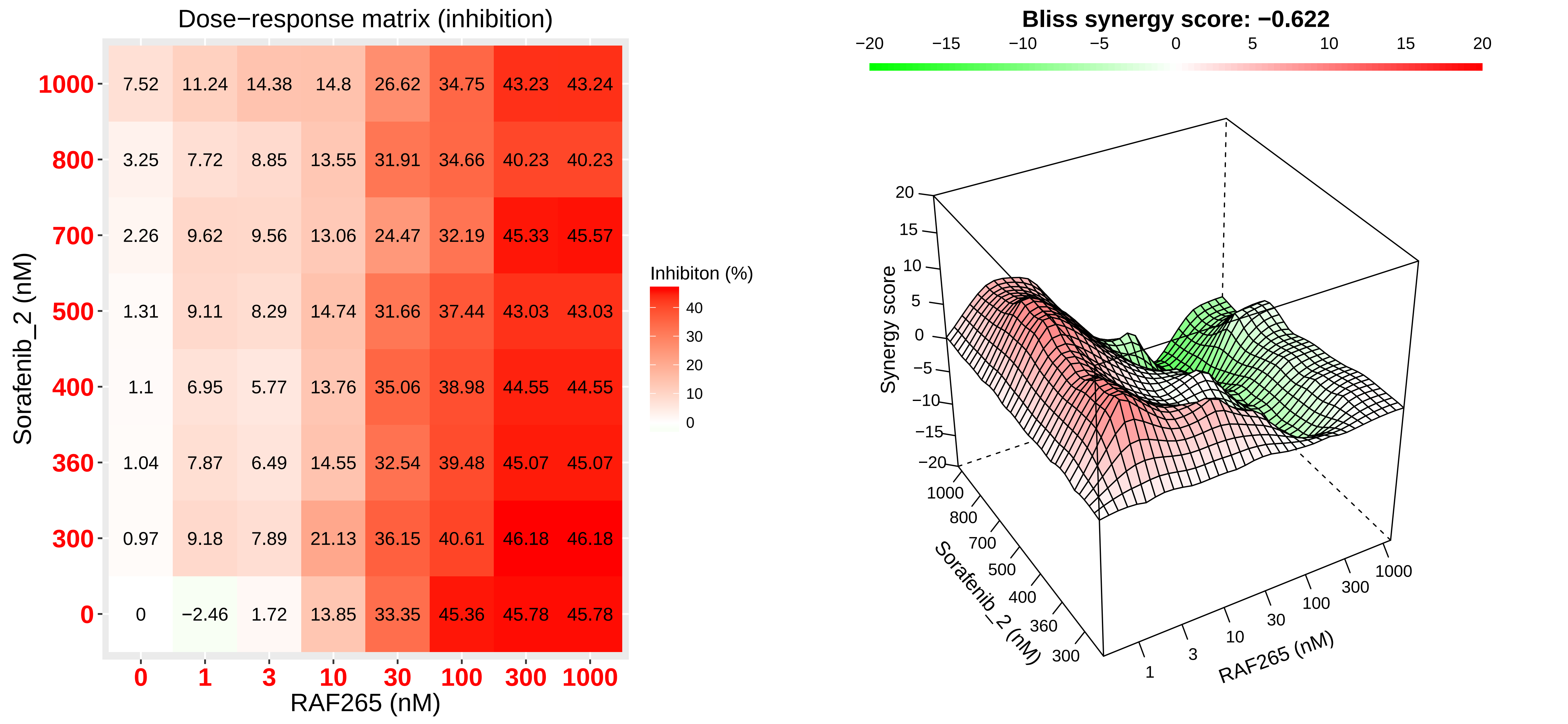
Dose−response matrix (inhibition)
Sorafenib_2 (nM)
RAF265 (nM)
1000 7.52 11.24 14.38 14.8 26.62 34.75 43.23 43.24
800 3.25 7.72 8.85 13.55 31.91 34.66 40.23 40.23
700 2.26 9.62 9.56 13.06 24.47 32.19 45.33 45.57
500 1.31 9.11 8.29 14.74 31.66 37.44 43.03 43.03
400 1.1 6.95 5.77 13.76 35.06 38.98 44.55 44.55
360 1.04 7.87 6.49 14.55 32.54 39.48 45.07 45.07
300 0.97 9.18 7.89 21.13 36.15 40.61 46.18 46.18
0 0 −2.46 1.72 13.85 33.35 45.36 45.78 45.78
0 1 3 10 30 100 300 1000
Inhibiton (%)
40
30
20
10
0
Bliss synergy score: −0.622
−20 −15 −10 −5 0 5 10 15 20
Synergy score
20 15 10 5 0 −5 −10 −15 −20
Sorafenib_2 (nM)
1000 800 700 500 400 360 300
RAF265 (nM)
1 3 10 30 100 300 1000

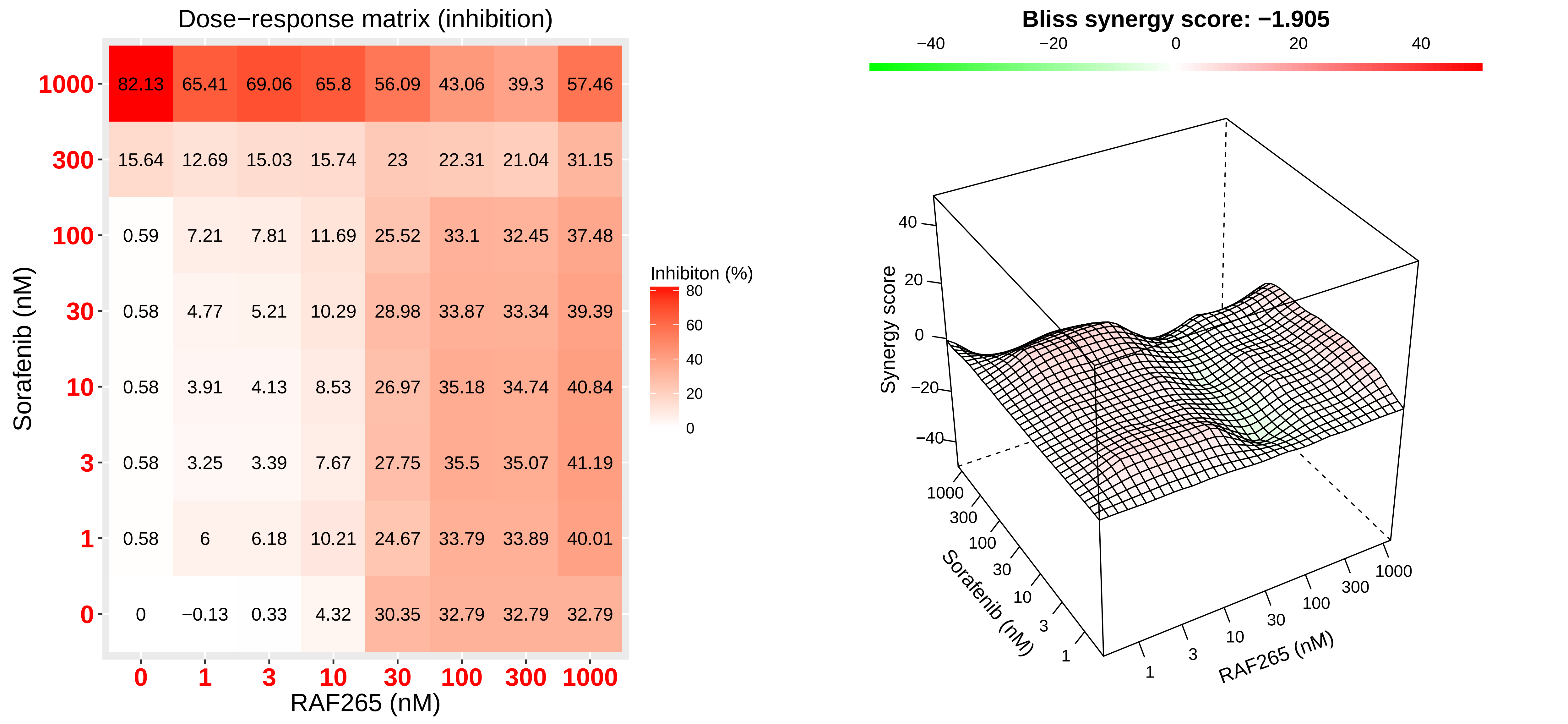

Dose−response matrix (inhibition)
1000
82.13 65.41 69.06 65.8 56.09 43.06 39.3 57.46
300
15.64 12.69 15.03 15.74 23 22.31 21.04 31.15
100
0.59 7.21 7.81 11.69 25.52 33.1 32.45 37.48
30
0.58 4.77 5.21 10.29 28.98 33.87 33.34 39.39
10
0.58 3.91 4.13 8.53 26.97 35.18 34.74 40.84
3
0.58 3.25 3.39 7.67 27.75 35.5 35.07 41.19
1
0.58 6 6.18 10.21 24.67 33.79 33.89 40.01
0
0 −0.13 0.33 4.32 30.35 32.79 32.79 32.79
0 1 3 10 30 100 300 1000
RAF265 (nM)
Sorafenib (nM)
Inhibiton (%)
80
60
40
20
0
Bliss synergy score: −1.905
−40 −20 0 20 40
Synergy score
40
20
0
−20
−40
Sorafenib (nM)
1000 300 100 30 10 3 1
RAF265 (nM)
1 3 10 30 100 300 1000

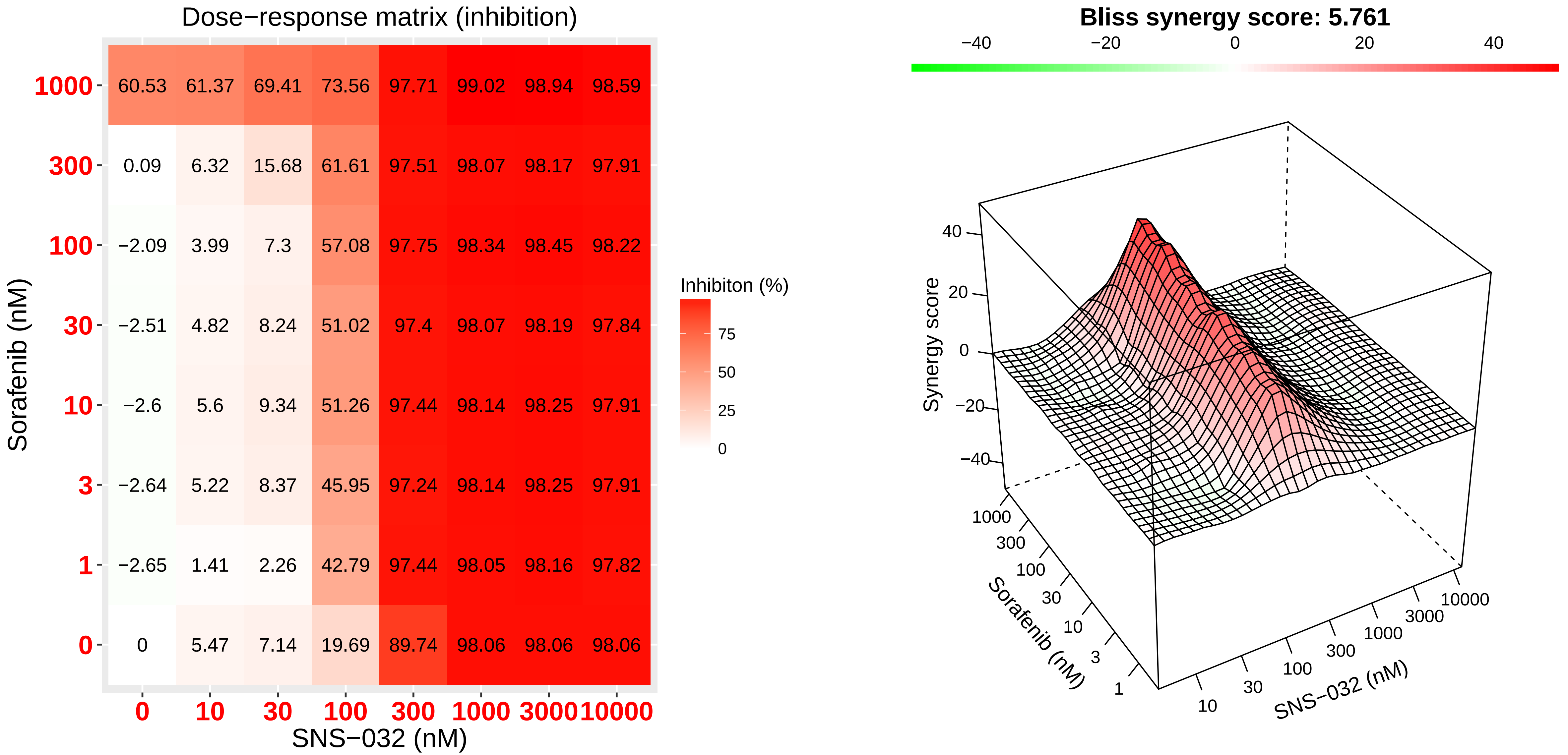
Dose−response matrix (inhibition)
1000
60.53 61.37 69.41 73.56 97.71 99.02 98.94 98.59
300
0.09 6.32 15.68 61.61 97.51 98.07 98.17 97.91
100
−2.09 3.99 7.3 57.08 97.75 98.34 98.45 98.22
30
−2.51 4.82 8.24 51.02 97.4 98.07 98.19 97.84
10
−2.6 5.6 9.34 51.26 97.44 98.14 98.25 97.91
3
−2.64 5.22 8.37 45.95 97.24 98.14 98.25 97.91
1
−2.65 1.41 2.26 42.79 97.44 98.05 98.16 97.82
0
0 5.47 7.14 19.69 89.74 98.06 98.06 98.06
0 10 30 100 300 1000 3000 10000
SNS−032 (nM)
Sorafenib (nM)
Inhibiton (%)
75
50
25
0
Bliss synergy score: 5.761
−40 −20 0 20 40
Synergy score
40
20
0
−20
−40
1000
300
100
30
10
3
1
Sorafenib (nM)
10 30 100 300 1000 3000 10000
SNS−032 (nM)

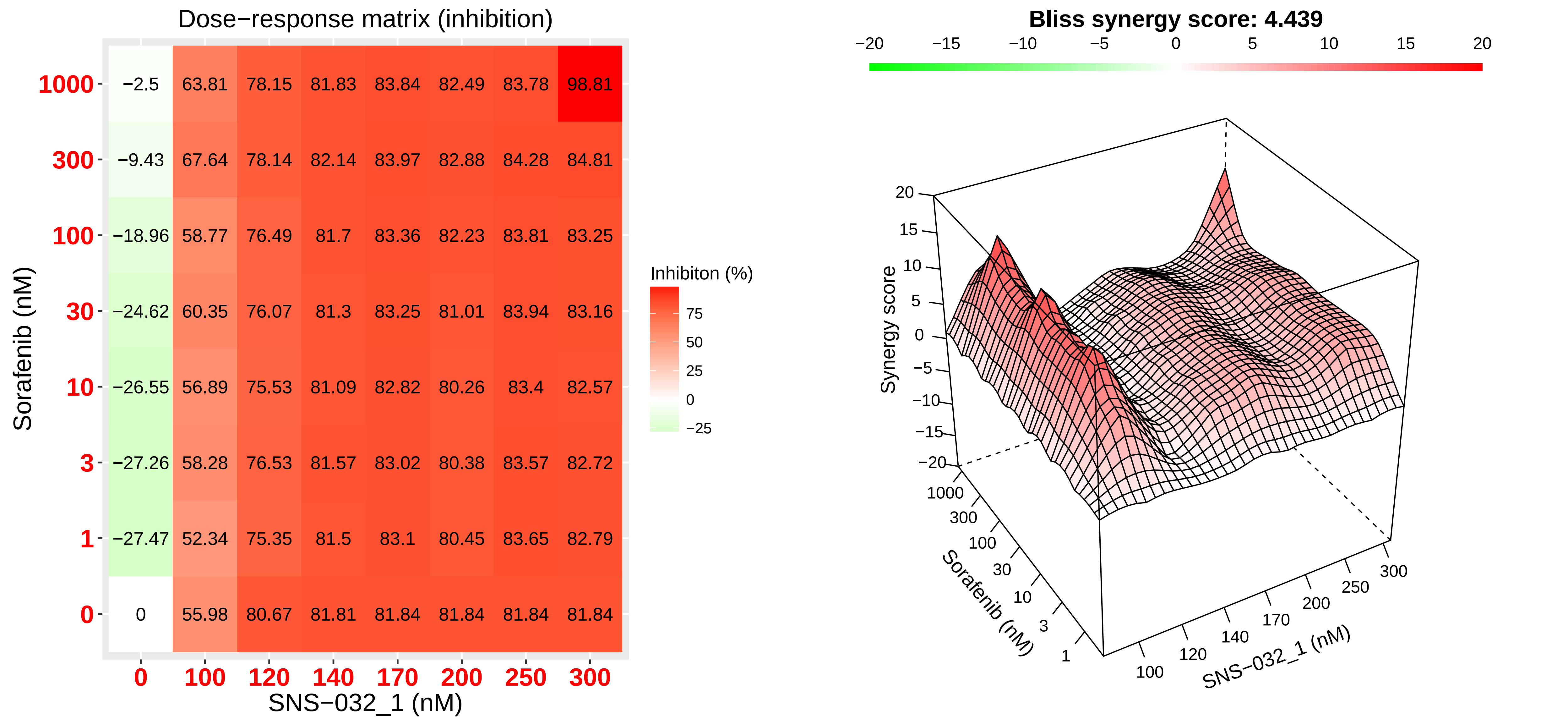
Dose−response matrix (inhibition)
1000 −2.5 63.81 78.15 81.83 83.84 82.49 83.78 98.81
300 −9.43 67.64 78.14 82.14 83.97 82.88 84.28 84.81
100 −18.96 58.77 76.49 81.7 83.36 82.23 83.81 83.25
30 −24.62 60.35 76.07 81.3 83.25 81.01 83.94 83.16
10 −26.55 56.89 75.53 81.09 82.82 80.26 83.4 82.57
3 −27.26 58.28 76.53 81.57 83.02 80.38 83.57 82.72
1 −27.47 52.34 75.35 81.5 83.1 80.45 83.65 82.79
0 0 55.98 80.67 81.81 81.84 81.84 81.84 81.84
0 100 120 140 170 200 250 300
SNS−032_1 (nM)
Sorafenib (nM)
Inhibiton (%)
75
50
25
0
−25
Bliss synergy score: 4.439
−20 −15 −10 −5 0 5 10 15 20
Synergy score
20 15 10 5 0 −5 −10 −15 −20
1000 300 100 30 10 3 1
Sorafenib (nM)
100 120 140 170 200 250 300
SNS−032_1 (nM)

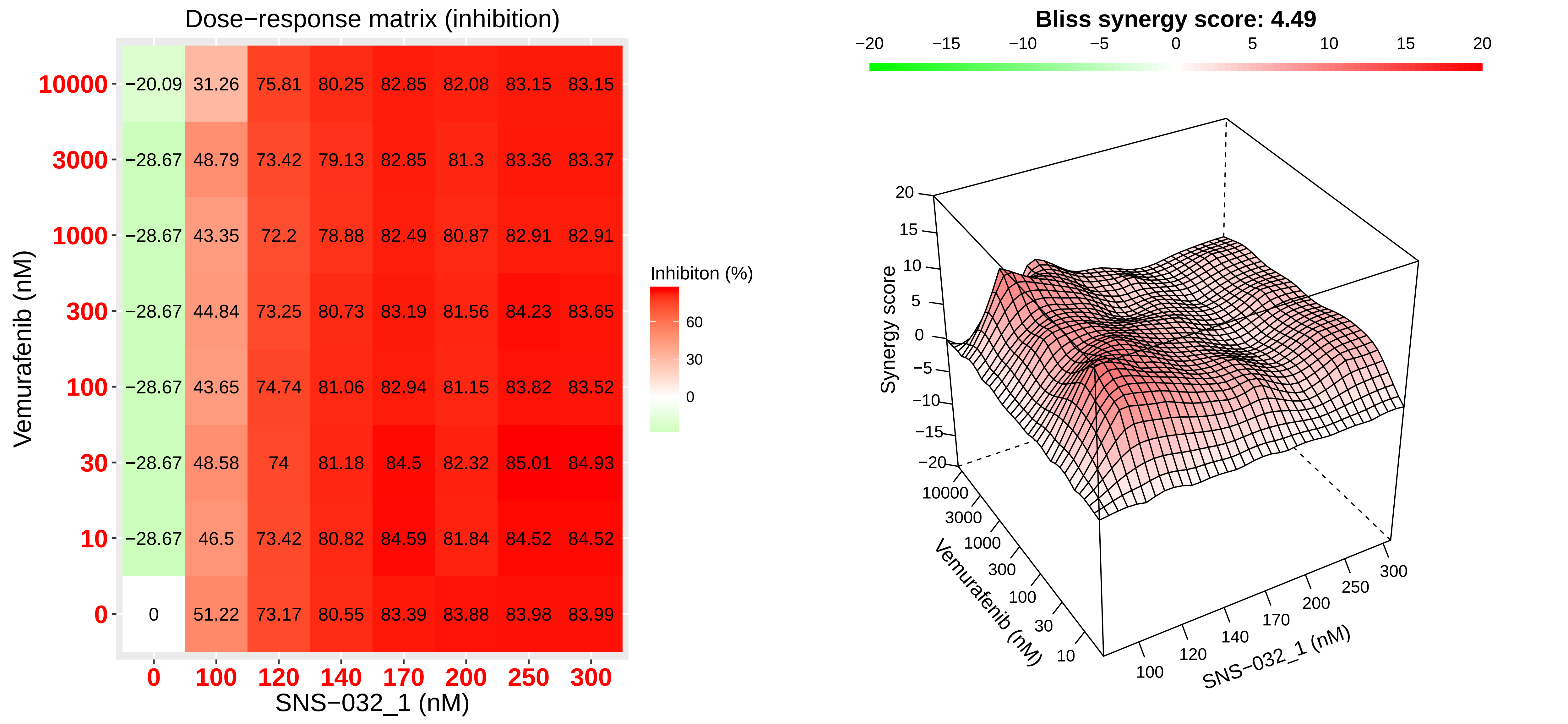

Dose−response matrix (inhibition)
Vemurafenib (nM)
10000 −20.09 31.26 75.81 80.25 82.85 82.08 83.15 83.15
3000 −28.67 48.79 73.42 79.13 82.85 81.3 83.36 83.37
1000 −28.67 43.35 72.2 78.88 82.49 80.87 82.91 82.91
300 −28.67 44.84 73.25 80.73 83.19 81.56 84.23 83.65
100 −28.67 43.65 74.74 81.06 82.94 81.15 83.82 83.52
30 −28.67 48.58 74 81.18 84.5 82.32 85.01 84.93
10 −28.67 46.5 73.42 80.82 84.59 81.84 84.52 84.52
0 0 51.22 73.17 80.55 83.39 83.88 83.98 83.99
0 100 120 140 170 200 250 300
SNS−032_1 (nM)
Inhibiton (%)
60
30
0
Bliss synergy score: 4.49
−20 −15 −10 −5 0 5 10 15 20
Synergy score
20 15 10 5 0 −5 −10 −15 −20
Vemurafenib (nM)
10000 3000 1000 300 100 30 10
SNS−032_1 (nM)
100 120 140 170 200 250 300

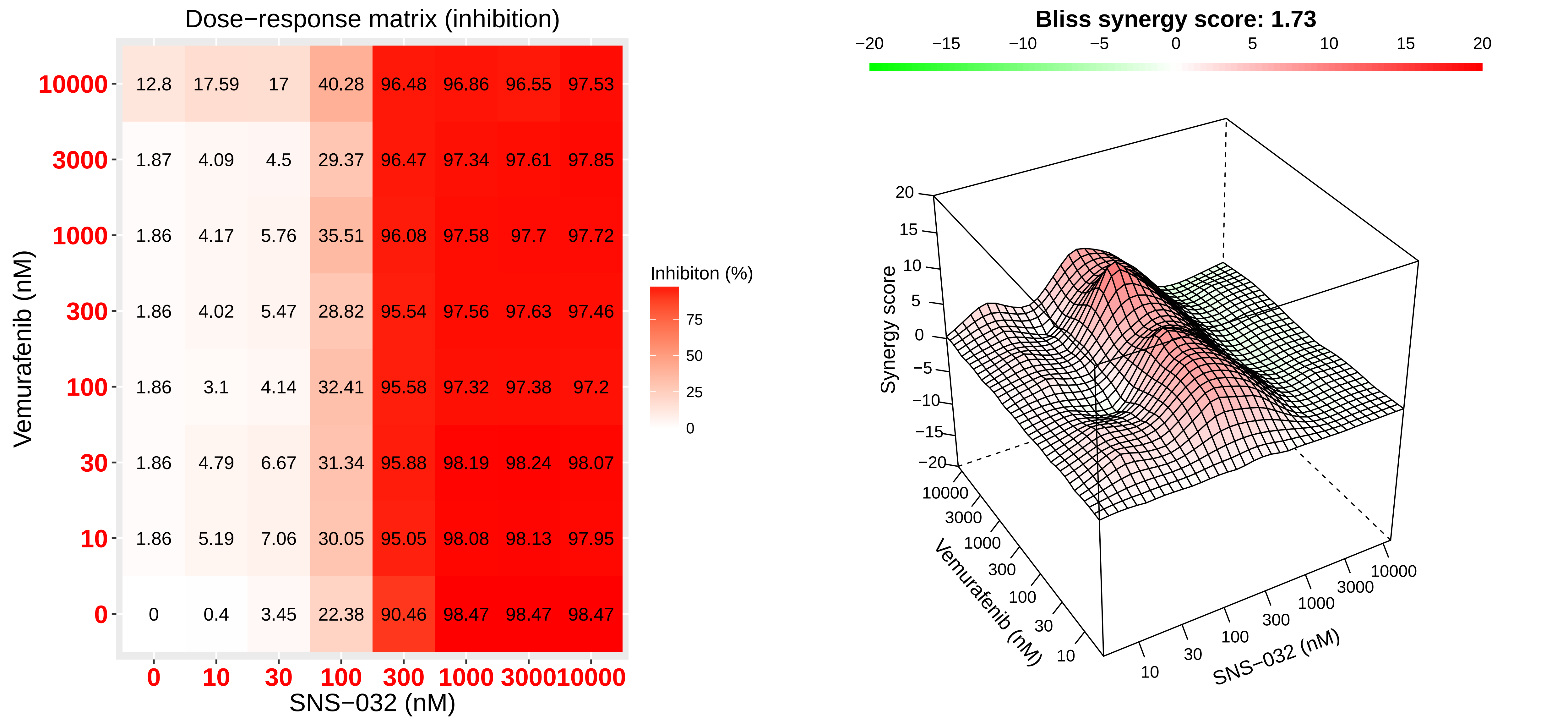
Dose−response matrix (inhibition)
10000 12.8 17.59 17 40.28 96.48 96.86 96.55 97.53
3000 1.87 4.09 4.5 29.37 96.47 97.34 97.61 97.85
1000 1.86 4.17 5.76 35.51 96.08 97.58 97.7 97.72
300 1.86 4.02 5.47 28.82 95.54 97.56 97.63 97.46
100 1.86 3.1 4.14 32.41 95.58 97.32 97.38 97.2
30 1.86 4.79 6.67 31.34 95.88 98.19 98.24 98.07
10 1.86 5.19 7.06 30.05 95.05 98.08 98.13 97.95
0 0 0.4 3.45 22.38 90.46 98.47 98.47 98.47
0 10 30 100 300 1000 3000 10000
SNS−032 (nM)
Vemurafenib (nM)
Inhibiton (%)
75
50
25
0
Bliss synergy score: 1.73
−20 −15 −10 −5 0 5 10 15 20
Synergy score
20 15 10 5 0 −5 −10 −15 −20
Vemurafenib (nM)
10000 3000 1000 300 100 30 10
SNS−032 (nM)
10 30 100 300 1000 3000 10000

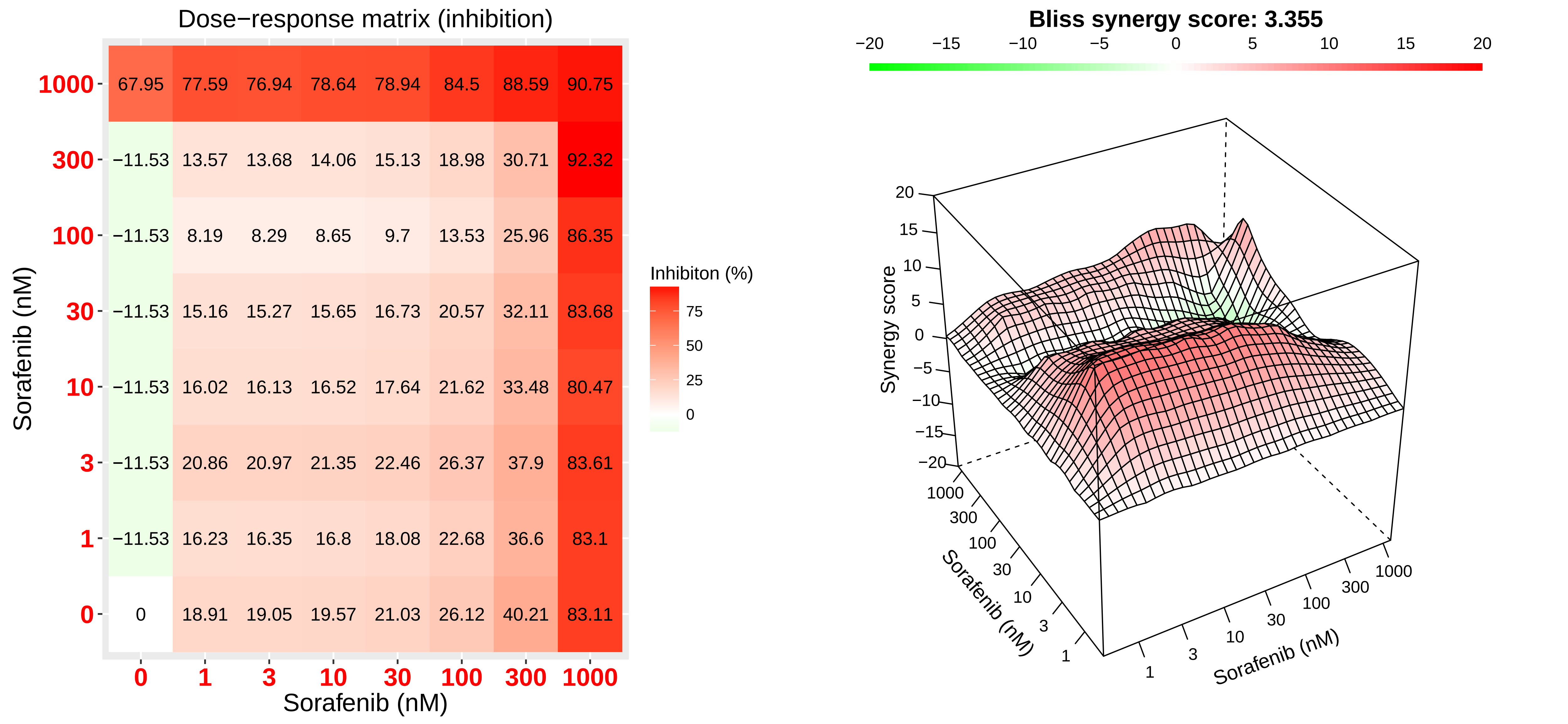
Dose−response matrix (inhibition)
1000
300
100
30
10
3
1
0
Sorafenib (nM)
67.95 77.59 76.94 78.64 78.94 84.5 88.59 90.75
−11.53 13.57 13.68 14.06 15.13 18.98 30.71 92.32
−11.53 8.19 8.29 8.65 9.7 13.53 25.96 86.35
−11.53 15.16 15.27 15.65 16.73 20.57 32.11 83.68
−11.53 16.02 16.13 16.52 17.64 21.62 33.48 80.47
−11.53 20.86 20.97 21.35 22.46 26.37 37.9 83.61
−11.53 16.23 16.35 16.8 18.08 22.68 36.6 83.1
0 18.91 19.05 19.57 21.03 26.12 40.21 83.11
0 1 3 10 30 100 300 1000
Sorafenib (nM)
Inhibiton (%)
75
50
25
0
Bliss synergy score: 3.355
−20 −15 −10 −5 0 5 10 15 20
Synergy score
20
15
10
5
0
−5
−10
−15
−20
1000
300
100
30
10
3
1
Sorafenib (nM)
1
3
10
30
100
300
1000
Sorafenib (nM)

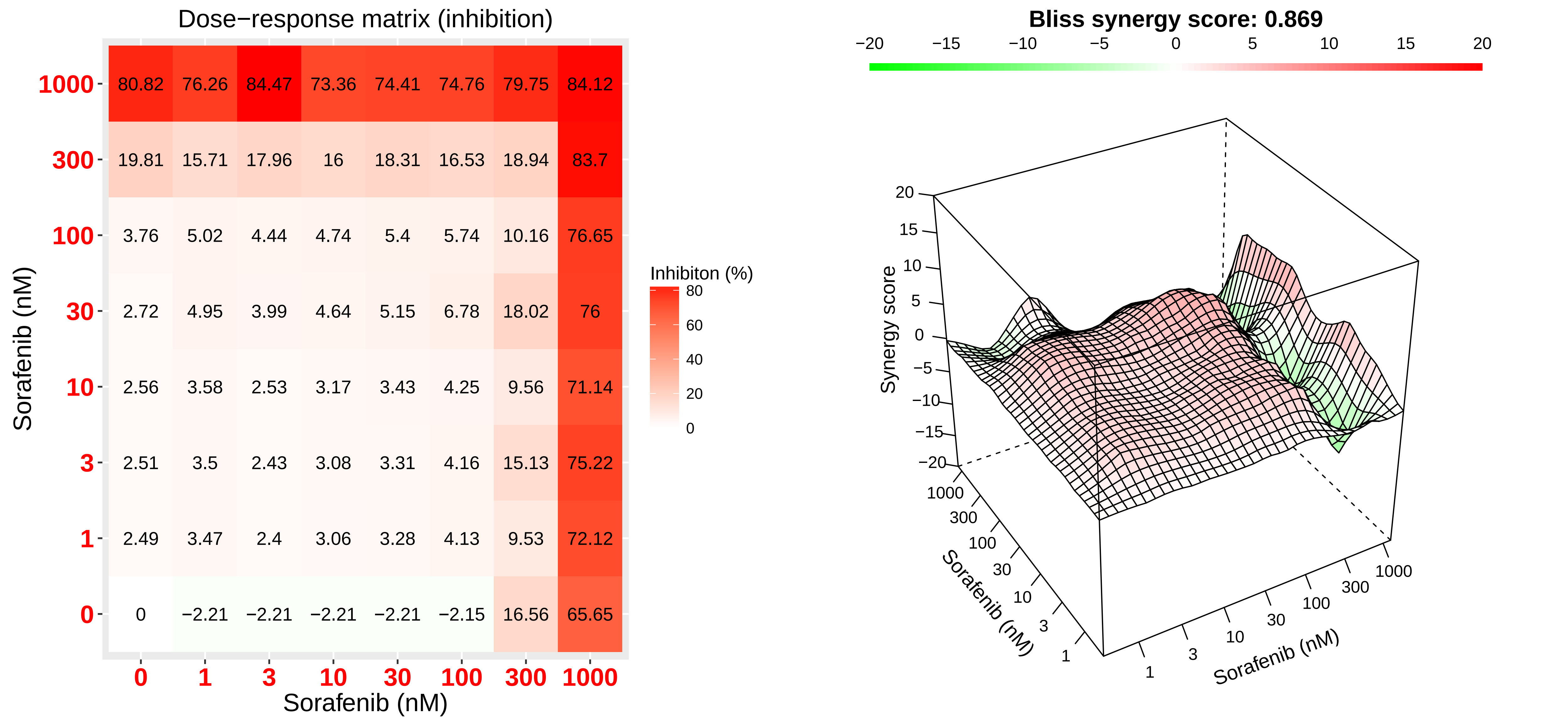
Dose−response matrix (inhibition)
1000 80.82 76.26 84.47 73.36 74.41 74.76 79.75 84.12
300 19.81 15.71 17.96 16 18.31 16.53 18.94 83.7
100 3.76 5.02 4.44 4.74 5.4 5.74 10.16 76.65
30 2.72 4.95 3.99 4.64 5.15 6.78 18.02 76
10 2.56 3.58 2.53 3.17 3.43 4.25 9.56 71.14
3 2.51 3.5 2.43 3.08 3.31 4.16 15.13 75.22
1 2.49 3.47 2.4 3.06 3.28 4.13 9.53 72.12
0 0 −2.21 −2.21 −2.21 −2.21 −2.15 16.56 65.65
0 1 3 10 30 100 300 1000
Sorafenib (nM)
Sorafenib (nM)
Inhibiton (%)
80
60
40
20
0
Bliss synergy score: 0.869
−20 −15 −10 −5 0 5 10 15 20
Synergy score
20 15 10 5 0 −5 −10 −15 −20
1000 300 100 30 10 3 1
Sorafenib (nM)
1 3 10 30 100 300 1000
Sorafenib (nM)

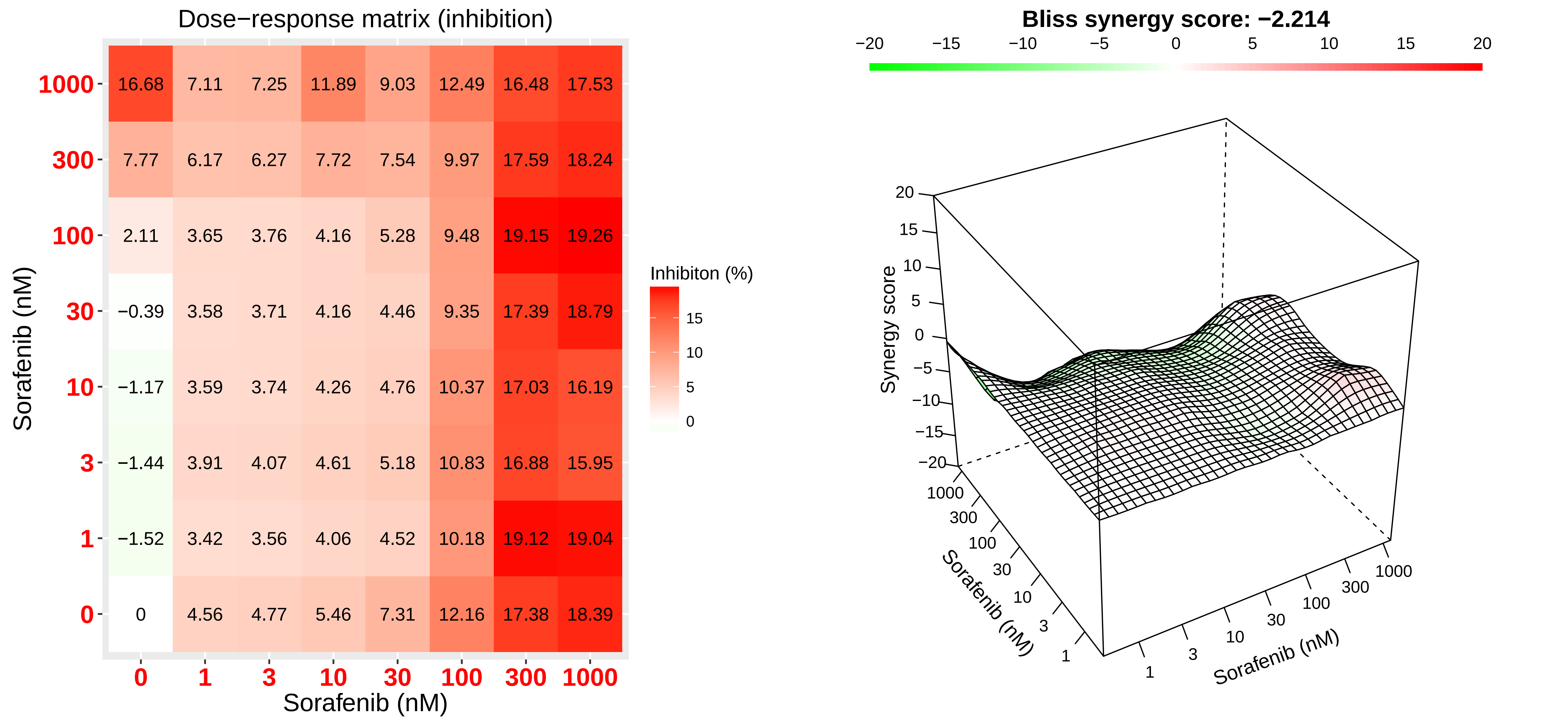

Dose−response matrix (inhibition)
1000 16.68 7.11 7.25 11.89 9.03 12.49 16.48 17.53
300 7.77 6.17 6.27 7.72 7.54 9.97 17.59 18.24
100 2.11 3.65 3.76 4.16 5.28 9.48 19.15 19.26
30 −0.39 3.58 3.71 4.16 4.46 9.35 17.39 18.79
10 −1.17 3.59 3.74 4.26 4.76 10.37 17.03 16.19
3 −1.44 3.91 4.07 4.61 5.18 10.83 16.88 15.95
1 −1.52 3.42 3.56 4.06 4.52 10.18 19.12 19.04
0 0 4.56 4.77 5.46 7.31 12.16 17.38 18.39
0 1 3 10 30 100 300 1000
Sorafenib (nM)
Sorafenib (nM)
Inhibiton (%)
15
10
5
0
Bliss synergy score: −2.214
−20 −15 −10 −5 0 5 10 15 20
Synergy score
20
15
10
5
0
−5
−10
−15
−20
1000
300
100
30
10
3
1
Sorafenib (nM)
1
3
10
30
100
300
1000
Sorafenib (nM)

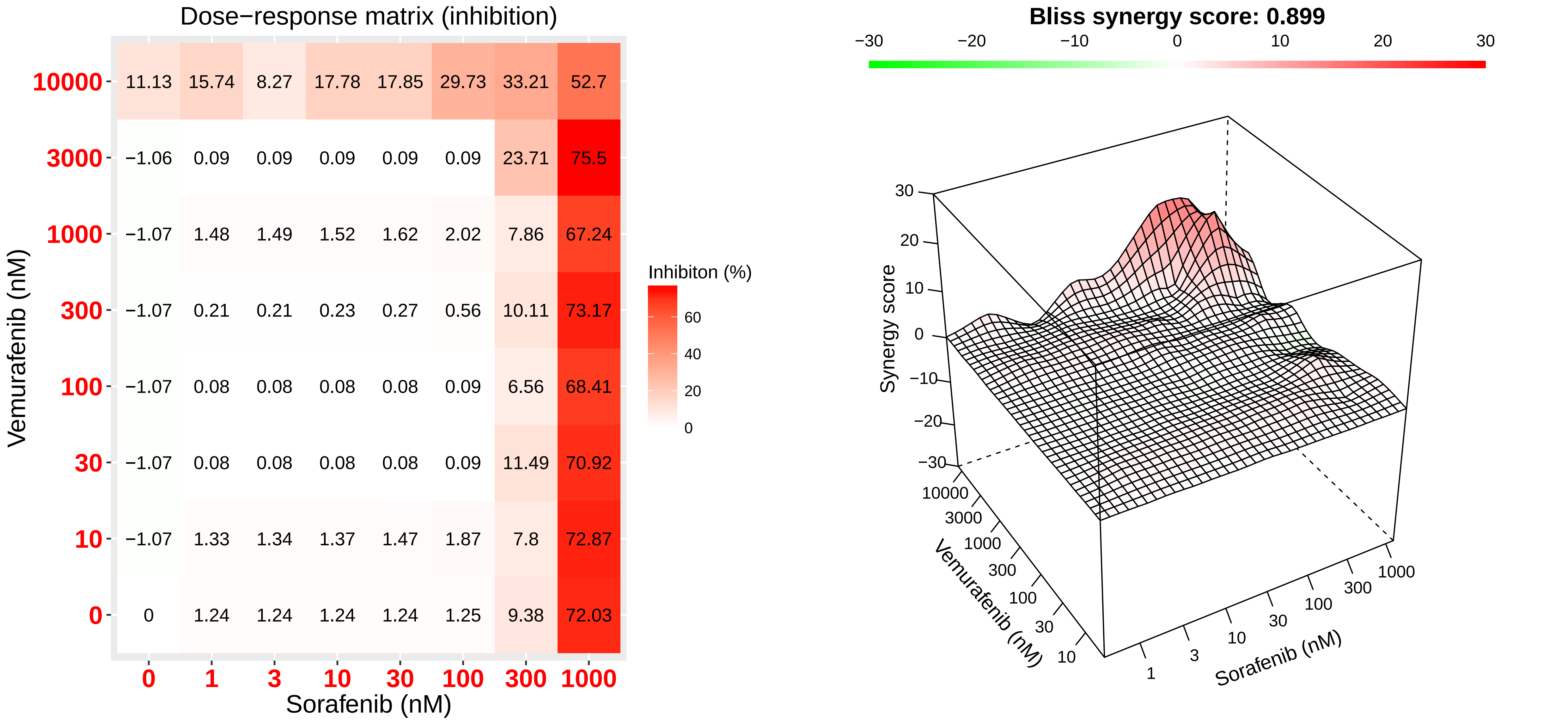

Dose−response matrix (inhibition)
10000
11.13
15.74
8.27
17.78
17.85
29.73
33.21
52.7
3000
−1.06
0.09
0.09
0.09
0.09
0.09
23.71
75.5
1000
−1.07
1.48
1.49
1.52
1.62
2.02
7.86
67.24
300
−1.07
0.21
0.21
0.23
0.27
0.56
10.11
73.17
100
−1.07
0.08
0.08
0.08
0.08
0.09
6.56
68.41
30
−1.07
0.08
0.08
0.08
0.08
0.09
11.49
70.92
10
−1.07
1.33
1.34
1.37
1.47
1.87
7.8
72.87
0
0
1.24
1.24
1.24
1.24
1.25
9.38
72.03
0
1
3
10
30
100
300
1000
Sorafenib (nM)
Vemurafenib (nM)
Inhibiton (%)
60
40
20
0
Bliss synergy score: 0.899
−30
−20
−10
0
10
20
30
Synergy score
30
20
10
0
−10
−20
−30
10000
3000
1000
300
100
30
10
Vemurafenib (nM)
1
3
10
30
100
300
1000
Sorafenib (nM)

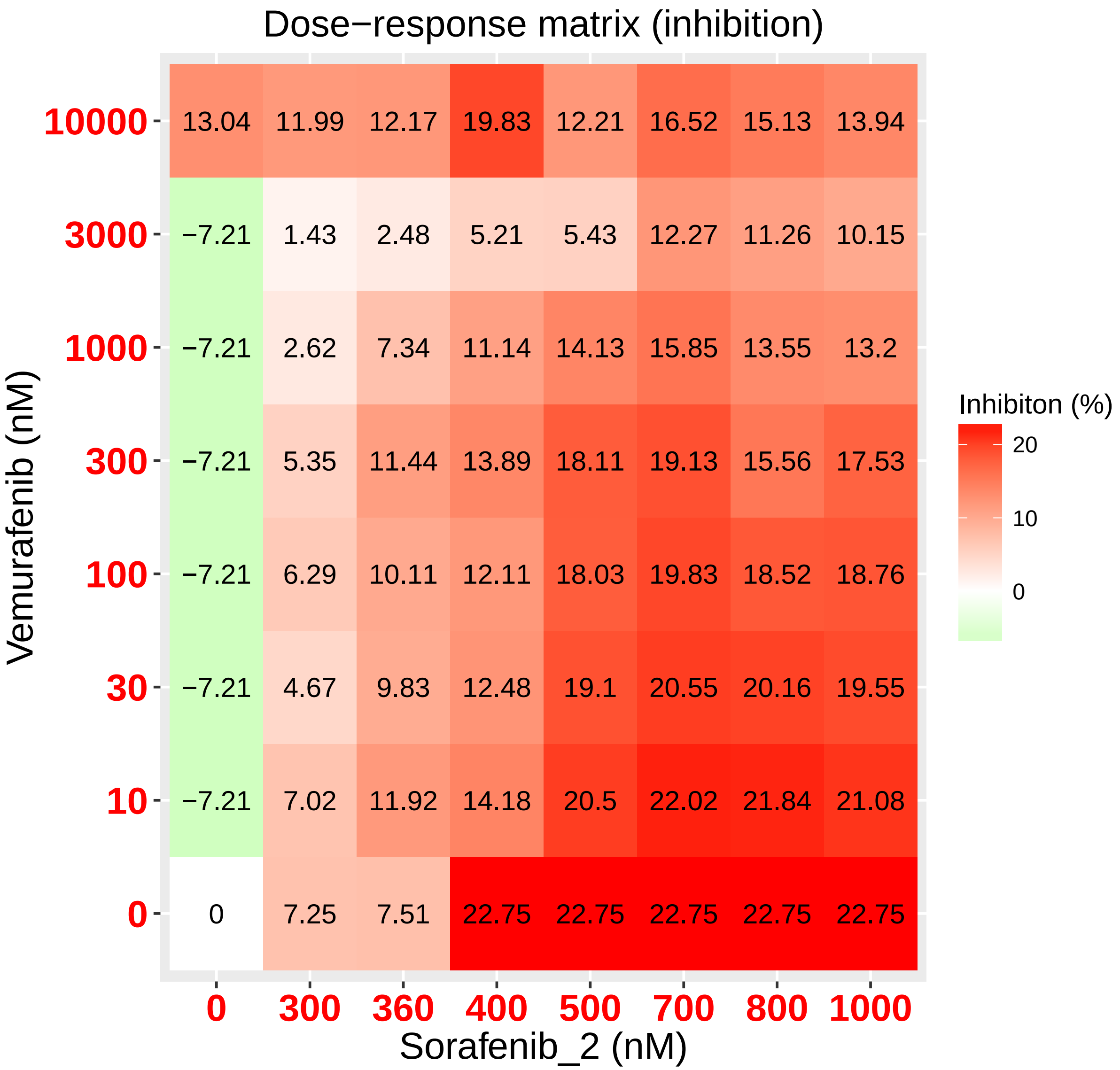
Dose−response matrix (inhibition)
Vemurafenib (nM)
10000
3000
1000
300
100
30
10
0
13.04 11.99 12.17 19.83 12.21 16.52 15.13 13.94
−7.21 1.43 2.48 5.21 5.43 12.27 11.26 10.15
−7.21 2.62 7.34 11.14 14.13 15.85 13.55 13.2
−7.21 5.35 11.44 13.89 18.11 19.13 15.56 17.53
−7.21 6.29 10.11 12.11 18.03 19.83 18.52 18.76
−7.21 4.67 9.83 12.48 19.1 20.55 20.16 19.55
−7.21 7.02 11.92 14.18 20.5 22.02 21.84 21.08
0 7.25 7.51 22.75 22.75 22.75 22.75 22.75
0 300 360 400 500 700 800 1000
Sorafenib_2 (nM)
Inhibiton (%)
20
10
0
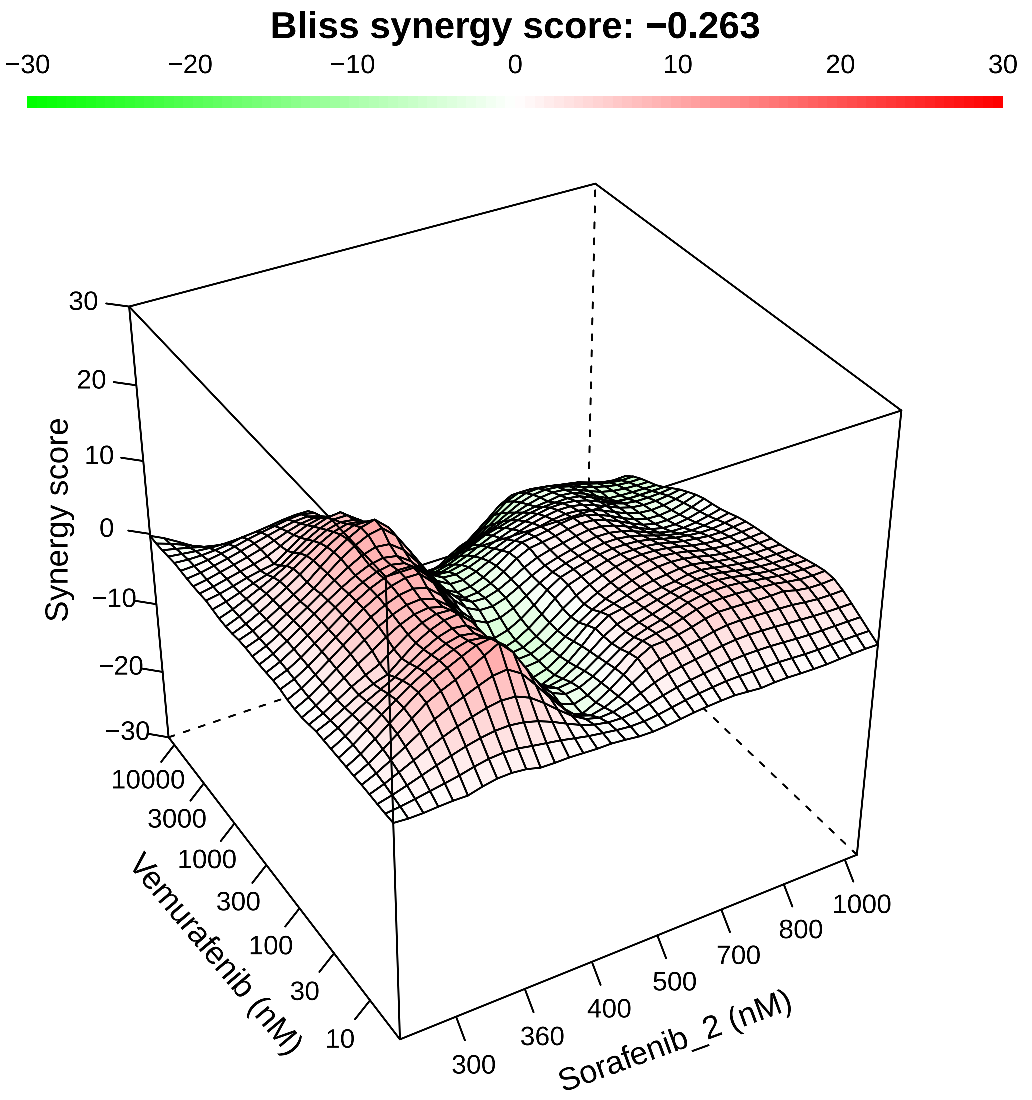
Bliss synergy score: −0.263
−30 −20 −10 0 10 20 30
Synergy score
30
20
10
0
−10
−20
−30
10000
3000
1000
300
100
30
10
Vemurafenib (nM)
300
360
400
500
700
800
1000
Sorafenib_2 (nM)

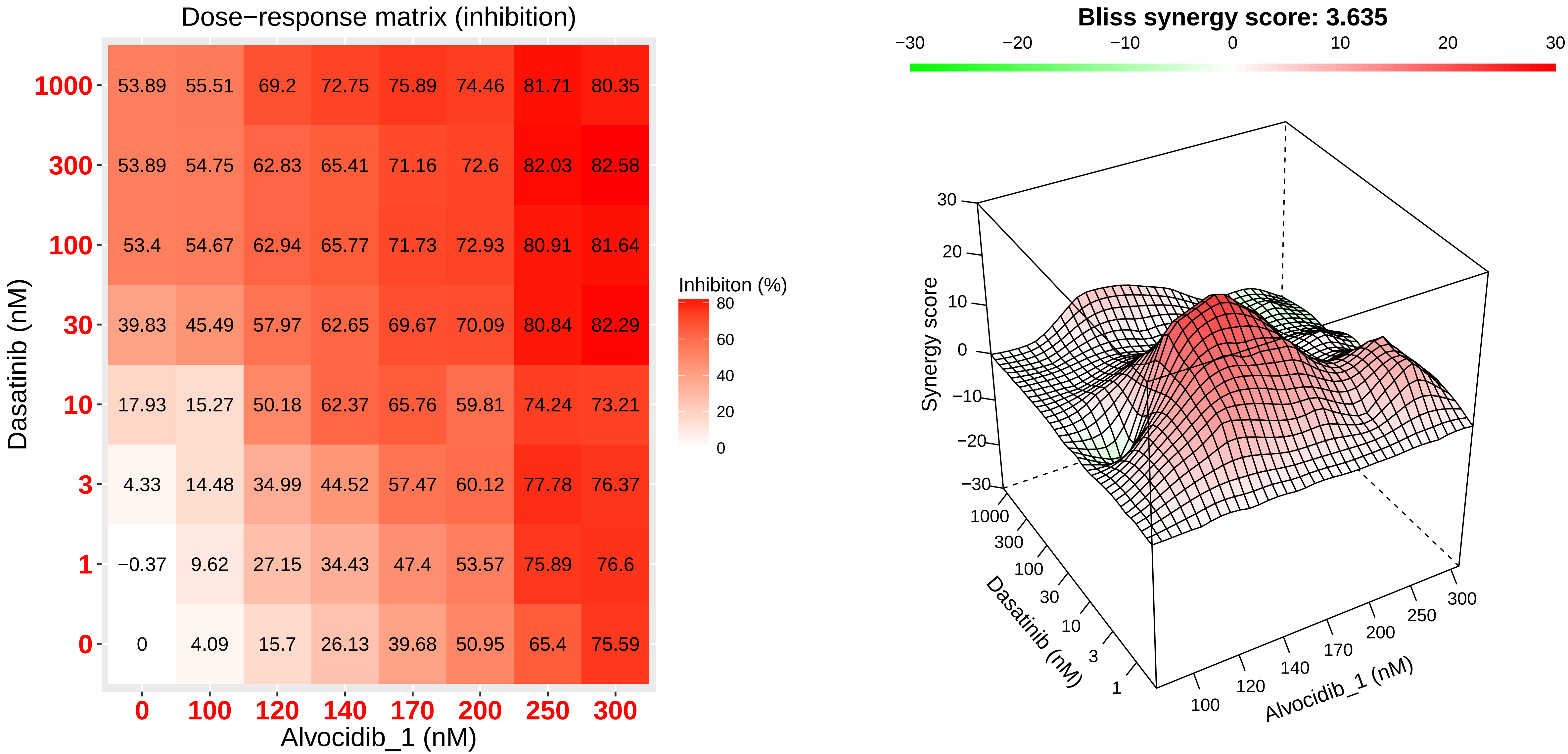
Dose−response matrix (inhibition)
Dasatinib (nM)
Alvocidib_1 (nM)
1000: 53.89 55.51 69.2 72.75 75.89 74.46 81.71 80.35
300: 53.89 54.75 62.83 65.41 71.16 72.6 82.03 82.58
100: 53.4 54.67 62.94 65.77 71.73 72.93 80.91 81.64
30: 39.83 45.49 57.97 62.65 69.67 70.09 80.84 82.29
10: 17.93 15.27 50.18 62.37 65.76 59.81 74.24 73.21
3: 4.33 14.48 34.99 44.52 57.47 60.12 77.78 76.37
1: −0.37 9.62 27.15 34.43 47.4 53.57 75.89 76.6
0: 0 4.09 15.7 26.13 39.68 50.95 65.4 75.59
0 100 120 140 170 200 250 300
Inhibiton (%)
80
60
40
20
0
Bliss synergy score: 3.635
−30 −20 −10 0 10 20 30
Synergy score
30
20
10
0
−10
−20
−30
1000
300
100
30
10
3
1
Dasatinib (nM)
100
120
140
170
200
250
300
Alvocidib_1 (nM)

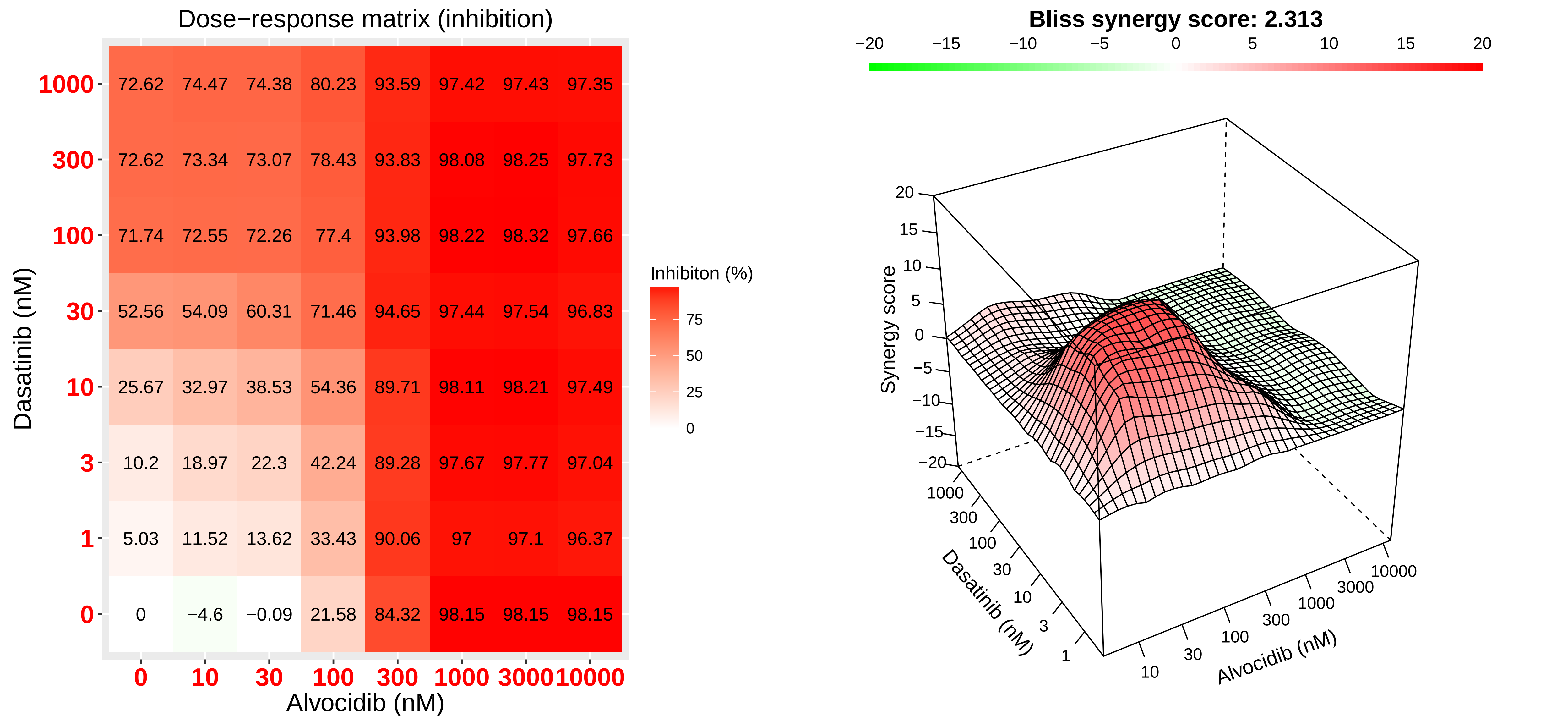

Dose−response matrix (inhibition)
Dasatinib (nM)
1000 300 100 30 10 3 1 0
72.62 74.47 74.38 80.23 93.59 97.42 97.43 97.35
72.62 73.34 73.07 78.43 93.83 98.08 98.25 97.73
71.74 72.55 72.26 77.4 93.98 98.22 98.32 97.66
52.56 54.09 60.31 71.46 94.65 97.44 97.54 96.83
25.67 32.97 38.53 54.36 89.71 98.11 98.21 97.49
10.2 18.97 22.3 42.24 89.28 97.67 97.77 97.04
5.03 11.52 13.62 33.43 90.06 97 97.1 96.37
0 −4.6 −0.09 21.58 84.32 98.15 98.15 98.15
0 10 30 100 300 1000 3000 10000
Alvocidib (nM)
Inhibiton (%)
75
50
25
0
Bliss synergy score: 2.313
−20 −15 −10 −5 0 5 10 15 20
Synergy score
20 15 10 5 0 −5 −10 −15 −20
Dasatinib (nM)
1000 300 100 30 10 3 1
Alvocidib (nM)
10 30 100 300 1000 3000 10000

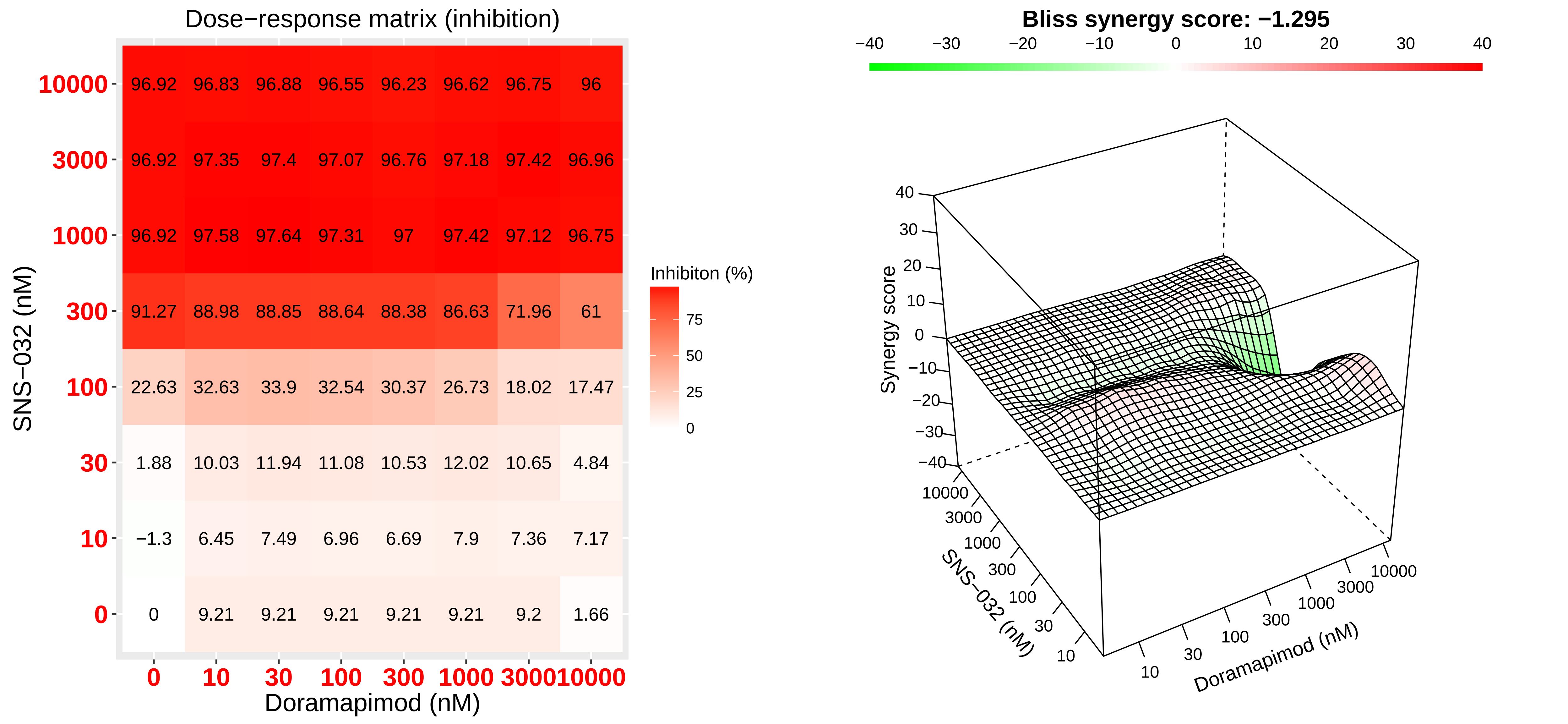
Dose−response matrix (inhibition)
10000
3000
1000
300
100
30
10
0
SNS−032 (nM)
96.92 96.83 96.88 96.55 96.23 96.62 96.75 96
96.92 97.35 97.4 97.07 96.76 97.18 97.42 96.96
96.92 97.58 97.64 97.31 97 97.42 97.12 96.75
91.27 88.98 88.85 88.64 88.38 86.63 71.96 61
22.63 32.63 33.9 32.54 30.37 26.73 18.02 17.47
1.88 10.03 11.94 11.08 10.53 12.02 10.65 4.84
−1.3 6.45 7.49 6.96 6.69 7.9 7.36 7.17
0 9.21 9.21 9.21 9.21 9.21 9.2 1.66
0 10 30 100 300 1000 3000 10000
Doramapimod (nM)
Inhibiton (%)
75
50
25
0
Bliss synergy score: −1.295
−40 −30 −20 −10 0 10 20 30 40
Synergy score
40
30
20
10
0
−10
−20
−30
−40
10000
3000
1000
300
100
30
10
SNS−032 (nM)
10
30
100
300
1000
3000
10000
Doramapimod (nM)

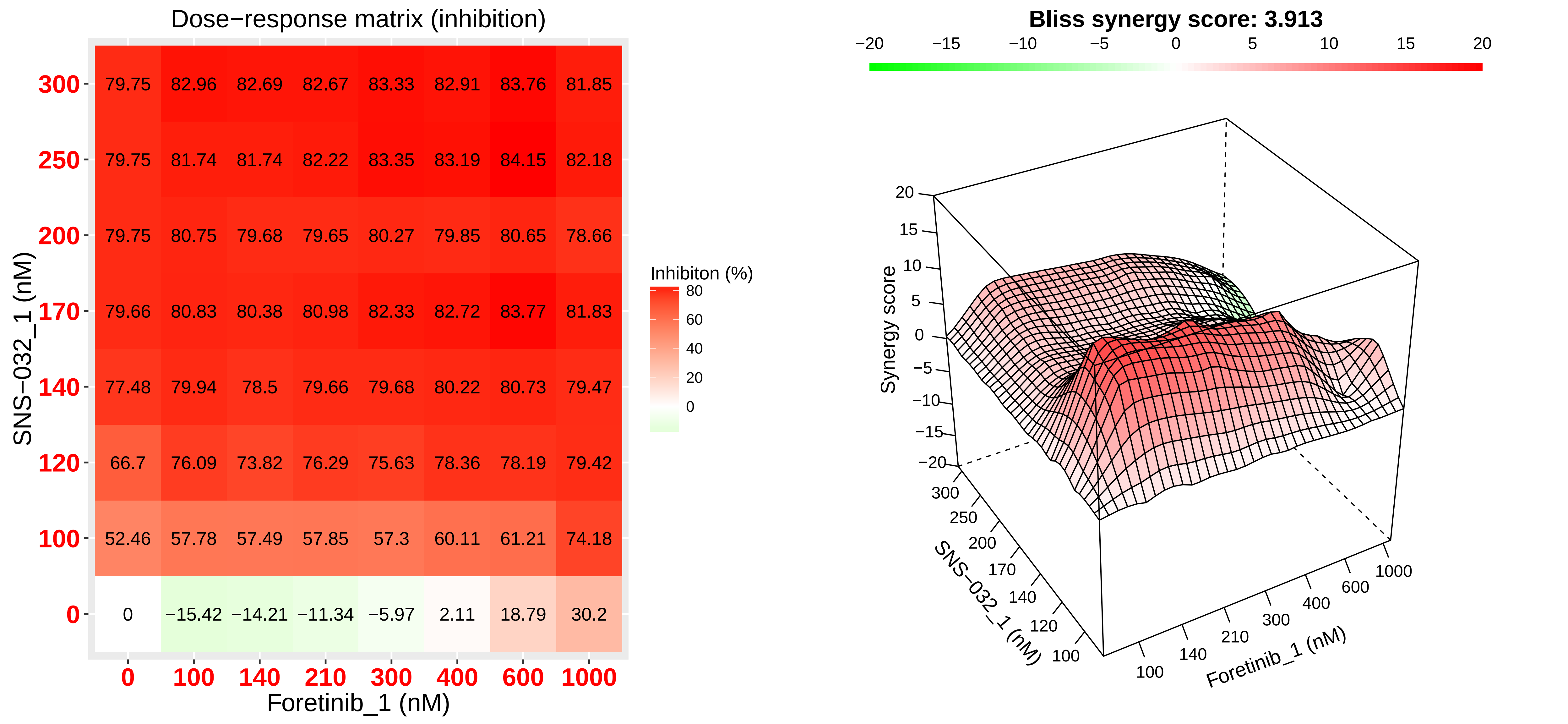
Dose−response matrix (inhibition)
300 79.75 82.96 82.69 82.67 83.33 82.91 83.76 81.85
250 79.75 81.74 81.74 82.22 83.35 83.19 84.15 82.18
200 79.75 80.75 79.68 79.65 80.27 79.85 80.65 78.66
170 79.66 80.83 80.38 80.98 82.33 82.72 83.77 81.83
140 77.48 79.94 78.5 79.66 79.68 80.22 80.73 79.47
120 66.7 76.09 73.82 76.29 75.63 78.36 78.19 79.42
100 52.46 57.78 57.49 57.85 57.3 60.11 61.21 74.18
0 0 −15.42 −14.21 −11.34 −5.97 2.11 18.79 30.2
0 100 140 210 300 400 600 1000
SNS−032_1 (nM)
Foretinib_1 (nM)
Inhibiton (%)
80 60 40 20 0
Bliss synergy score: 3.913
−20 −15 −10 −5 0 5 10 15 20
Synergy score
20 15 10 5 0 −5 −10 −15 −20
300 250 200 170 140 120 100
SNS−032_1 (nM)
100 140 210 300 400 600 1000
Foretinib_1 (nM)

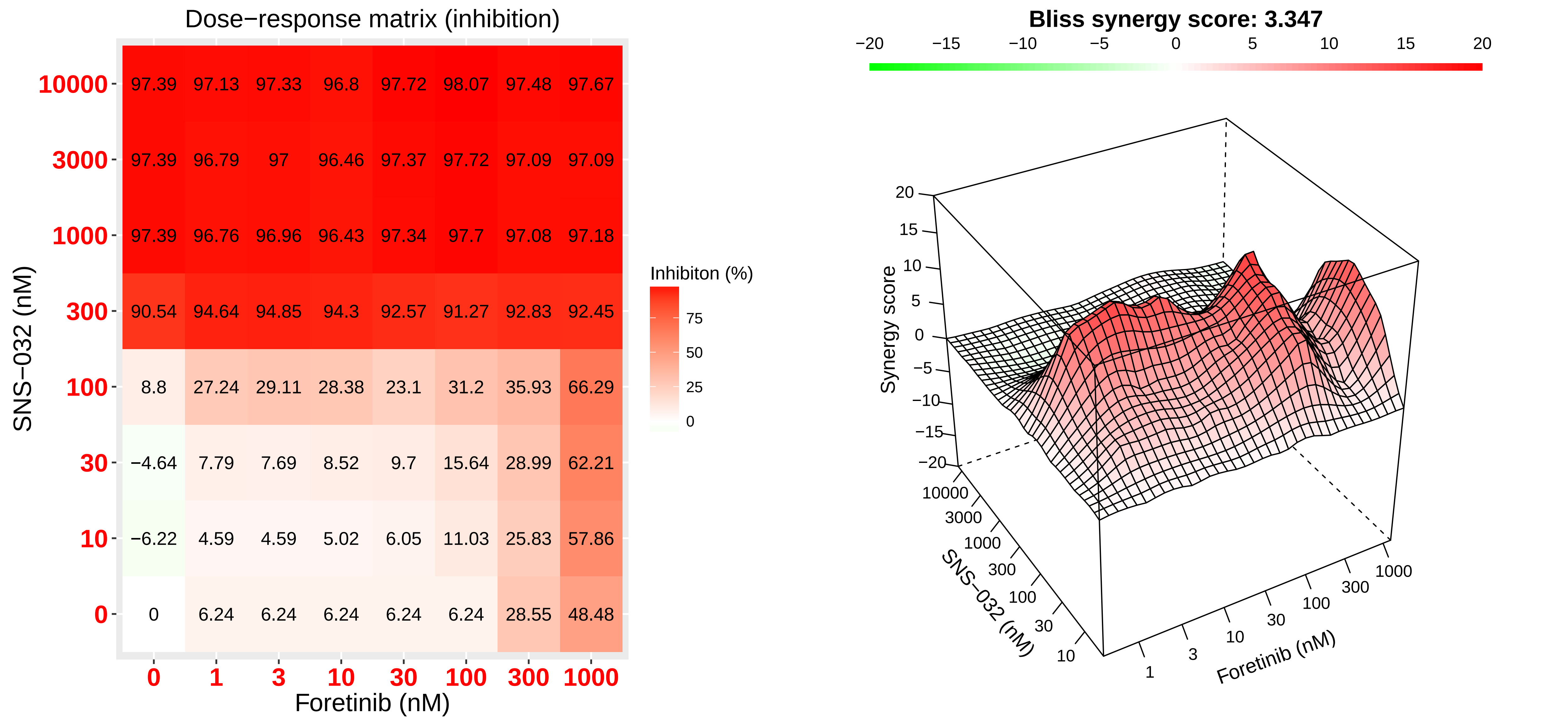
Dose−response matrix (inhibition)
SNS−032 (nM)
Foretinib (nM)
10000
3000
1000
300
100
30
10
0
0
1
3
10
30
100
300
1000
97.39 97.13 97.33 96.8 97.72 98.07 97.48 97.67
97.39 96.79 97 96.46 97.37 97.72 97.09 97.09
97.39 96.76 96.96 96.43 97.34 97.7 97.08 97.18
90.54 94.64 94.85 94.3 92.57 91.27 92.83 92.45
8.8 27.24 29.11 28.38 23.1 31.2 35.93 66.29
−4.64 7.79 7.69 8.52 9.7 15.64 28.99 62.21
−6.22 4.59 4.59 5.02 6.05 11.03 25.83 57.86
0 6.24 6.24 6.24 6.24 6.24 28.55 48.48
Inhibiton (%)
75
50
25
0
Bliss synergy score: 3.347
−20 −15 −10 −5 0 5 10 15 20
Synergy score
20
15
10
5
0
−5
−10
−15
−20
SNS−032 (nM)
10000
3000
1000
300
100
30
10
Foretinib (nM)
1
3
10
30
100
300
1000

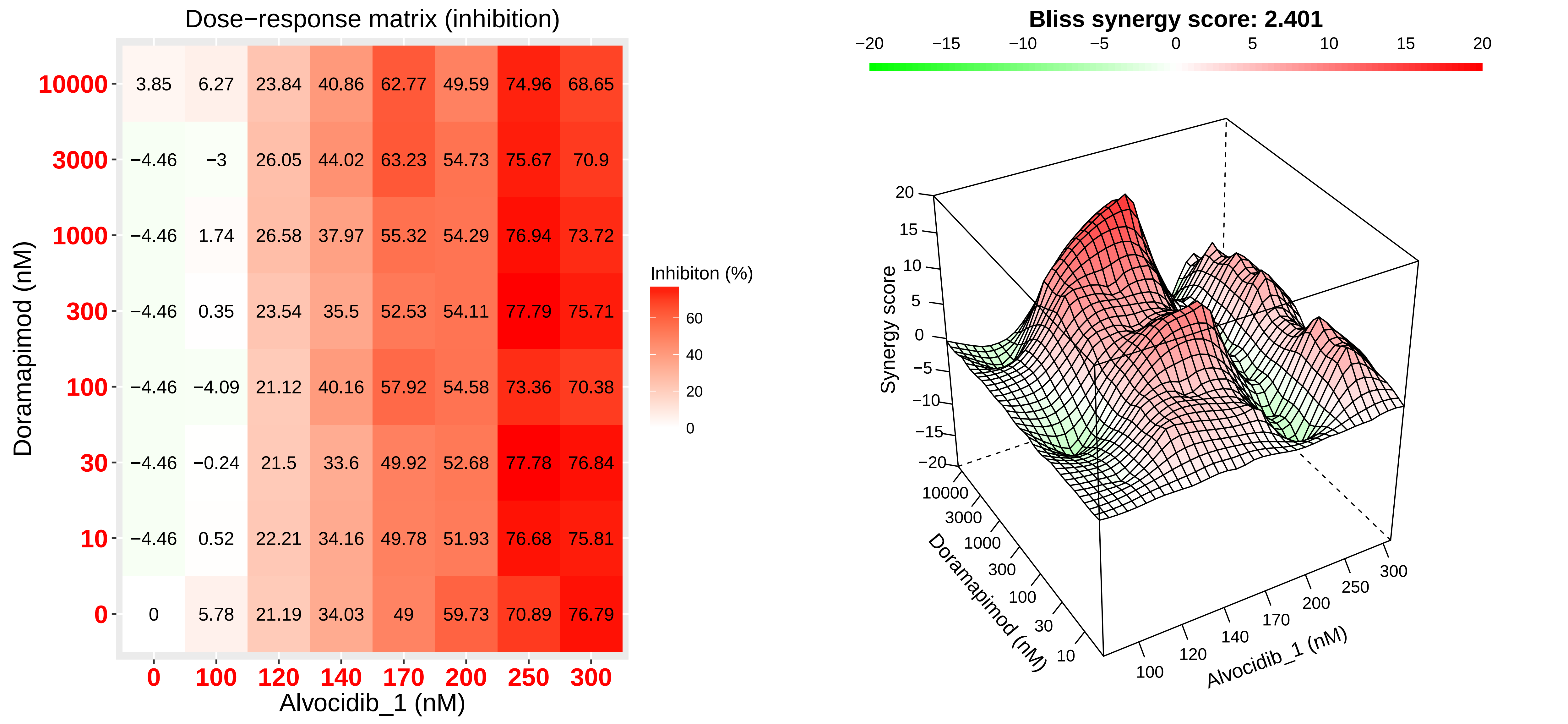

Dose−response matrix (inhibition)
10000
3.85 6.27 23.84 40.86 62.77 49.59 74.96 68.65
3000
−4.46 −3 26.05 44.02 63.23 54.73 75.67 70.9
1000
−4.46 1.74 26.58 37.97 55.32 54.29 76.94 73.72
300
−4.46 0.35 23.54 35.5 52.53 54.11 77.79 75.71
100
−4.46 −4.09 21.12 40.16 57.92 54.58 73.36 70.38
30
−4.46 −0.24 21.5 33.6 49.92 52.68 77.78 76.84
10
−4.46 0.52 22.21 34.16 49.78 51.93 76.68 75.81
0
0 5.78 21.19 34.03 49 59.73 70.89 76.79
0 100 120 140 170 200 250 300
Alvocidib_1 (nM)
Doramapimod (nM)
Inhibiton (%)
60
40
20
0
Bliss synergy score: 2.401
−20 −15 −10 −5 0 5 10 15 20
Synergy score
20 15 10 5 0 −5 −10 −15 −20
10000 3000 1000 300 100 30 10
Doramapimod (nM)
100 120 140 170 200 250 300
Alvocidib_1 (nM)

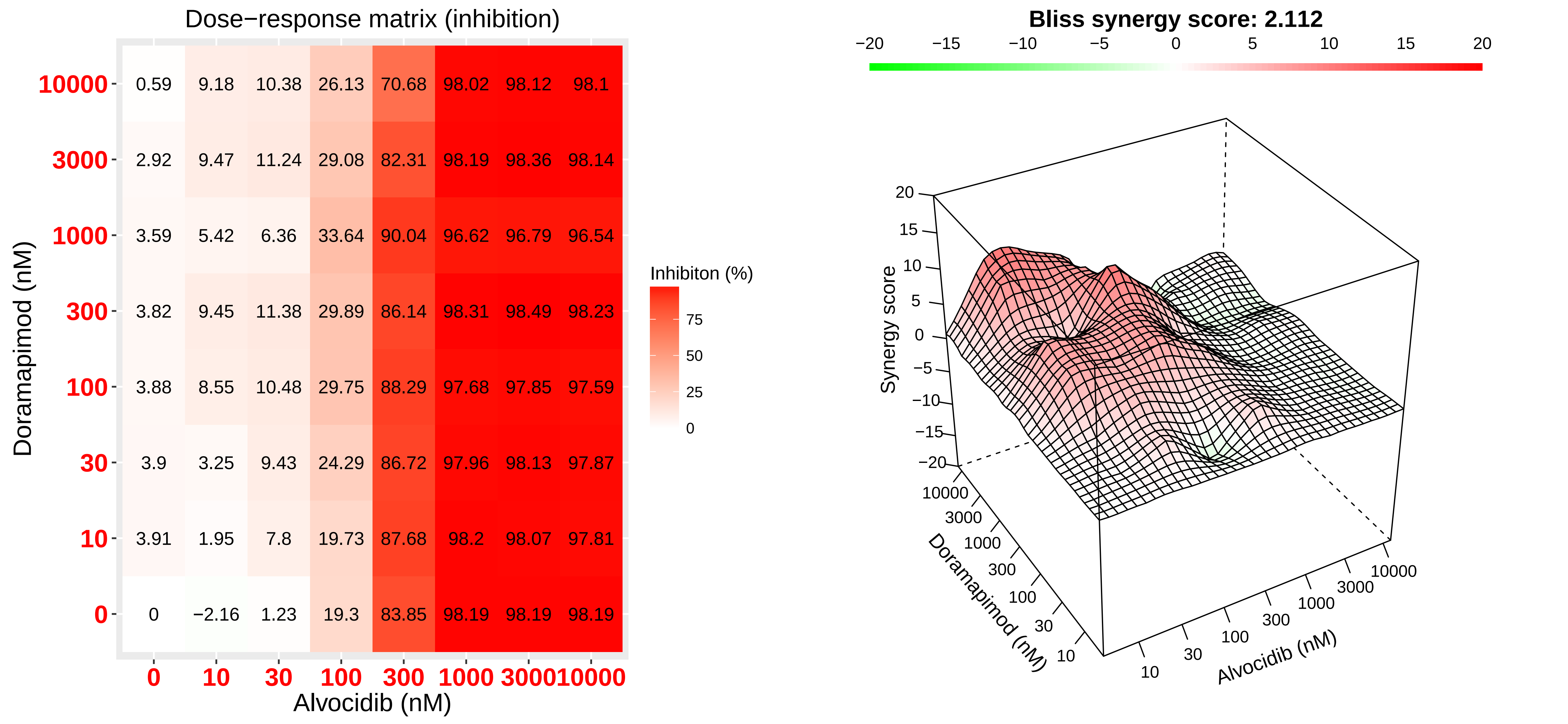

Dose−response matrix (inhibition)
Doramapimod (nM)
Alvocidib (nM)
10000 0.59 9.18 10.38 26.13 70.68 98.02 98.12 98.1
3000 2.92 9.47 11.24 29.08 82.31 98.19 98.36 98.14
1000 3.59 5.42 6.36 33.64 90.04 96.62 96.79 96.54
300 3.82 9.45 11.38 29.89 86.14 98.31 98.49 98.23
100 3.88 8.55 10.48 29.75 88.29 97.68 97.85 97.59
30 3.9 3.25 9.43 24.29 86.72 97.96 98.13 97.87
10 3.91 1.95 7.8 19.73 87.68 98.2 98.07 97.81
0 0 −2.16 1.23 19.3 83.85 98.19 98.19 98.19
0 10 30 100 300 1000 3000 10000
Inhibiton (%)
75
50
25
0
Bliss synergy score: 2.112
−20 −15 −10 −5 0 5 10 15 20
Synergy score
20 15 10 5 0 −5 −10 −15 −20
Doramapimod (nM)
10000 3000 1000 300 100 30 10
Alvocidib (nM)
10 30 100 300 1000 3000 10000

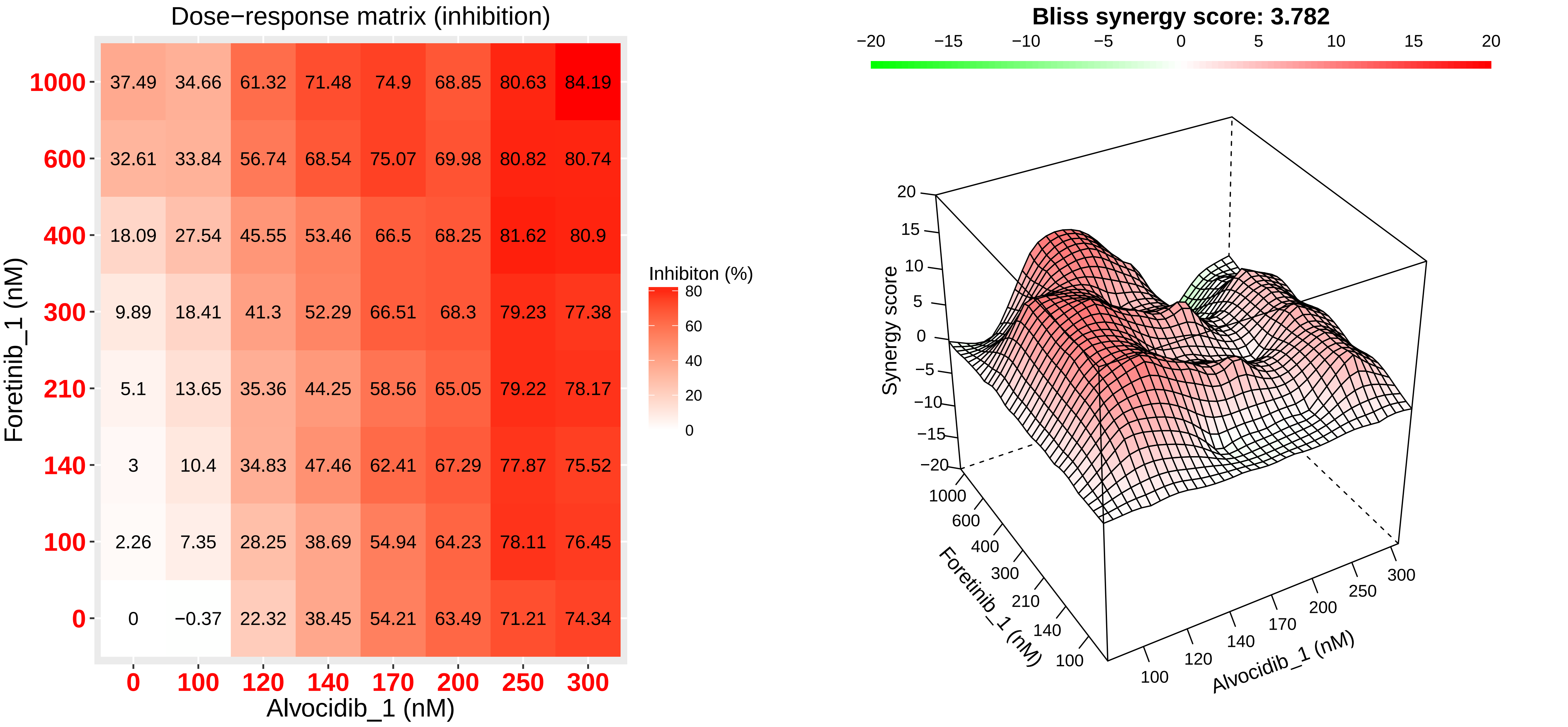
Dose−response matrix (inhibition)
1000
37.49 34.66 61.32 71.48 74.9 68.85 80.63 84.19
600
32.61 33.84 56.74 68.54 75.07 69.98 80.82 80.74
400
18.09 27.54 45.55 53.46 66.5 68.25 81.62 80.9
300
9.89 18.41 41.3 52.29 66.51 68.3 79.23 77.38
210
5.1 13.65 35.36 44.25 58.56 65.05 79.22 78.17
140
3 10.4 34.83 47.46 62.41 67.29 77.87 75.52
100
2.26 7.35 28.25 38.69 54.94 64.23 78.11 76.45
0
0 −0.37 22.32 38.45 54.21 63.49 71.21 74.34
0 100 120 140 170 200 250 300
Alvocidib_1 (nM)
Foretinib_1 (nM)
Inhibiton (%)
80
60
40
20
0
Bliss synergy score: 3.782
−20 −15 −10 −5 0 5 10 15 20
Synergy score
20
15
10
5
0
−5
−10
−15
−20
1000 600 400 300 210 140 100
Foretinib_1 (nM)
100 120 140 170 200 250 300
Alvocidib_1 (nM)

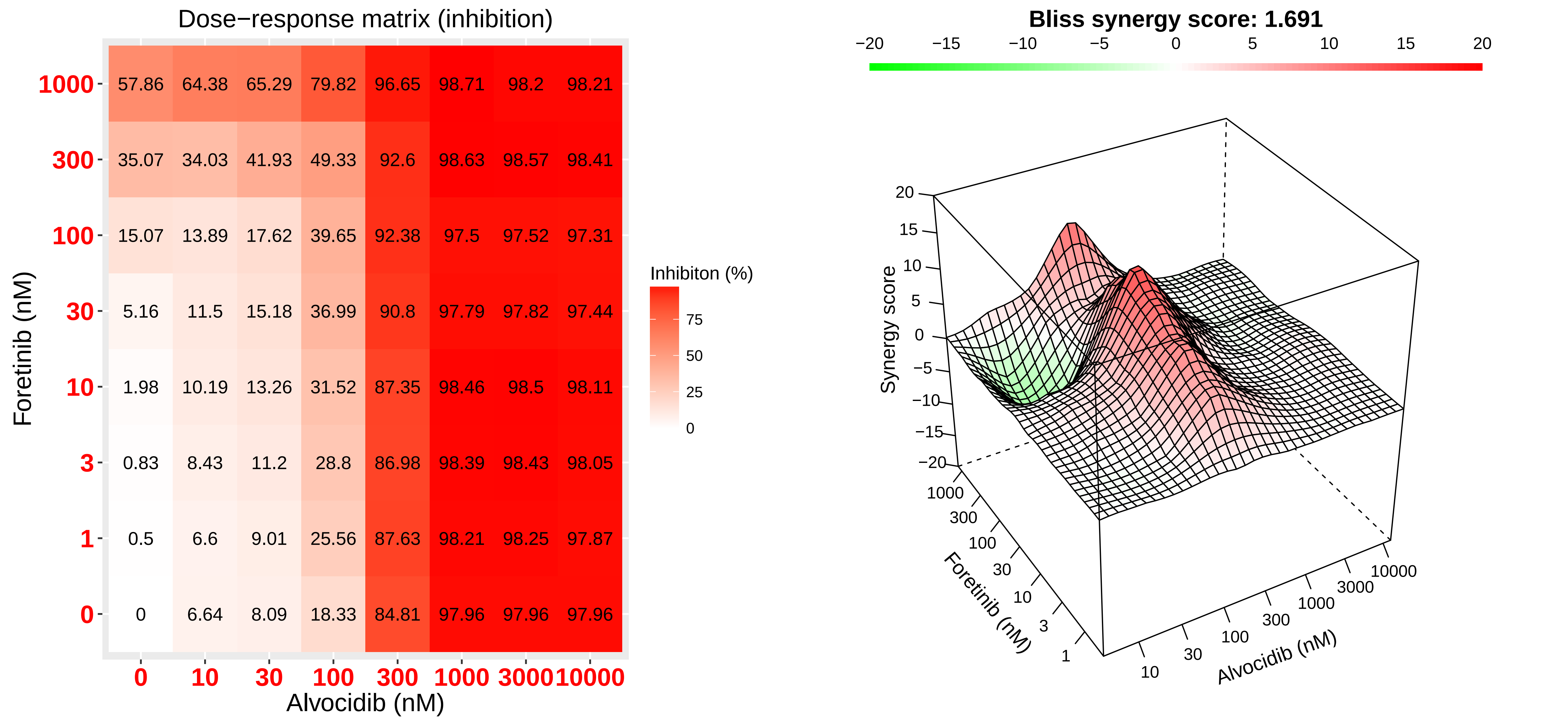
Dose−response matrix (inhibition)
Foretinib (nM)
1000
300
100
30
10
3
1
0
57.86 64.38 65.29 79.82 96.65 98.71 98.2 98.21
35.07 34.03 41.93 49.33 92.6 98.63 98.57 98.41
15.07 13.89 17.62 39.65 92.38 97.5 97.52 97.31
5.16 11.5 15.18 36.99 90.8 97.79 97.82 97.44
1.98 10.19 13.26 31.52 87.35 98.46 98.5 98.11
0.83 8.43 11.2 28.8 86.98 98.39 98.43 98.05
0.5 6.6 9.01 25.56 87.63 98.21 98.25 97.87
0 6.64 8.09 18.33 84.81 97.96 97.96 97.96
0
10
30
100
300
1000
3000
10000
Alvocidib (nM)
Inhibiton (%)
75
50
25
0
Bliss synergy score: 1.691
−20
−15
−10
−5
0
5
10
15
20
Synergy score
20
15
10
5
0
−5
−10
−15
−20
Foretinib (nM)
1000
300
100
30
10
3
1
Alvocidib (nM)
10
30
100
300
1000
3000
10000

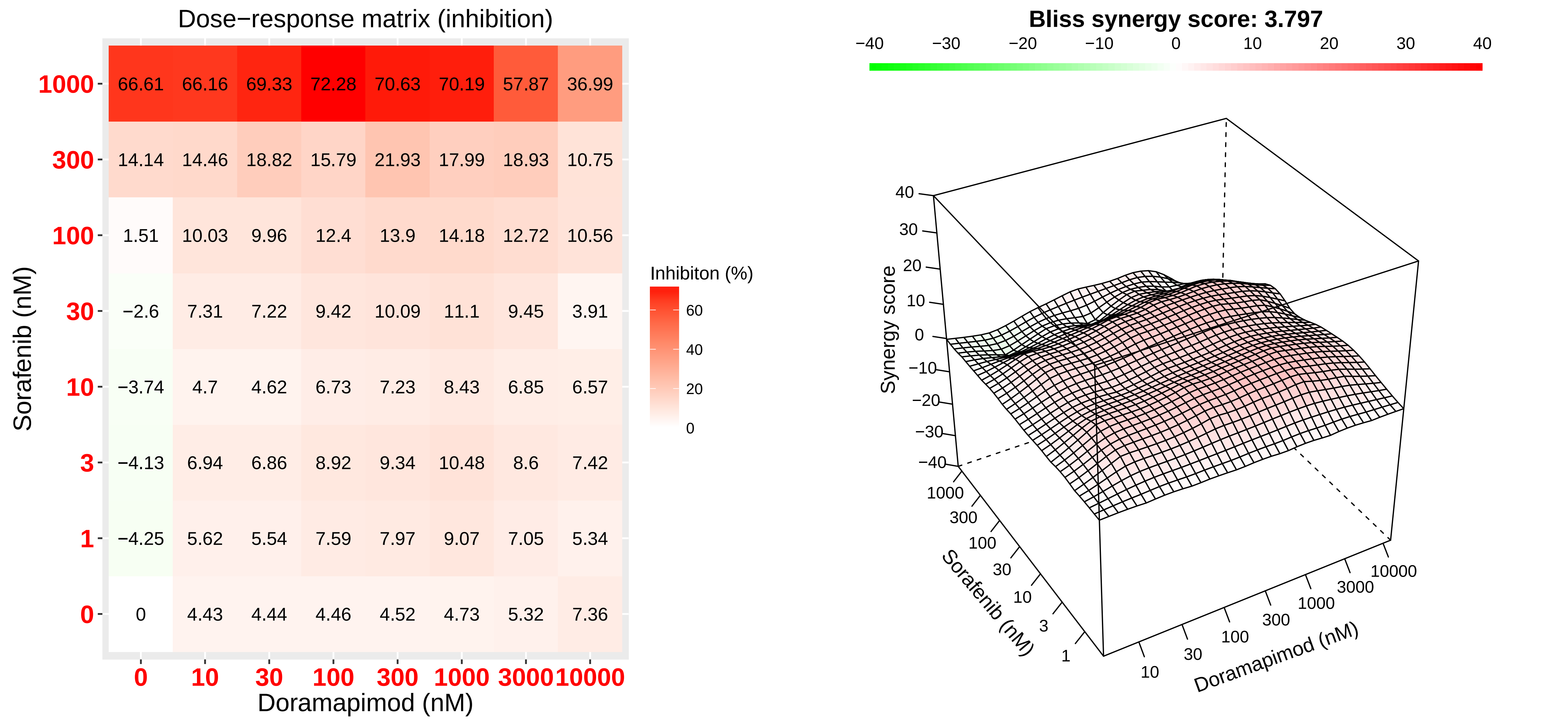

Dose−response matrix (inhibition)
1000 66.61 66.16 69.33 72.28 70.63 70.19 57.87 36.99
300 14.14 14.46 18.82 15.79 21.93 17.99 18.93 10.75
100 1.51 10.03 9.96 12.4 13.9 14.18 12.72 10.56
30 −2.6 7.31 7.22 9.42 10.09 11.1 9.45 3.91
10 −3.74 4.7 4.62 6.73 7.23 8.43 6.85 6.57
3 −4.13 6.94 6.86 8.92 9.34 10.48 8.6 7.42
1 −4.25 5.62 5.54 7.59 7.97 9.07 7.05 5.34
0 0 4.43 4.44 4.46 4.52 4.73 5.32 7.36
0 10 30 100 300 1000 3000 10000
Doramapimod (nM)
Sorafenib (nM)
Inhibiton (%)
60
40
20
0
Bliss synergy score: 3.797
−40 −30 −20 −10 0 10 20 30 40
Synergy score
40 30 20 10 0 −10 −20 −30 −40
1000 300 100 30 10 3 1
Sorafenib (nM)
10 30 100 300 1000 3000 10000
Doramapimod (nM)

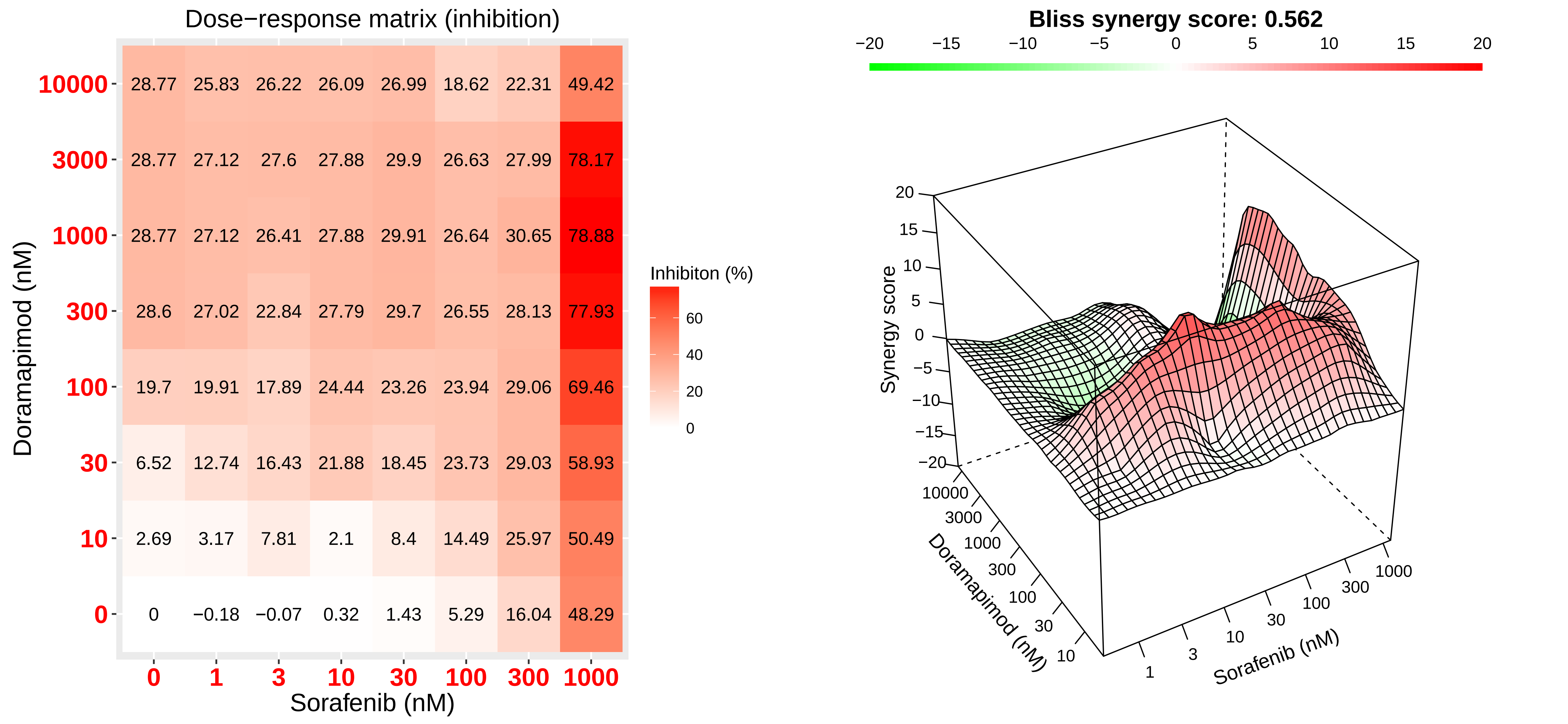
Dose−response matrix (inhibition)
10000
3000
1000
300
100
30
10
0
Doramapimod (nM)
28.77 25.83 26.22 26.09 26.99 18.62 22.31 49.42
28.77 27.12 27.6 27.88 29.9 26.63 27.99 78.17
28.77 27.12 26.41 27.88 29.91 26.64 30.65 78.88
28.6 27.02 22.84 27.79 29.7 26.55 28.13 77.93
19.7 19.91 17.89 24.44 23.26 23.94 29.06 69.46
6.52 12.74 16.43 21.88 18.45 23.73 29.03 58.93
2.69 3.17 7.81 2.1 8.4 14.49 25.97 50.49
0 −0.18 −0.07 0.32 1.43 5.29 16.04 48.29
0 1 3 10 30 100 300 1000
Sorafenib (nM)
Inhibiton (%)
60
40
20
0
Bliss synergy score: 0.562
−20 −15 −10 −5 0 5 10 15 20
Synergy score
20
15
10
5
0
−5
−10
−15
−20
10000
3000
1000
300
100
30
10
Doramapimod (nM)
1
3
10
30
100
300
1000
Sorafenib (nM)

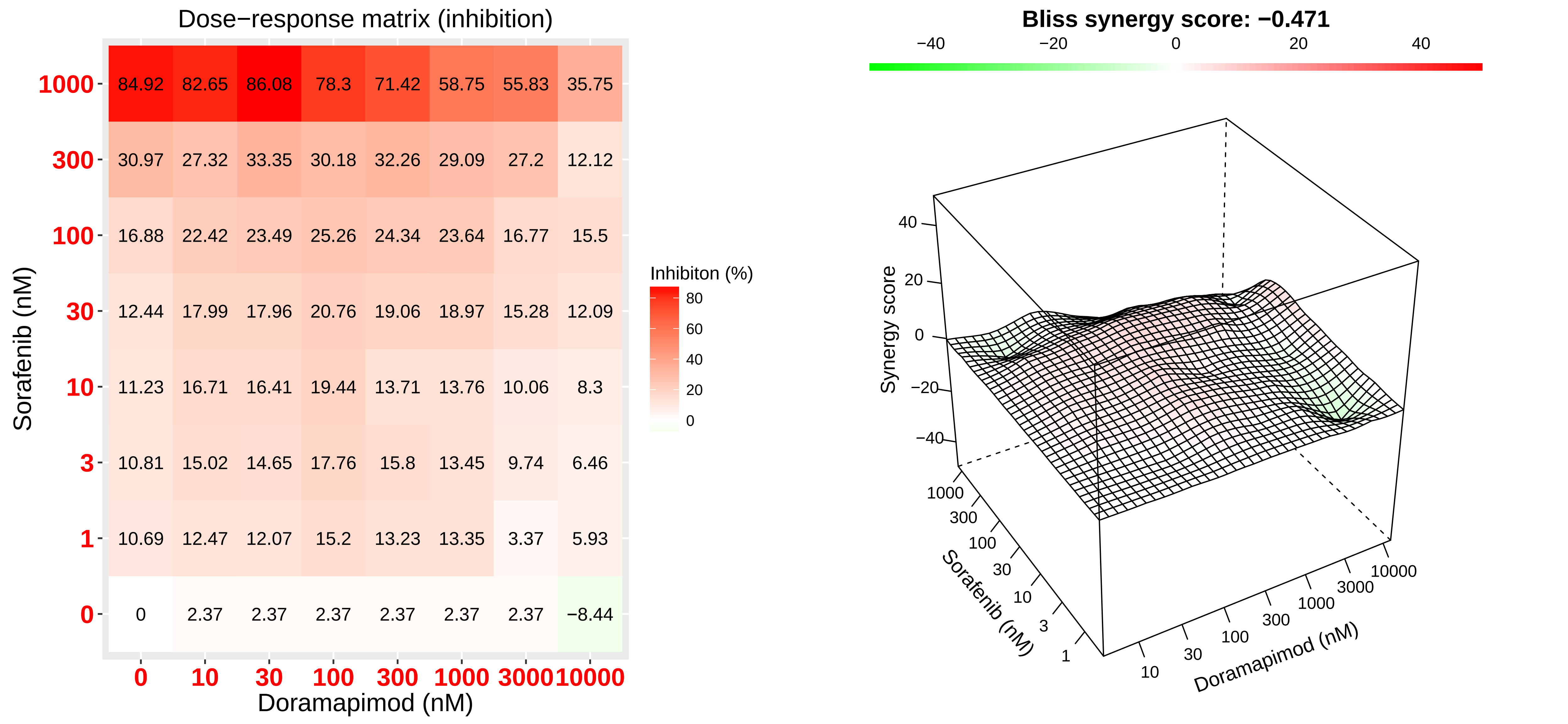

Dose−response matrix (inhibition)
1000
84.92 82.65 86.08 78.3 71.42 58.75 55.83 35.75
300
30.97 27.32 33.35 30.18 32.26 29.09 27.2 12.12
100
16.88 22.42 23.49 25.26 24.34 23.64 16.77 15.5
30
12.44 17.99 17.96 20.76 19.06 18.97 15.28 12.09
10
11.23 16.71 16.41 19.44 13.71 13.76 10.06 8.3
3
10.81 15.02 14.65 17.76 15.8 13.45 9.74 6.46
1
10.69 12.47 12.07 15.2 13.23 13.35 3.37 5.93
0
0 2.37 2.37 2.37 2.37 2.37 2.37 −8.44
Sorafenib (nM)
0 10 30 100 300 1000 3000 10000
Doramapimod (nM)
Inhibiton (%)
80
60
40
20
0
Bliss synergy score: −0.471
−40 −20 0 20 40
Synergy score
40
20
0
−20
−40
1000 300 100 30 10 3 1
Sorafenib (nM)
10 30 100 300 1000 3000 10000
Doramapimod (nM)

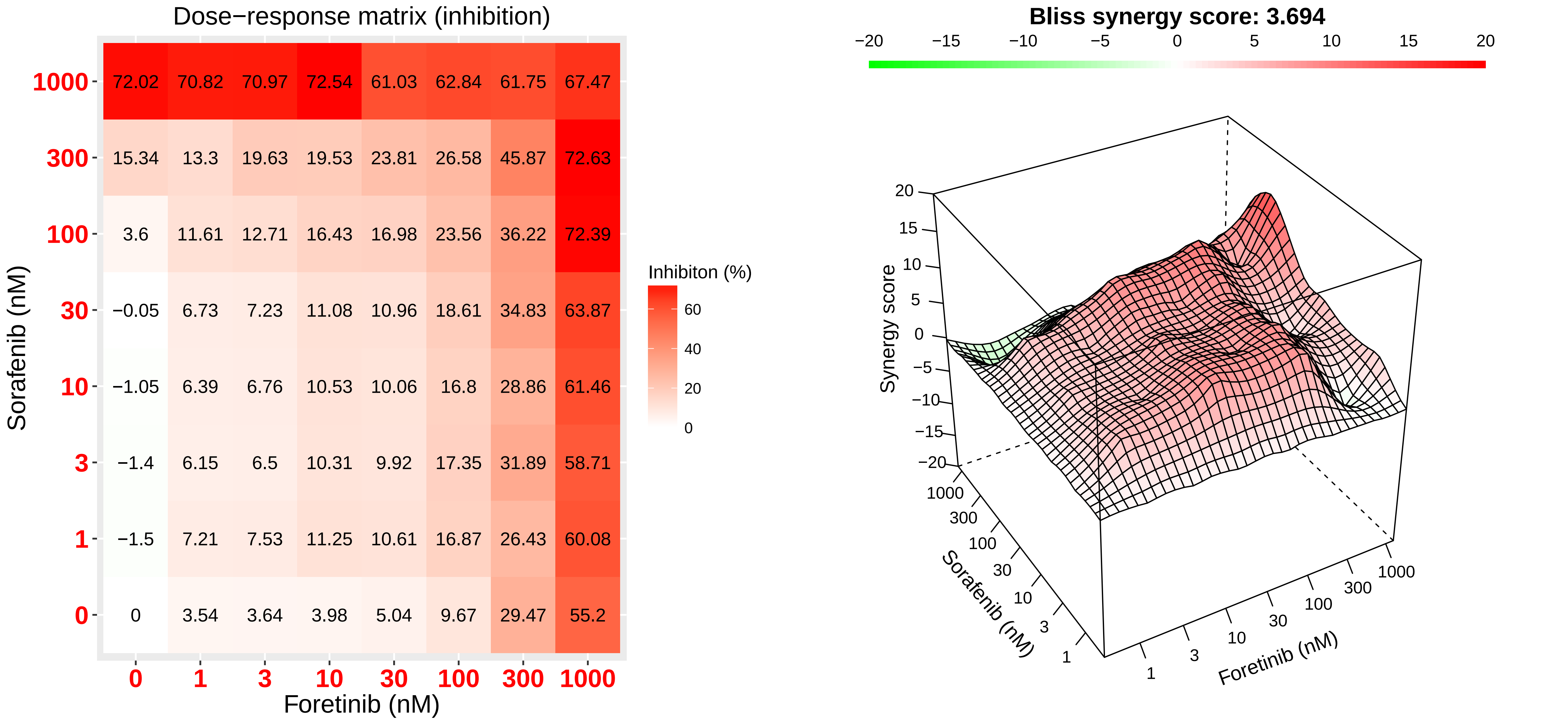

Dose−response matrix (inhibition)
Sorafenib (nM)
1000
300
100
30
10
3
1
0
72.02 70.82 70.97 72.54 61.03 62.84 61.75 67.47
15.34 13.3 19.63 19.53 23.81 26.58 45.87 72.63
3.6 11.61 12.71 16.43 16.98 23.56 36.22 72.39
−0.05 6.73 7.23 11.08 10.96 18.61 34.83 63.87
−1.05 6.39 6.76 10.53 10.06 16.8 28.86 61.46
−1.4 6.15 6.5 10.31 9.92 17.35 31.89 58.71
−1.5 7.21 7.53 11.25 10.61 16.87 26.43 60.08
0 3.54 3.64 3.98 5.04 9.67 29.47 55.2
0 1 3 10 30 100 300 1000
Foretinib (nM)
Inhibiton (%)
60
40
20
0
Bliss synergy score: 3.694
−20 −15 −10 −5 0 5 10 15 20
Synergy score
20
15
10
5
0
−5
−10
−15
−20
Sorafenib (nM)
1000
300
100
30
10
3
1
Foretinib (nM)
1
3
10
30
100
300
1000

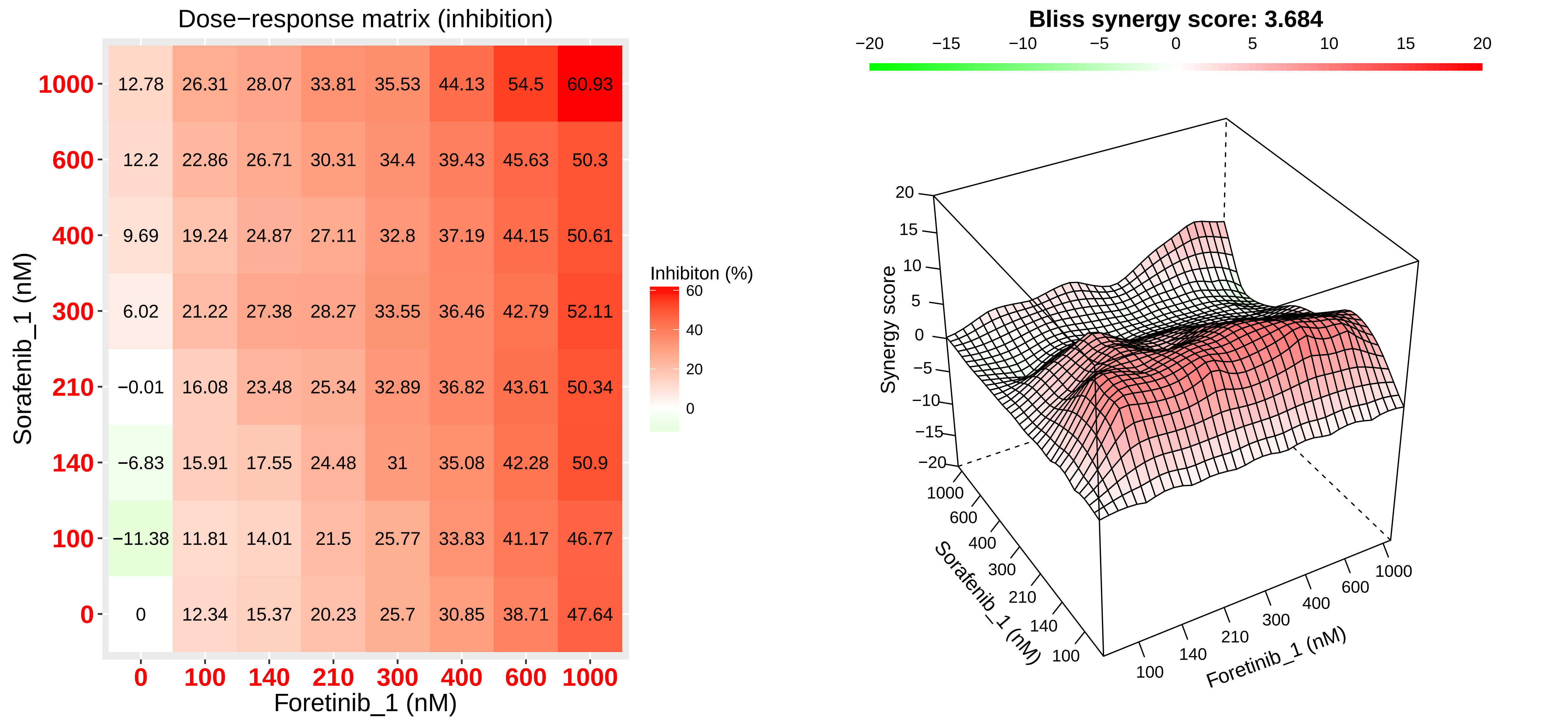
Dose−response matrix (inhibition)
1000 12.78 26.31 28.07 33.81 35.53 44.13 54.5 60.93
600 12.2 22.86 26.71 30.31 34.4 39.43 45.63 50.3
400 9.69 19.24 24.87 27.11 32.8 37.19 44.15 50.61
300 6.02 21.22 27.38 28.27 33.55 36.46 42.79 52.11
210 −0.01 16.08 23.48 25.34 32.89 36.82 43.61 50.34
140 −6.83 15.91 17.55 24.48 31 35.08 42.28 50.9
100 −11.38 11.81 14.01 21.5 25.77 33.83 41.17 46.77
0 0 12.34 15.37 20.23 25.7 30.85 38.71 47.64
0 100 140 210 300 400 600 1000
Foretinib_1 (nM)
Sorafenib_1 (nM)
Inhibiton (%)
60
40
20
0
Bliss synergy score: 3.684
−20 −15 −10 −5 0 5 10 15 20
Synergy score
20
15
10
5
0
−5
−10
−15
−20
Sorafenib_1 (nM)
1000 600 400 300 210 140 100
Foretinib_1 (nM)
100 140 210 300 400 600 1000

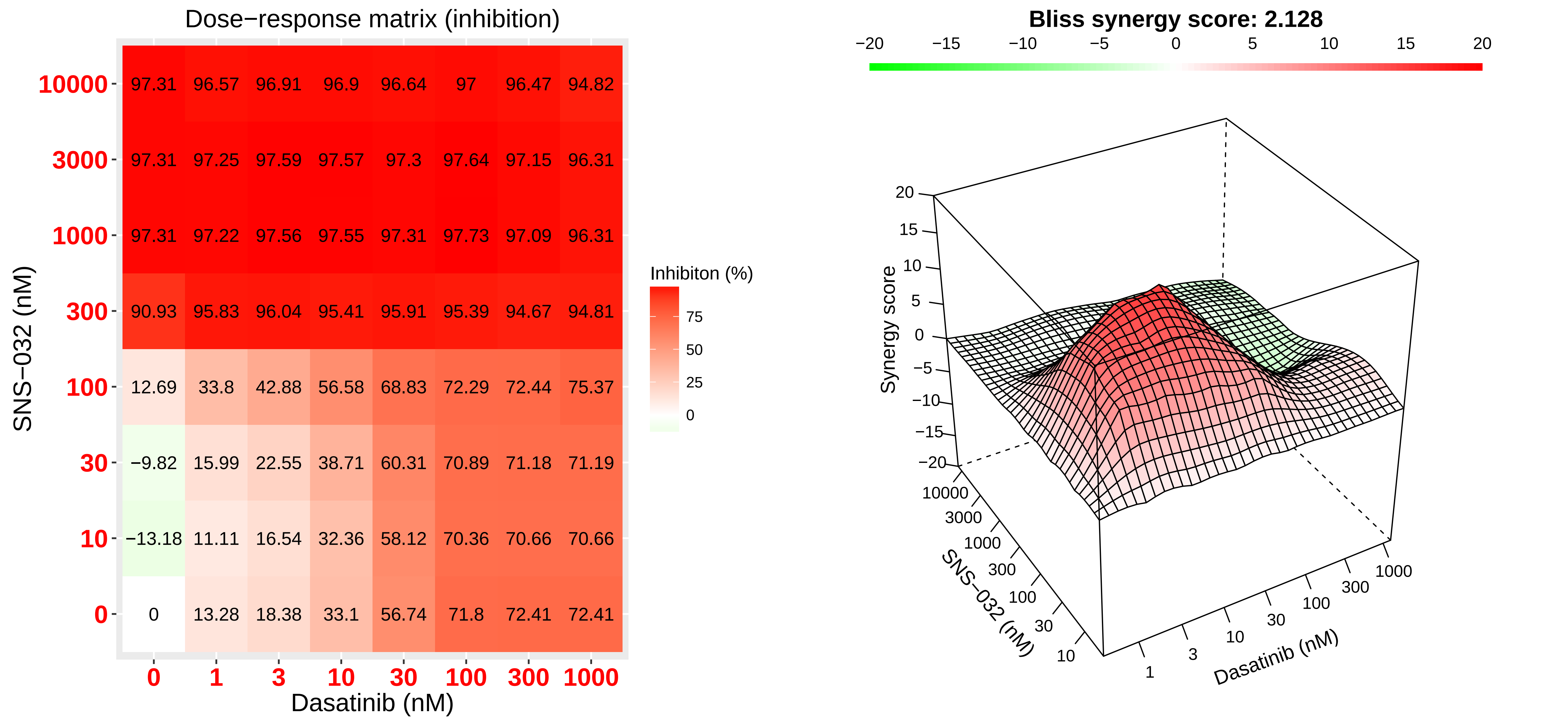
Dose−response matrix (inhibition)
10000
97.31 96.57 96.91 96.9 96.64 97 96.47 94.82
3000
97.31 97.25 97.59 97.57 97.3 97.64 97.15 96.31
1000
97.31 97.22 97.56 97.55 97.31 97.73 97.09 96.31
300
90.93 95.83 96.04 95.41 95.91 95.39 94.67 94.81
100
12.69 33.8 42.88 56.58 68.83 72.29 72.44 75.37
30
−9.82 15.99 22.55 38.71 60.31 70.89 71.18 71.19
10
−13.18 11.11 16.54 32.36 58.12 70.36 70.66 70.66
0
0 13.28 18.38 33.1 56.74 71.8 72.41 72.41
0 1 3 10 30 100 300 1000
Dasatinib (nM)
SNS−032 (nM)
Inhibiton (%)
75
50
25
0
Bliss synergy score: 2.128
−20 −15 −10 −5 0 5 10 15 20
Synergy score
20
15
10
5
0
−5
−10
−15
−20
10000
3000
1000
300
100
30
10
SNS−032 (nM)
1
3
10
30
100
300
1000
Dasatinib (nM)

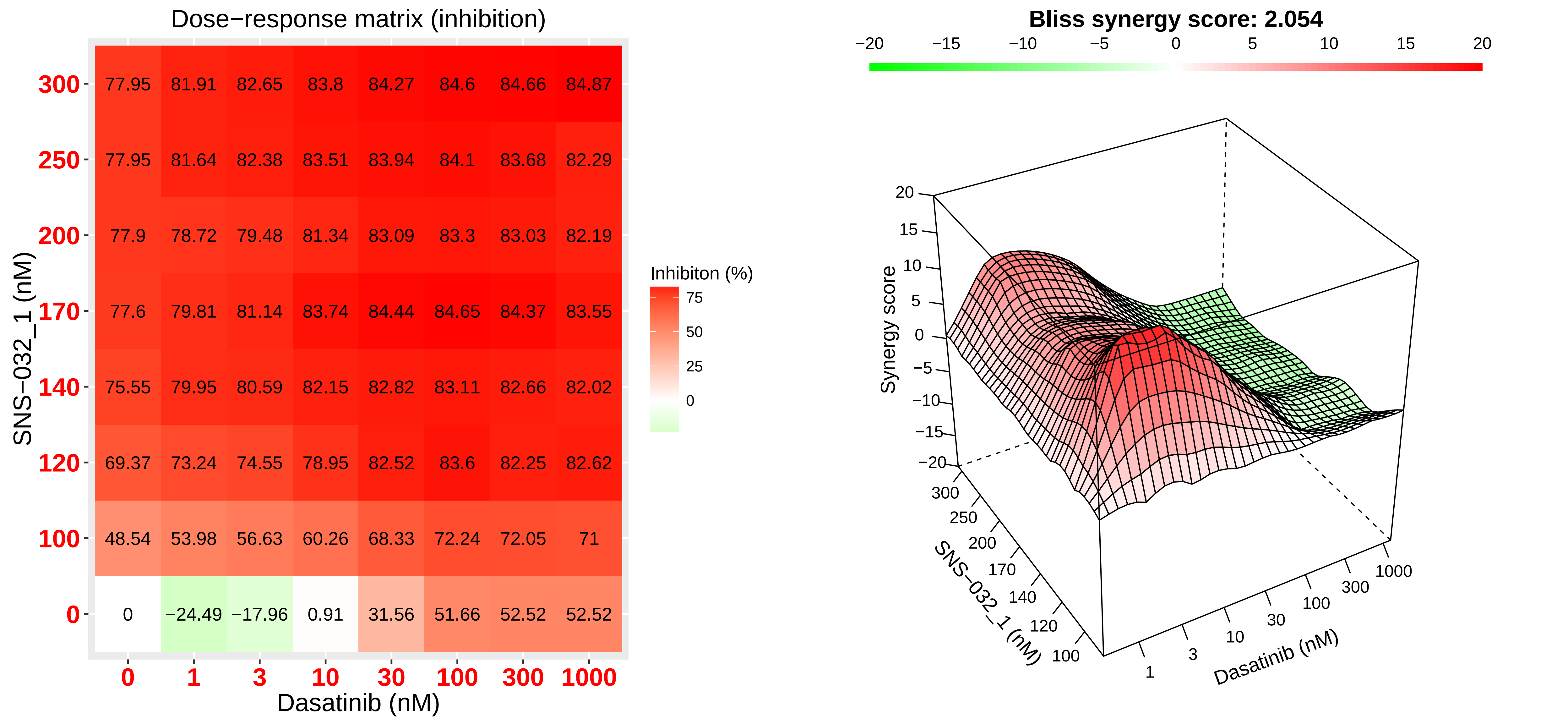
Dose−response matrix (inhibition)
300 250 200 170 140 120 100 0
SNS−032_1 (nM)
77.95 81.91 82.65 83.8 84.27 84.6 84.66 84.87
77.95 81.64 82.38 83.51 83.94 84.1 83.68 82.29
77.9 78.72 79.48 81.34 83.09 83.3 83.03 82.19
77.6 79.81 81.14 83.74 84.44 84.65 84.37 83.55
75.55 79.95 80.59 82.15 82.82 83.11 82.66 82.02
69.37 73.24 74.55 78.95 82.52 83.6 82.25 82.62
48.54 53.98 56.63 60.26 68.33 72.24 72.05 71
0 −24.49 −17.96 0.91 31.56 51.66 52.52 52.52
0 1 3 10 30 100 300 1000
Dasatinib (nM)
Inhibiton (%)
75 50 25 0
Bliss synergy score: 2.054
−20 −15 −10 −5 0 5 10 15 20
Synergy score
20 15 10 5 0 −5 −10 −15 −20
300 250 200 170 140 120 100
SNS−032_1 (nM)
1 3 10 30 100 300 1000
Dasatinib (nM)

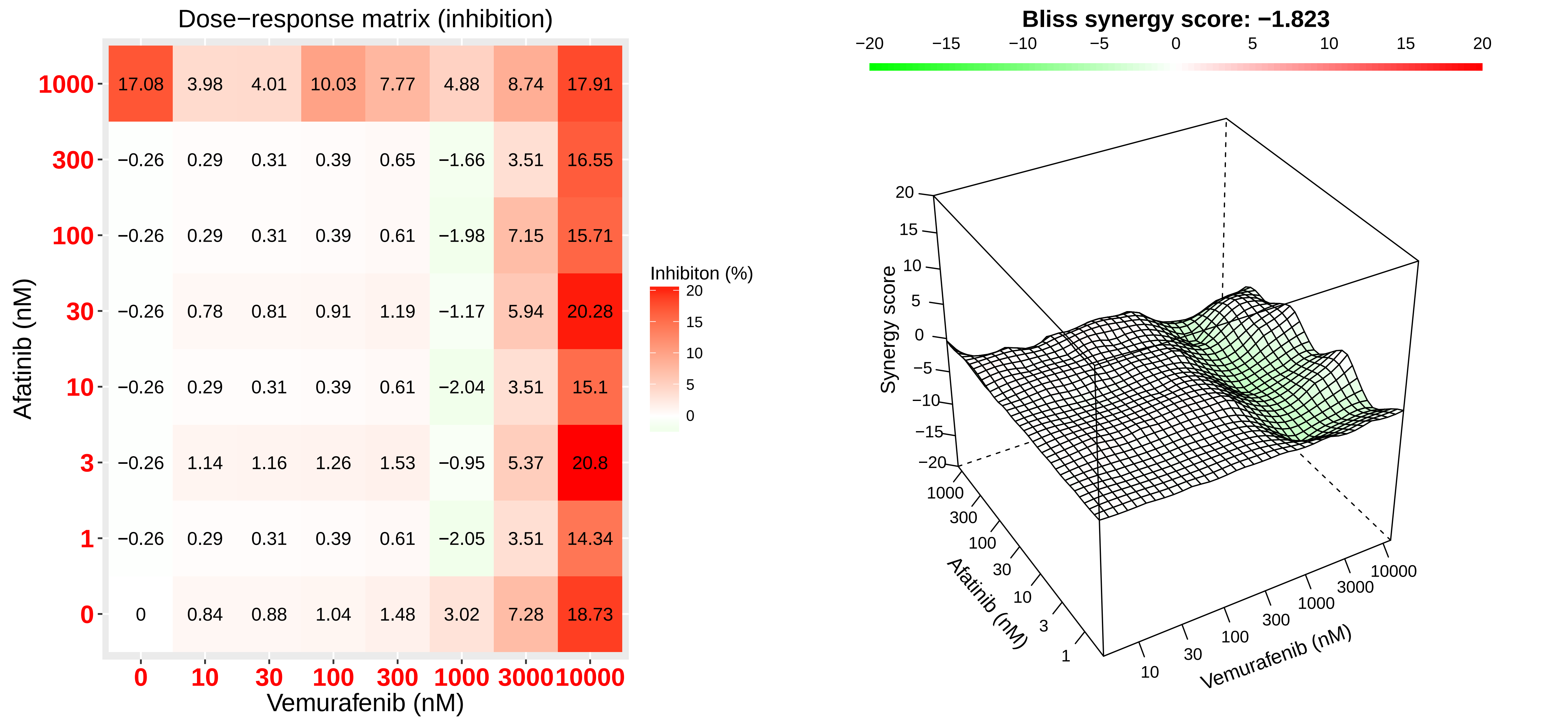
Dose−response matrix (inhibition)
1000 17.08 3.98 4.01 10.03 7.77 4.88 8.74 17.91
300 −0.26 0.29 0.31 0.39 0.65 −1.66 3.51 16.55
100 −0.26 0.29 0.31 0.39 0.61 −1.98 7.15 15.71
30 −0.26 0.78 0.81 0.91 1.19 −1.17 5.94 20.28
10 −0.26 0.29 0.31 0.39 0.61 −2.04 3.51 15.1
3 −0.26 1.14 1.16 1.26 1.53 −0.95 5.37 20.8
1 −0.26 0.29 0.31 0.39 0.61 −2.05 3.51 14.34
0 0 0.84 0.88 1.04 1.48 3.02 7.28 18.73
Afatinib (nM)
0 10 30 100 300 1000 3000 10000
Vemurafenib (nM)
Inhibiton (%)
20 15 10 5 0
Bliss synergy score: −1.823
−20 −15 −10 −5 0 5 10 15 20
Synergy score
20 15 10 5 0 −5 −10 −15 −20
Afatinib (nM)
1000 300 100 30 10 3 1
Vemurafenib (nM)
10 30 100 300 1000 3000 10000

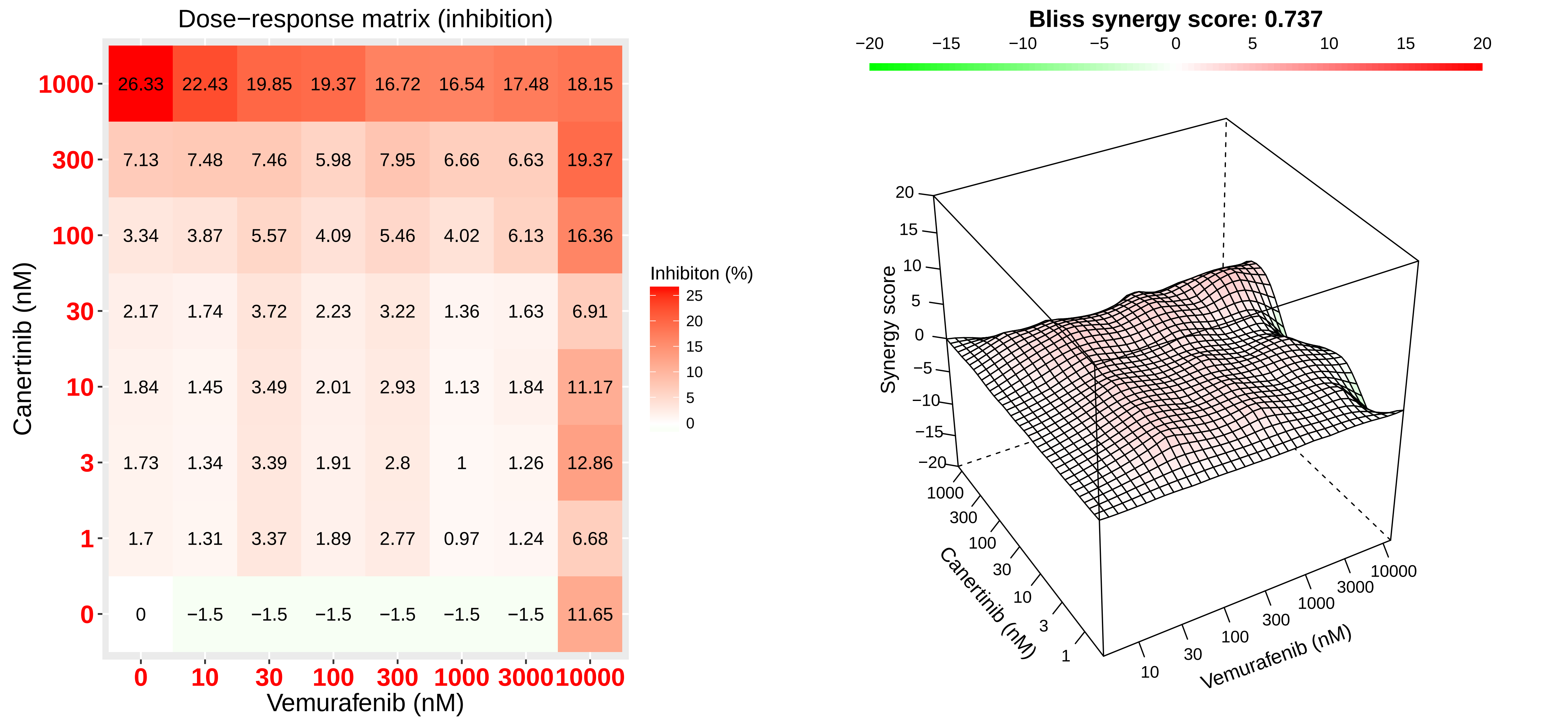
Dose−response matrix (inhibition)
1000 26.33 22.43 19.85 19.37 16.72 16.54 17.48 18.15
300 7.13 7.48 7.46 5.98 7.95 6.66 6.63 19.37
100 3.34 3.87 5.57 4.09 5.46 4.02 6.13 16.36
30 2.17 1.74 3.72 2.23 3.22 1.36 1.63 6.91
10 1.84 1.45 3.49 2.01 2.93 1.13 1.84 11.17
3 1.73 1.34 3.39 1.91 2.8 1 1.26 12.86
1 1.7 1.31 3.37 1.89 2.77 0.97 1.24 6.68
0 0 −1.5 −1.5 −1.5 −1.5 −1.5 −1.5 11.65
0 10 30 100 300 1000 3000 10000
Canertinib (nM)
Vemurafenib (nM)
Inhibiton (%)
25 20 15 10 5 0
Bliss synergy score: 0.737
−20 −15 −10 −5 0 5 10 15 20
Synergy score
20 15 10 5 0 −5 −10 −15 −20
1000 300 100 30 10 3 1
Canertinib (nM)
10 30 100 300 1000 3000 10000
Vemurafenib (nM)

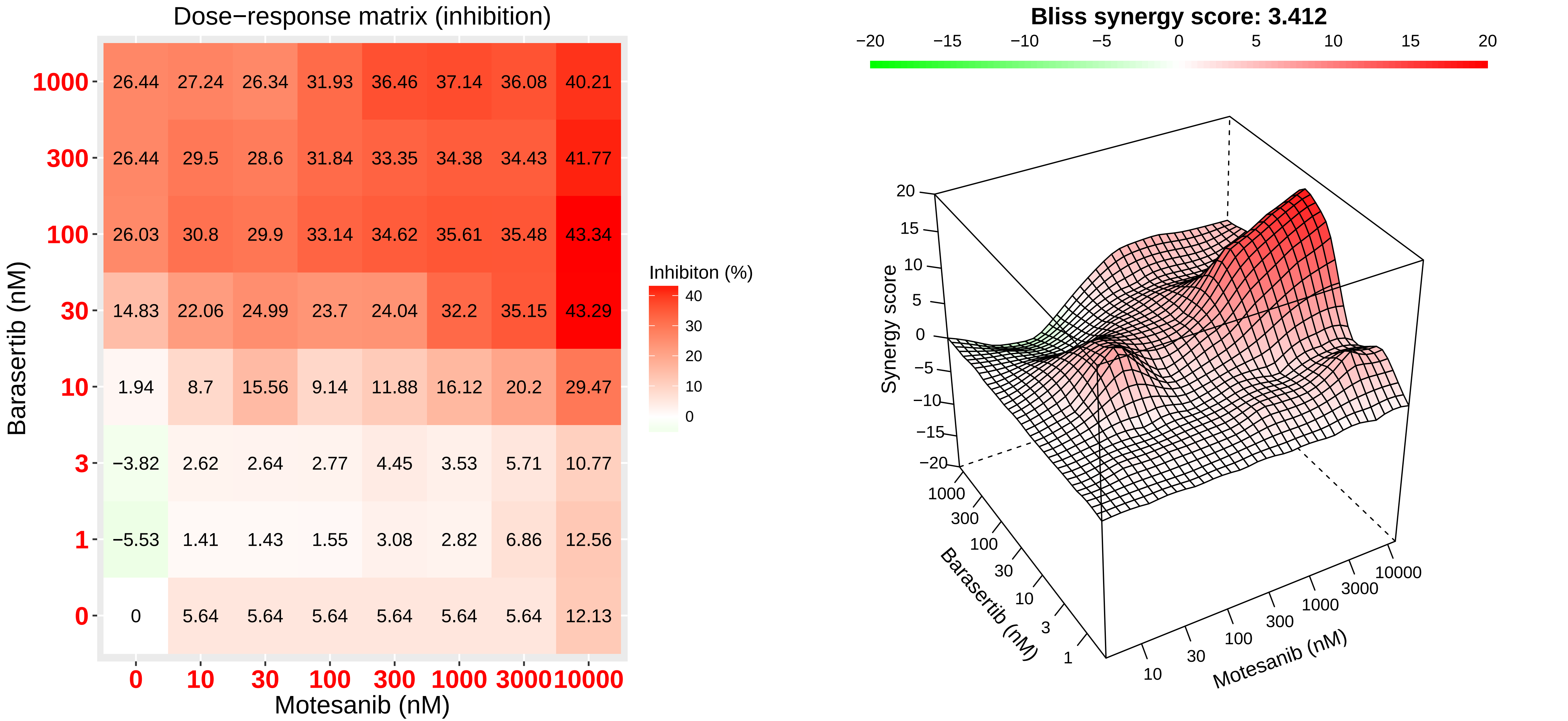
Dose−response matrix (inhibition)
1000
300
100
30
10
3
1
0
Barasertib (nM)
26.44 27.24 26.34 31.93 36.46 37.14 36.08 40.21
26.44 29.5 28.6 31.84 33.35 34.38 34.43 41.77
26.03 30.8 29.9 33.14 34.62 35.61 35.48 43.34
14.83 22.06 24.99 23.7 24.04 32.2 35.15 43.29
1.94 8.7 15.56 9.14 11.88 16.12 20.2 29.47
−3.82 2.62 2.64 2.77 4.45 3.53 5.71 10.77
−5.53 1.41 1.43 1.55 3.08 2.82 6.86 12.56
0 5.64 5.64 5.64 5.64 5.64 5.64 12.13
0 10 30 100 300 1000 3000 10000
Motesanib (nM)
Inhibiton (%)
40
30
20
10
0
Bliss synergy score: 3.412
−20 −15 −10 −5 0 5 10 15 20
Synergy score
20
15
10
5
0
−5
−10
−15
−20
Barasertib (nM)
1000
300
100
30
10
3
1
Motesanib (nM)
10
30
100
300
1000
3000
10000

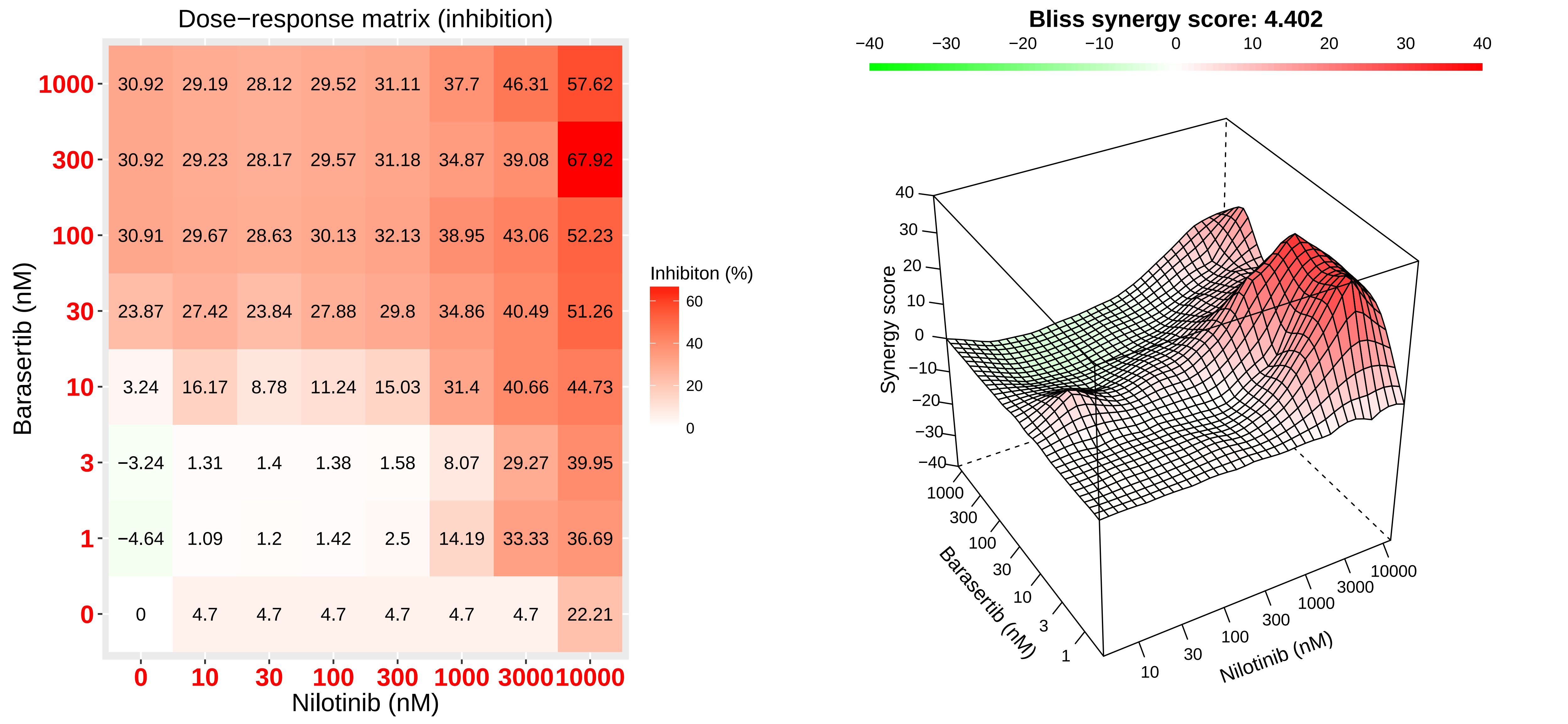
Dose−response matrix (inhibition)
Barasertib (nM)
Nilotinib (nM)
0 10 30 100 300 1000 3000 10000
1000: 30.92 29.19 28.12 29.52 31.11 37.7 46.31 57.62
300: 30.92 29.23 28.17 29.57 31.18 34.87 39.08 67.92
100: 30.91 29.67 28.63 30.13 32.13 38.95 43.06 52.23
30: 23.87 27.42 23.84 27.88 29.8 34.86 40.49 51.26
10: 3.24 16.17 8.78 11.24 15.03 31.4 40.66 44.73
3: −3.24 1.31 1.4 1.38 1.58 8.07 29.27 39.95
1: −4.64 1.09 1.2 1.42 2.5 14.19 33.33 36.69
0: 0 4.7 4.7 4.7 4.7 4.7 4.7 22.21
Inhibiton (%)
60
40
20
0
Bliss synergy score: 4.402
−40 −30 −20 −10 0 10 20 30 40
Synergy score
40 30 20 10 0 −10 −20 −30 −40
Barasertib (nM)
1000 300 100 30 10 3 1
Nilotinib (nM)
10 30 100 300 1000 3000 10000

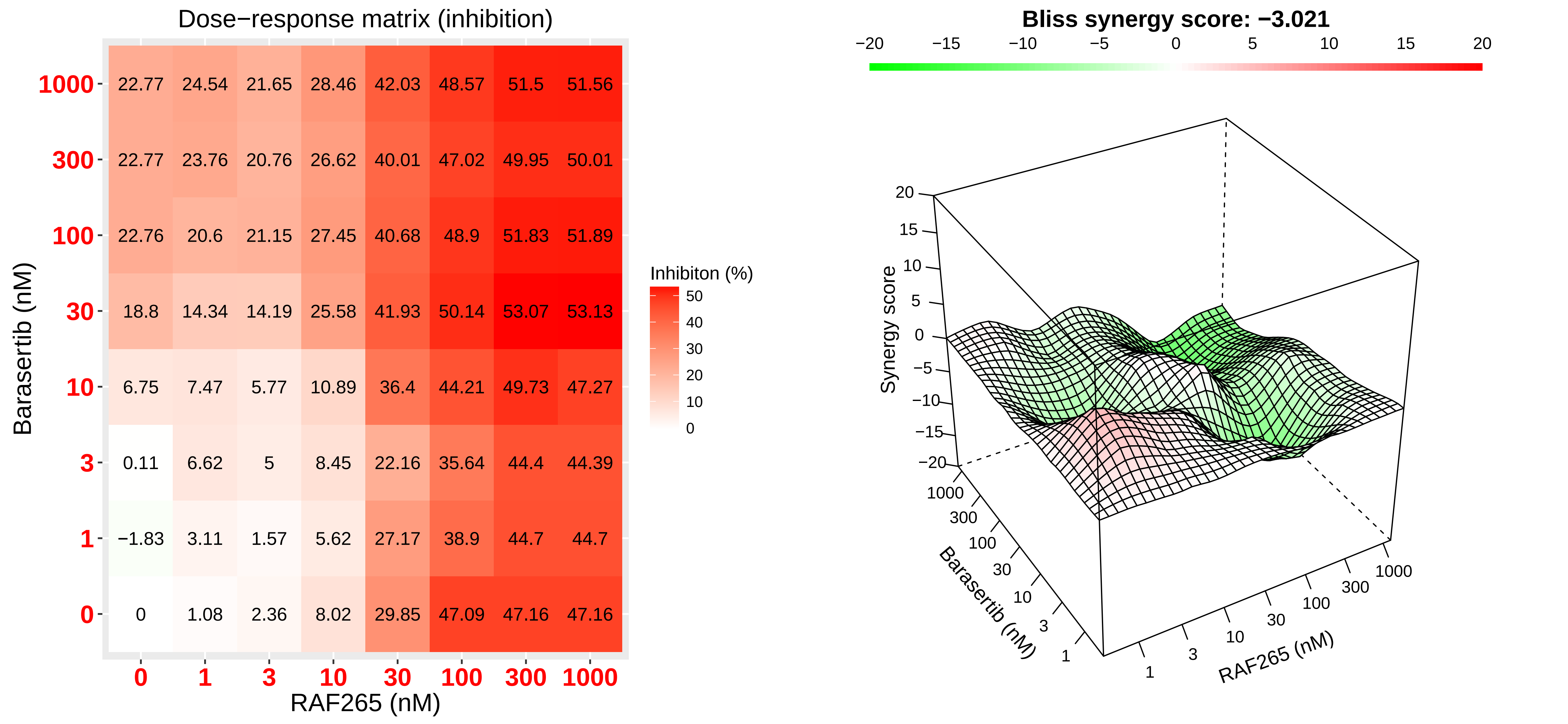

Dose−response matrix (inhibition)
1000 22.77 24.54 21.65 28.46 42.03 48.57 51.5 51.56
300 22.77 23.76 20.76 26.62 40.01 47.02 49.95 50.01
100 22.76 20.6 21.15 27.45 40.68 48.9 51.83 51.89
30 18.8 14.34 14.19 25.58 41.93 50.14 53.07 53.13
10 6.75 7.47 5.77 10.89 36.4 44.21 49.73 47.27
3 0.11 6.62 5 8.45 22.16 35.64 44.4 44.39
1 −1.83 3.11 1.57 5.62 27.17 38.9 44.7 44.7
0 0 1.08 2.36 8.02 29.85 47.09 47.16 47.16
0 1 3 10 30 100 300 1000
RAF265 (nM)
Barasertib (nM)
Inhibiton (%)
50
40
30
20
10
0
Bliss synergy score: −3.021
−20 −15 −10 −5 0 5 10 15 20
Synergy score
20
15
10
5
0
−5
−10
−15
−20
1000 300 100 30 10 3 1
Barasertib (nM)
1 3 10 30 100 300 1000
RAF265 (nM)

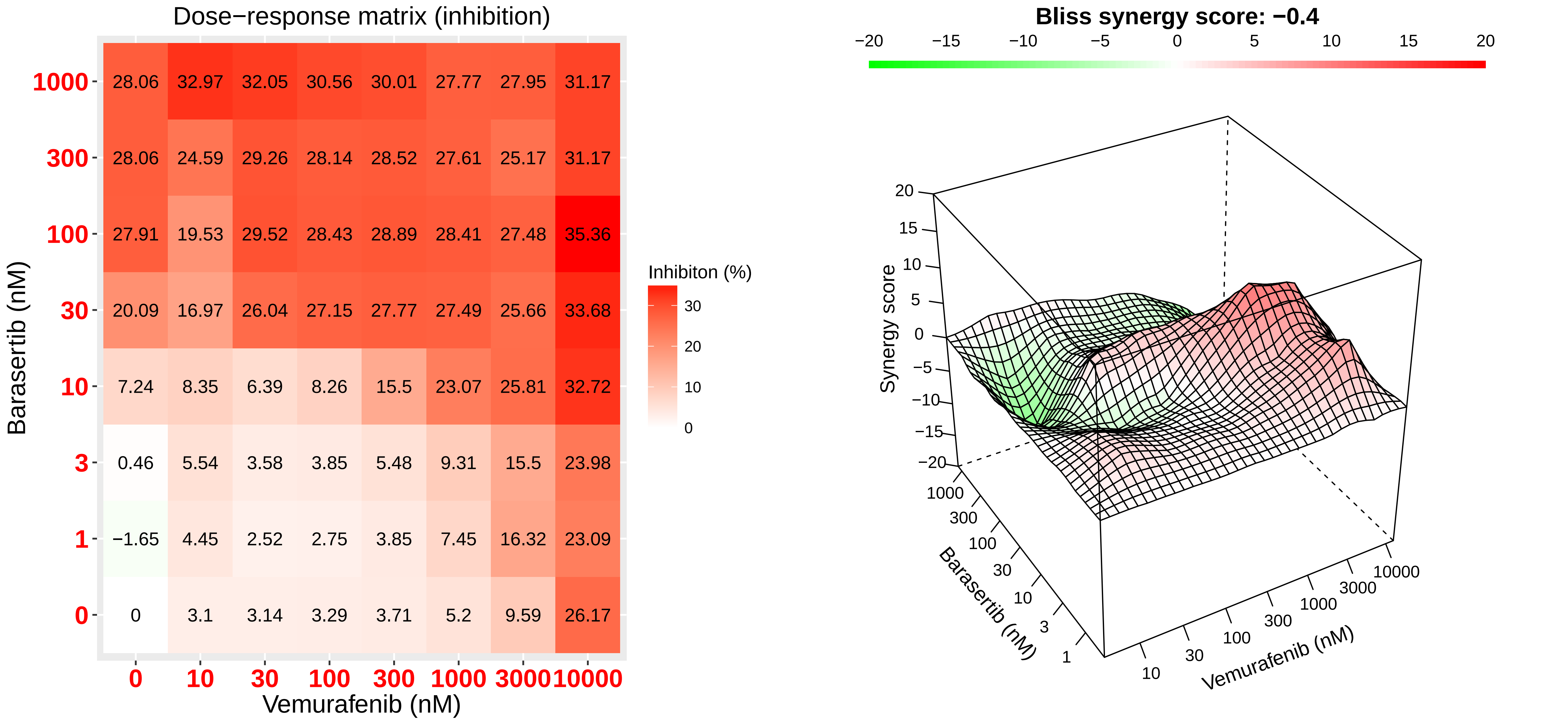

Dose−response matrix (inhibition)
1000
28.06 32.97 32.05 30.56 30.01 27.77 27.95 31.17
300
28.06 24.59 29.26 28.14 28.52 27.61 25.17 31.17
100
27.91 19.53 29.52 28.43 28.89 28.41 27.48 35.36
30
20.09 16.97 26.04 27.15 27.77 27.49 25.66 33.68
10
7.24 8.35 6.39 8.26 15.5 23.07 25.81 32.72
3
0.46 5.54 3.58 3.85 5.48 9.31 15.5 23.98
1
−1.65 4.45 2.52 2.75 3.85 7.45 16.32 23.09
0
0 3.1 3.14 3.29 3.71 5.2 9.59 26.17
Barasertib (nM)
0 10 30 100 300 1000 3000 10000
Vemurafenib (nM)
Inhibiton (%)
30
20
10
0
Bliss synergy score: −0.4
−20 −15 −10 −5 0 5 10 15 20
Synergy score
20 15 10 5 0 −5 −10 −15 −20
1000 300 100 30 10 3 1
Barasertib (nM)
10 30 100 300 1000 3000 10000
Vemurafenib (nM)

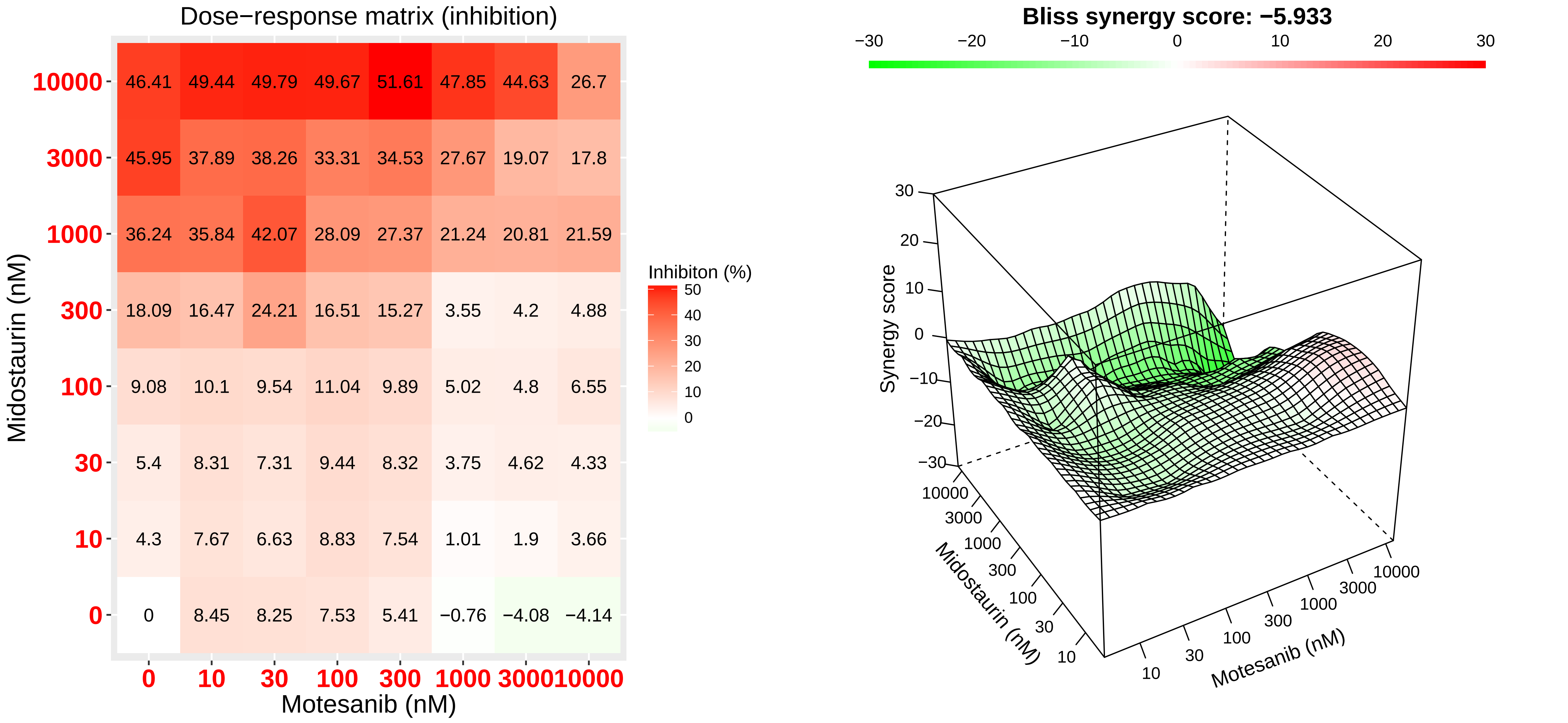
Dose−response matrix (inhibition)
10000 46.41 49.44 49.79 49.67 51.61 47.85 44.63 26.7
3000 45.95 37.89 38.26 33.31 34.53 27.67 19.07 17.8
1000 36.24 35.84 42.07 28.09 27.37 21.24 20.81 21.59
300 18.09 16.47 24.21 16.51 15.27 3.55 4.2 4.88
100 9.08 10.1 9.54 11.04 9.89 5.02 4.8 6.55
30 5.4 8.31 7.31 9.44 8.32 3.75 4.62 4.33
10 4.3 7.67 6.63 8.83 7.54 1.01 1.9 3.66
0 0 8.45 8.25 7.53 5.41 −0.76 −4.08 −4.14
0 10 30 100 300 1000 3000 10000
Midostaurin (nM)
Motesanib (nM)
Inhibiton (%)
50 40 30 20 10 0
Bliss synergy score: −5.933
−30 −20 −10 0 10 20 30
Synergy score
30 20 10 0 −10 −20 −30
10000 3000 1000 300 100 30 10
Midostaurin (nM)
10 30 100 300 1000 3000 10000
Motesanib (nM)

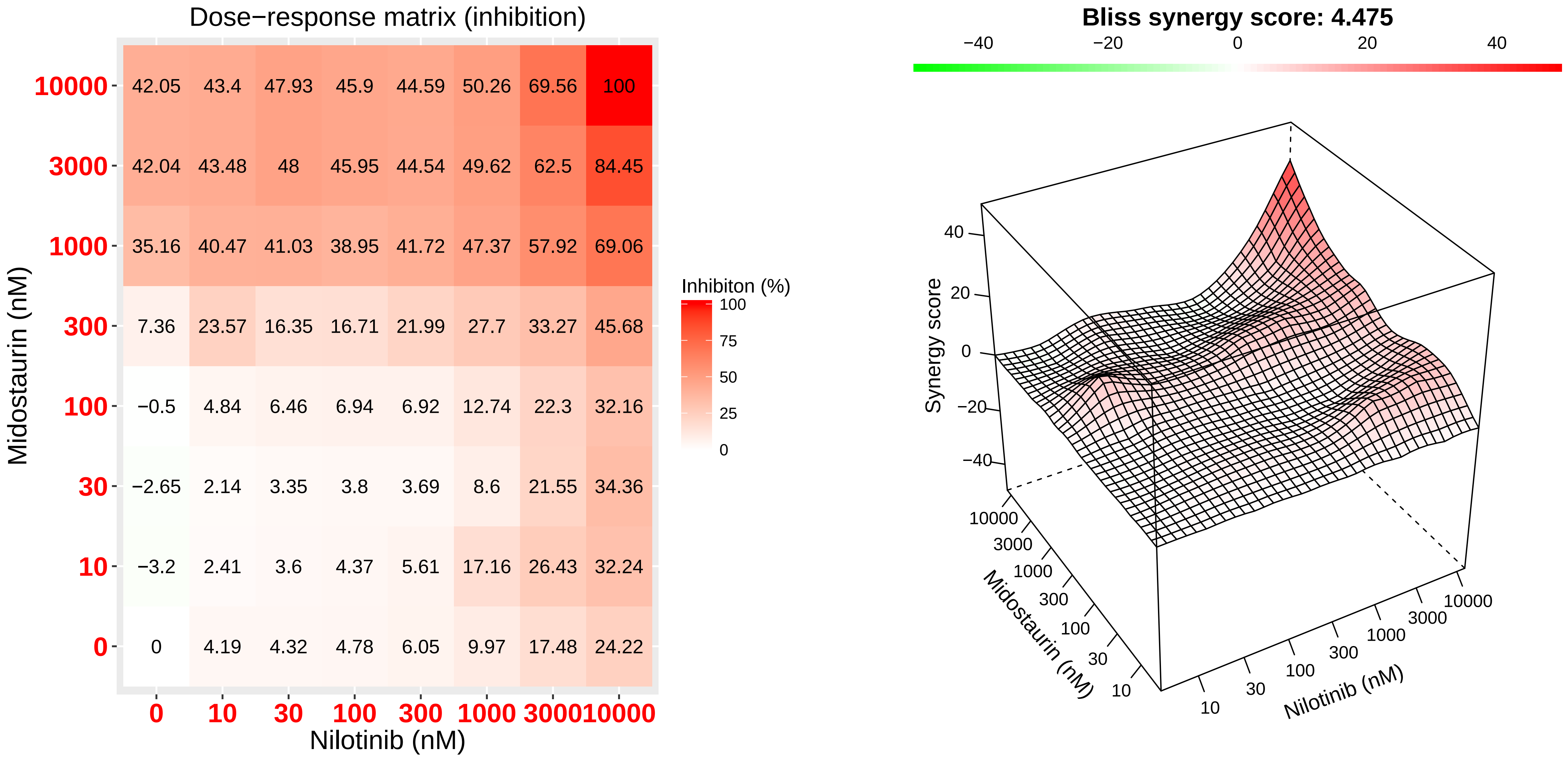
Dose−response matrix (inhibition)
Midostaurin (nM)
10000 42.05 43.4 47.93 45.9 44.59 50.26 69.56 100
3000 42.04 43.48 48 45.95 44.54 49.62 62.5 84.45
1000 35.16 40.47 41.03 38.95 41.72 47.37 57.92 69.06
300 7.36 23.57 16.35 16.71 21.99 27.7 33.27 45.68
100 −0.5 4.84 6.46 6.94 6.92 12.74 22.3 32.16
30 −2.65 2.14 3.35 3.8 3.69 8.6 21.55 34.36
10 −3.2 2.41 3.6 4.37 5.61 17.16 26.43 32.24
0 0 4.19 4.32 4.78 6.05 9.97 17.48 24.22
0 10 30 100 300 1000 3000 10000
Nilotinib (nM)
Inhibiton (%)
100
75
50
25
0
Bliss synergy score: 4.475
−40 −20 0 20 40
Synergy score
40
20
0
−20
−40
10000
3000
1000
300
100
30
10
Midostaurin (nM)
10 30 100 300 1000 3000 10000
Nilotinib (nM)

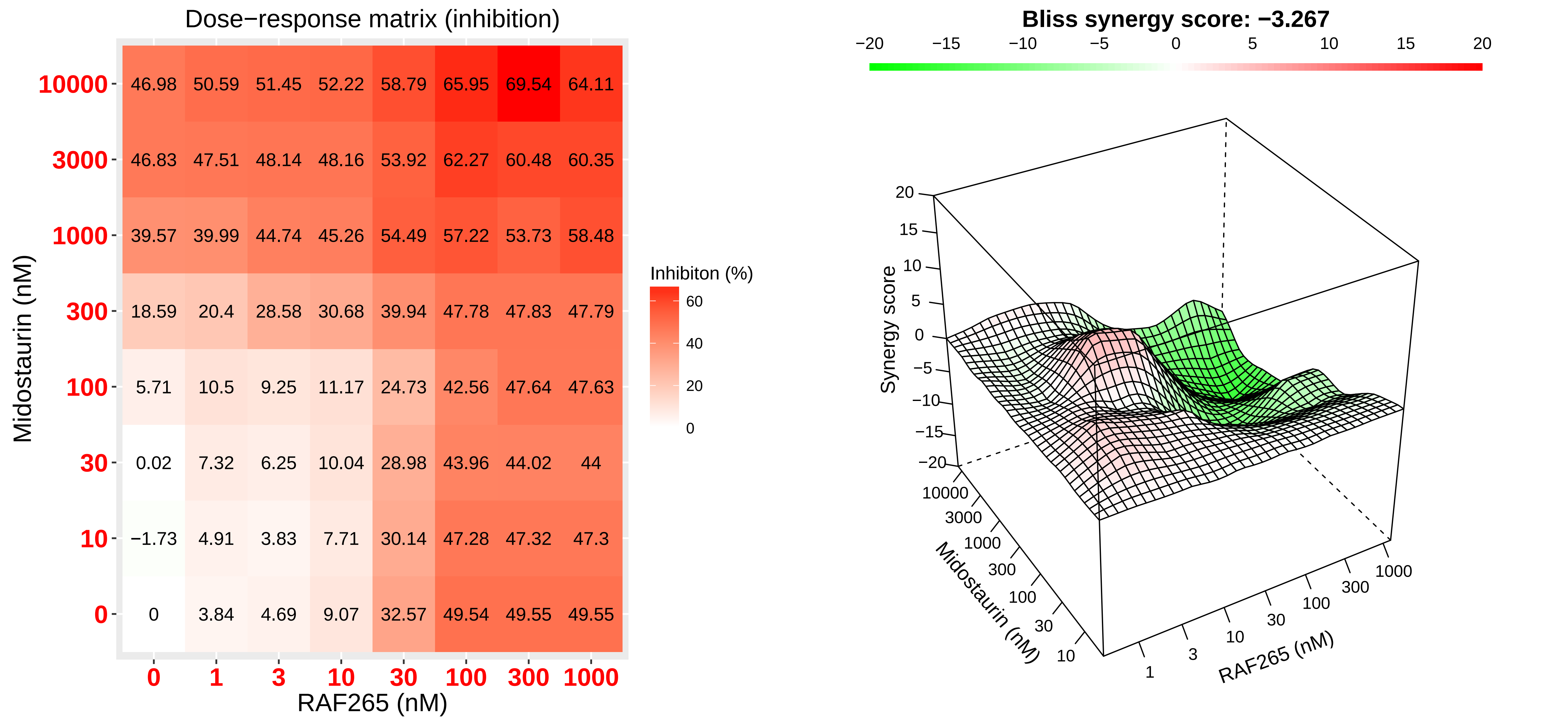
Dose−response matrix (inhibition)
10000
3000
1000
300
100
30
10
0
46.98 50.59 51.45 52.22 58.79 65.95 69.54 64.11
46.83 47.51 48.14 48.16 53.92 62.27 60.48 60.35
39.57 39.99 44.74 45.26 54.49 57.22 53.73 58.48
18.59 20.4 28.58 30.68 39.94 47.78 47.83 47.79
5.71 10.5 9.25 11.17 24.73 42.56 47.64 47.63
0.02 7.32 6.25 10.04 28.98 43.96 44.02 44
−1.73 4.91 3.83 7.71 30.14 47.28 47.32 47.3
0 3.84 4.69 9.07 32.57 49.54 49.55 49.55
0 1 3 10 30 100 300 1000
RAF265 (nM)
Midostaurin (nM)
Inhibiton (%)
60
40
20
0
Bliss synergy score: −3.267
−20 −15 −10 −5 0 5 10 15 20
Synergy score
20
15
10
5
0
−5
−10
−15
−20
10000
3000
1000
300
100
30
10
Midostaurin (nM)
1
3
10
30
100
300
1000
RAF265 (nM)

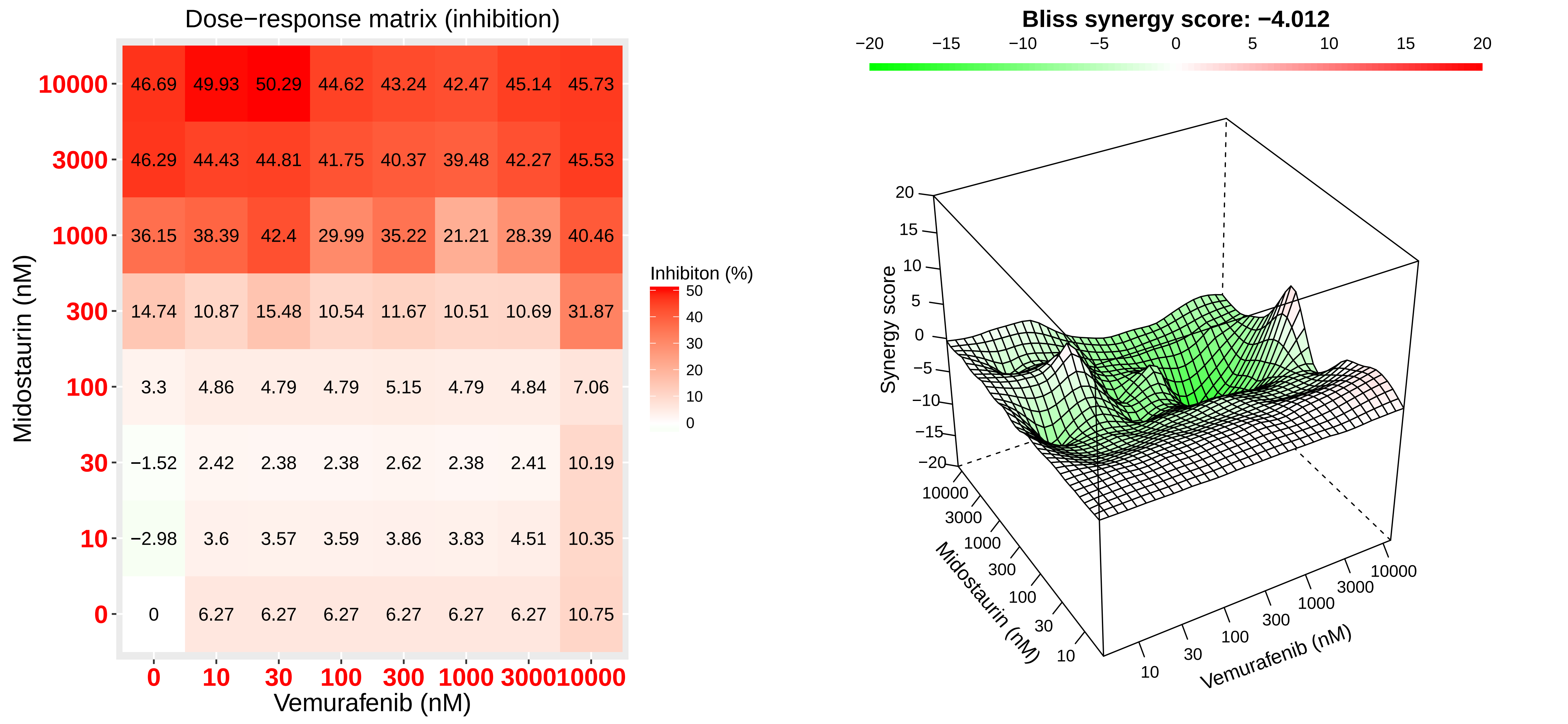

Dose−response matrix (inhibition)
10000
46.69 49.93 50.29 44.62 43.24 42.47 45.14 45.73
3000
46.29 44.43 44.81 41.75 40.37 39.48 42.27 45.53
1000
36.15 38.39 42.4 29.99 35.22 21.21 28.39 40.46
300
14.74 10.87 15.48 10.54 11.67 10.51 10.69 31.87
100
3.3 4.86 4.79 4.79 5.15 4.79 4.84 7.06
30
−1.52 2.42 2.38 2.38 2.62 2.38 2.41 10.19
10
−2.98 3.6 3.57 3.59 3.86 3.83 4.51 10.35
0
0 6.27 6.27 6.27 6.27 6.27 6.27 10.75
Midostaurin (nM)
0 10 30 100 300 1000 3000 10000
Vemurafenib (nM)
Inhibiton (%)
50 40 30 20 10 0
Bliss synergy score: −4.012
−20 −15 −10 −5 0 5 10 15 20
Synergy score
20 15 10 5 0 −5 −10 −15 −20
10000 3000 1000 300 100 30 10
Midostaurin (nM)
10 30 100 300 1000 3000 10000
Vemurafenib (nM)

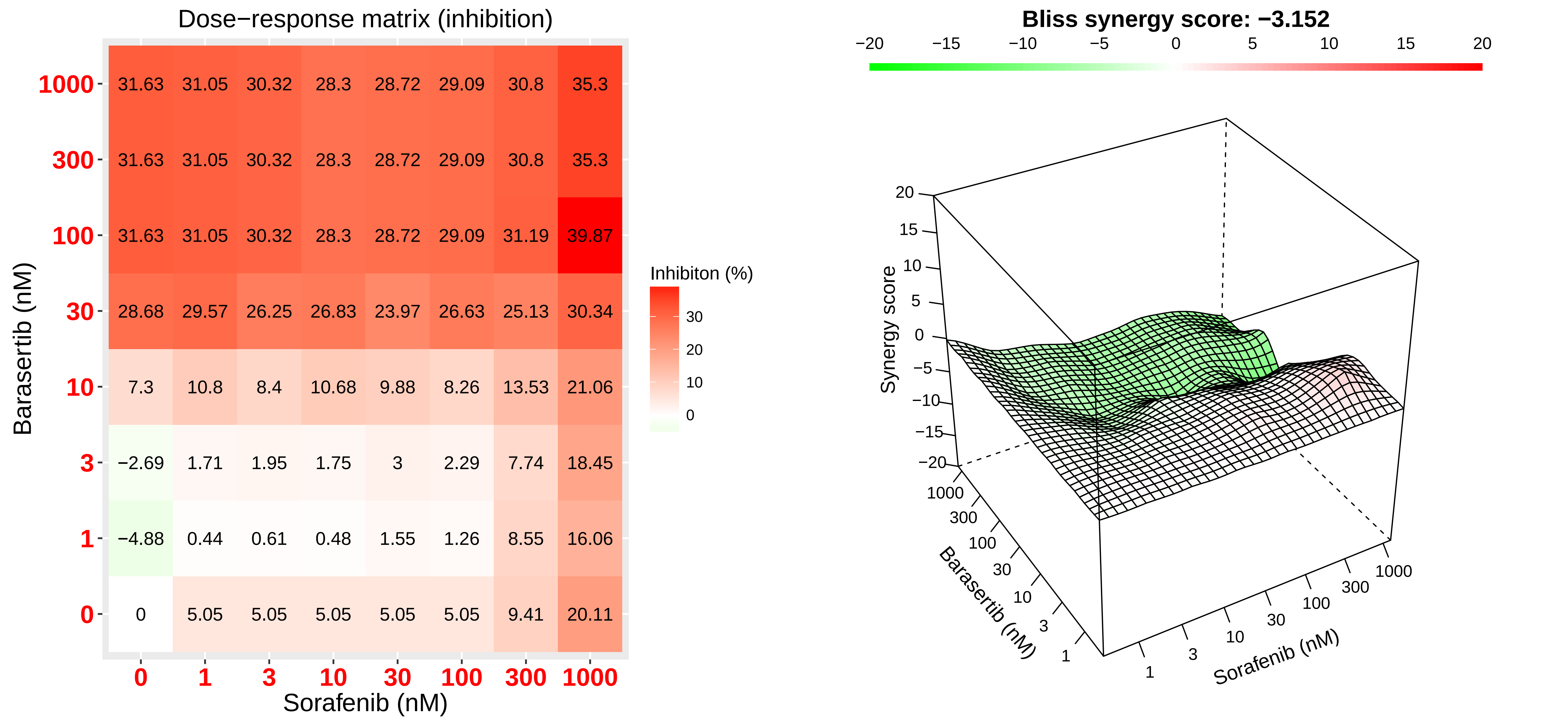

Dose−response matrix (inhibition)
1000 31.63 31.05 30.32 28.3 28.72 29.09 30.8 35.3
300 31.63 31.05 30.32 28.3 28.72 29.09 30.8 35.3
100 31.63 31.05 30.32 28.3 28.72 29.09 31.19 39.87
30 28.68 29.57 26.25 26.83 23.97 26.63 25.13 30.34
10 7.3 10.8 8.4 10.68 9.88 8.26 13.53 21.06
3 −2.69 1.71 1.95 1.75 3 2.29 7.74 18.45
1 −4.88 0.44 0.61 0.48 1.55 1.26 8.55 16.06
0 0 5.05 5.05 5.05 5.05 5.05 9.41 20.11
0 1 3 10 30 100 300 1000
Sorafenib (nM)
Barasertib (nM)
Inhibiton (%)
30
20
10
0
Bliss synergy score: −3.152
−20 −15 −10 −5 0 5 10 15 20
Synergy score
20 15 10 5 0 −5 −10 −15 −20
Barasertib (nM)
1000 300 100 30 10 3 1
Sorafenib (nM)
1 3 10 30 100 300 1000

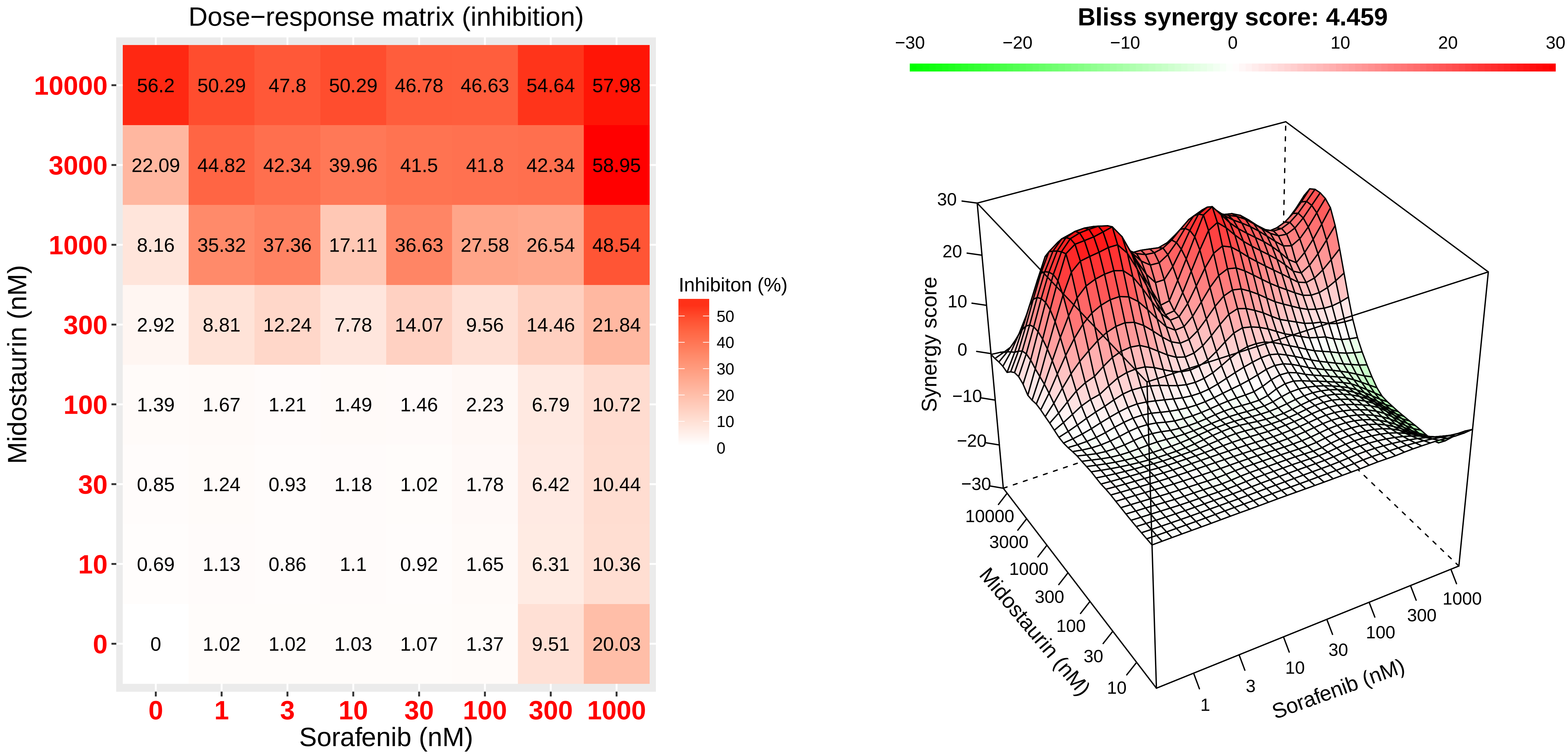
Dose−response matrix (inhibition)
10000 56.2 50.29 47.8 50.29 46.78 46.63 54.64 57.98
3000 22.09 44.82 42.34 39.96 41.5 41.8 42.34 58.95
1000 8.16 35.32 37.36 17.11 36.63 27.58 26.54 48.54
300 2.92 8.81 12.24 7.78 14.07 9.56 14.46 21.84
100 1.39 1.67 1.21 1.49 1.46 2.23 6.79 10.72
30 0.85 1.24 0.93 1.18 1.02 1.78 6.42 10.44
10 0.69 1.13 0.86 1.1 0.92 1.65 6.31 10.36
0 0 1.02 1.02 1.03 1.07 1.37 9.51 20.03
0 1 3 10 30 100 300 1000
Sorafenib (nM)
Midostaurin (nM)
Inhibiton (%)
50
40
30
20
10
0
Bliss synergy score: 4.459
−30 −20 −10 0 10 20 30
Synergy score
30 20 10 0 −10 −20 −30
Midostaurin (nM)
10000 3000 1000 300 100 30 10
Sorafenib (nM)
1 3 10 30 100 300 1000

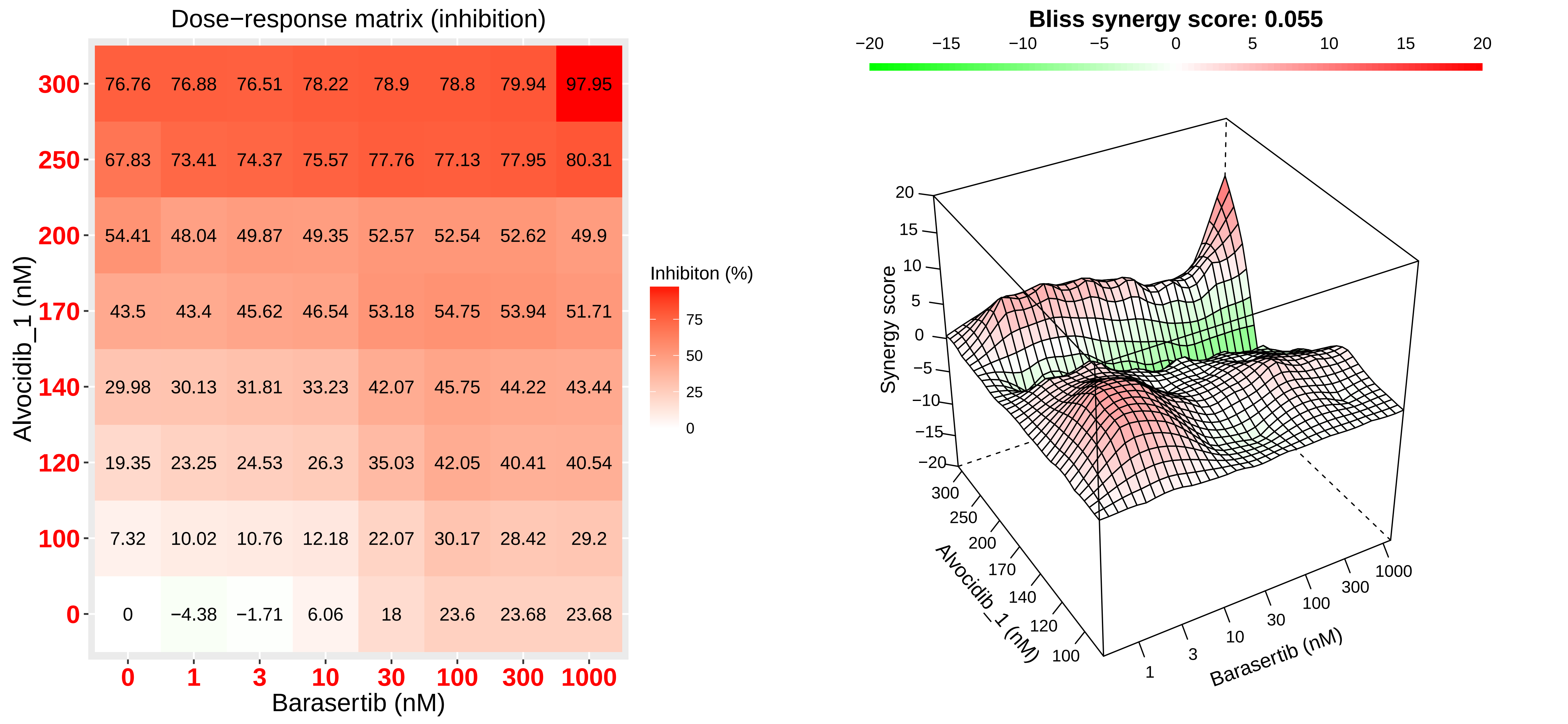

Dose−response matrix (inhibition)
Alvocidib_1 (nM)
300
250
200
170
140
120
100
0
76.76 76.88 76.51 78.22 78.9 78.8 79.94 97.95
67.83 73.41 74.37 75.57 77.76 77.13 77.95 80.31
54.41 48.04 49.87 49.35 52.57 52.54 52.62 49.9
43.5 43.4 45.62 46.54 53.18 54.75 53.94 51.71
29.98 30.13 31.81 33.23 42.07 45.75 44.22 43.44
19.35 23.25 24.53 26.3 35.03 42.05 40.41 40.54
7.32 10.02 10.76 12.18 22.07 30.17 28.42 29.2
0 −4.38 −1.71 6.06 18 23.6 23.68 23.68
0 1 3 10 30 100 300 1000
Barasertib (nM)
Inhibiton (%)
75
50
25
0
Bliss synergy score: 0.055
−20 −15 −10 −5 0 5 10 15 20
Synergy score
20 15 10 5 0 −5 −10 −15 −20
Alvocidib_1 (nM)
300 250 200 170 140 120 100
Barasertib (nM)
1 3 10 30 100 300 1000

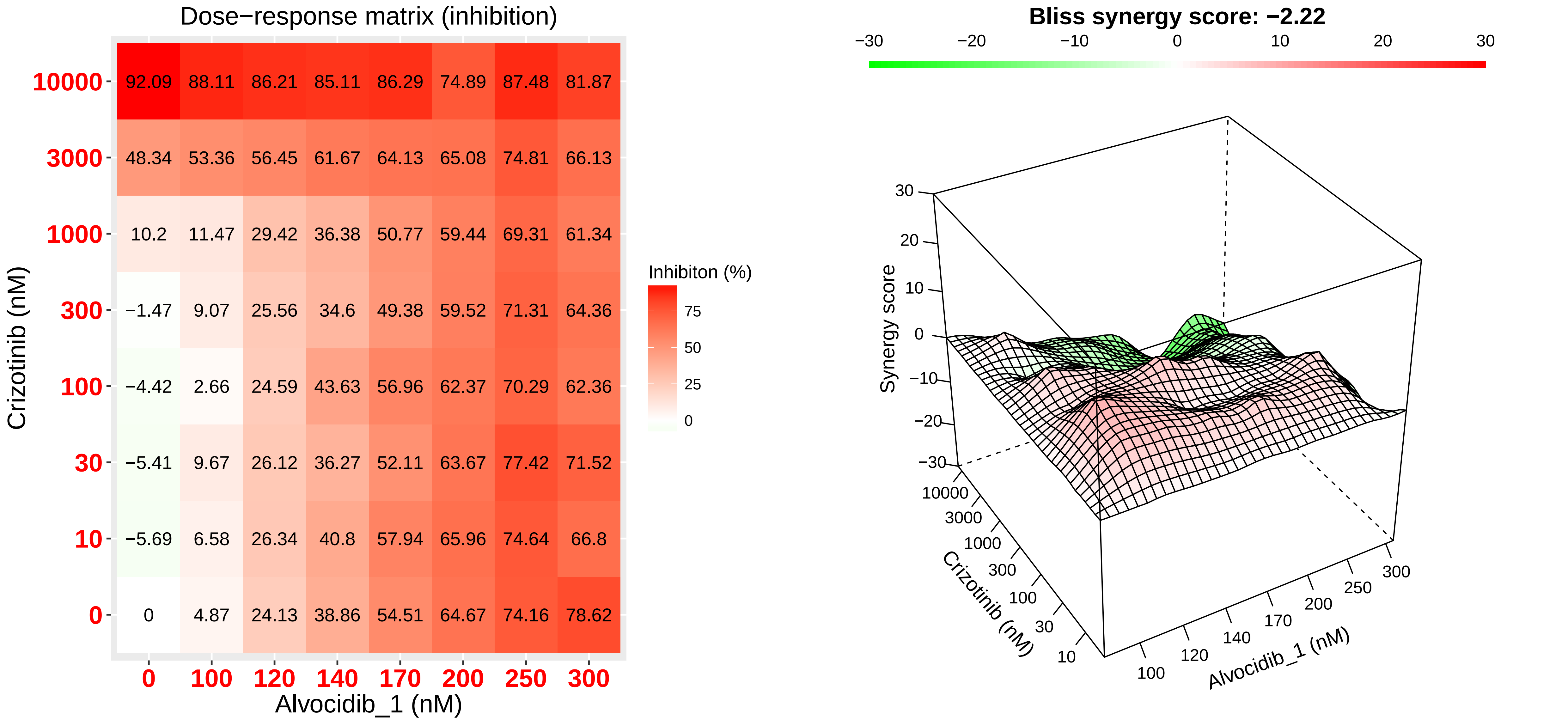

Dose−response matrix (inhibition)
Crizotinib (nM)
10000 3000 1000 300 100 30 10 0
Alvocidib_1 (nM)
0 100 120 140 170 200 250 300
92.09 88.11 86.21 85.11 86.29 74.89 87.48 81.87
48.34 53.36 56.45 61.67 64.13 65.08 74.81 66.13
10.2 11.47 29.42 36.38 50.77 59.44 69.31 61.34
−1.47 9.07 25.56 34.6 49.38 59.52 71.31 64.36
−4.42 2.66 24.59 43.63 56.96 62.37 70.29 62.36
−5.41 9.67 26.12 36.27 52.11 63.67 77.42 71.52
−5.69 6.58 26.34 40.8 57.94 65.96 74.64 66.8
0 4.87 24.13 38.86 54.51 64.67 74.16 78.62
Inhibiton (%)
75
50
25
0
Bliss synergy score: −2.22
−30 −20 −10 0 10 20 30
Synergy score
30 20 10 0 −10 −20 −30
Crizotinib (nM)
10000 3000 1000 300 100 30 10
Alvocidib_1 (nM)
100 120 140 170 200 250 300

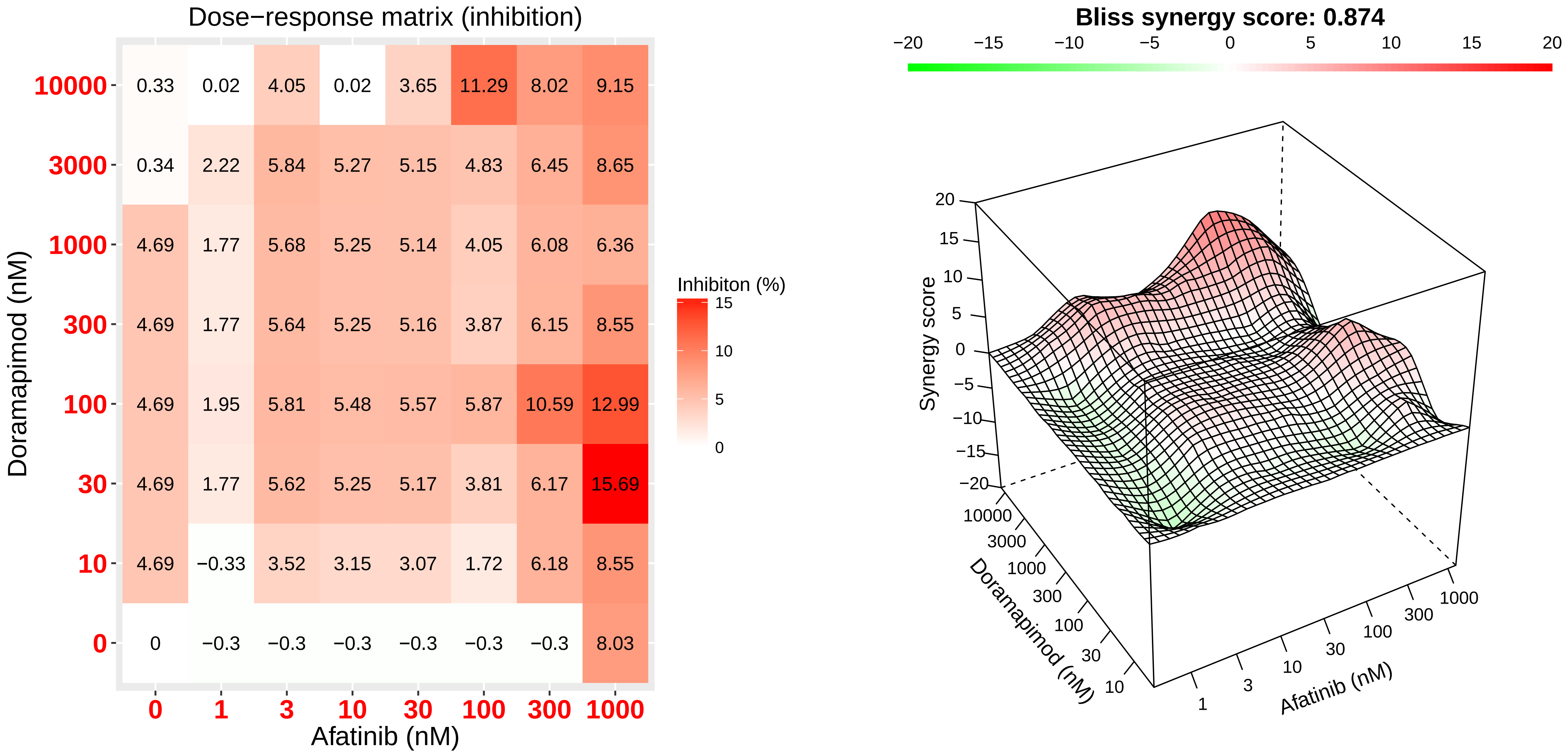
Dose−response matrix (inhibition)
Doramapimod (nM)
10000 0.33 0.02 4.05 0.02 3.65 11.29 8.02 9.15
3000 0.34 2.22 5.84 5.27 5.15 4.83 6.45 8.65
1000 4.69 1.77 5.68 5.25 5.14 4.05 6.08 6.36
300 4.69 1.77 5.64 5.25 5.16 3.87 6.15 8.55
100 4.69 1.95 5.81 5.48 5.57 5.87 10.59 12.99
30 4.69 1.77 5.62 5.25 5.17 3.81 6.17 15.69
10 4.69 −0.33 3.52 3.15 3.07 1.72 6.18 8.55
0 0 −0.3 −0.3 −0.3 −0.3 −0.3 −0.3 8.03
0 1 3 10 30 100 300 1000
Afatinib (nM)
Inhibiton (%)
15
10
5
0
Bliss synergy score: 0.874
−20 −15 −10 −5 0 5 10 15 20
Synergy score
20
15
10
5
0
−5
−10
−15
−20
10000
3000
1000
300
100
30
10
Doramapimod (nM)
1
3
10
30
100
300
1000
Afatinib (nM)

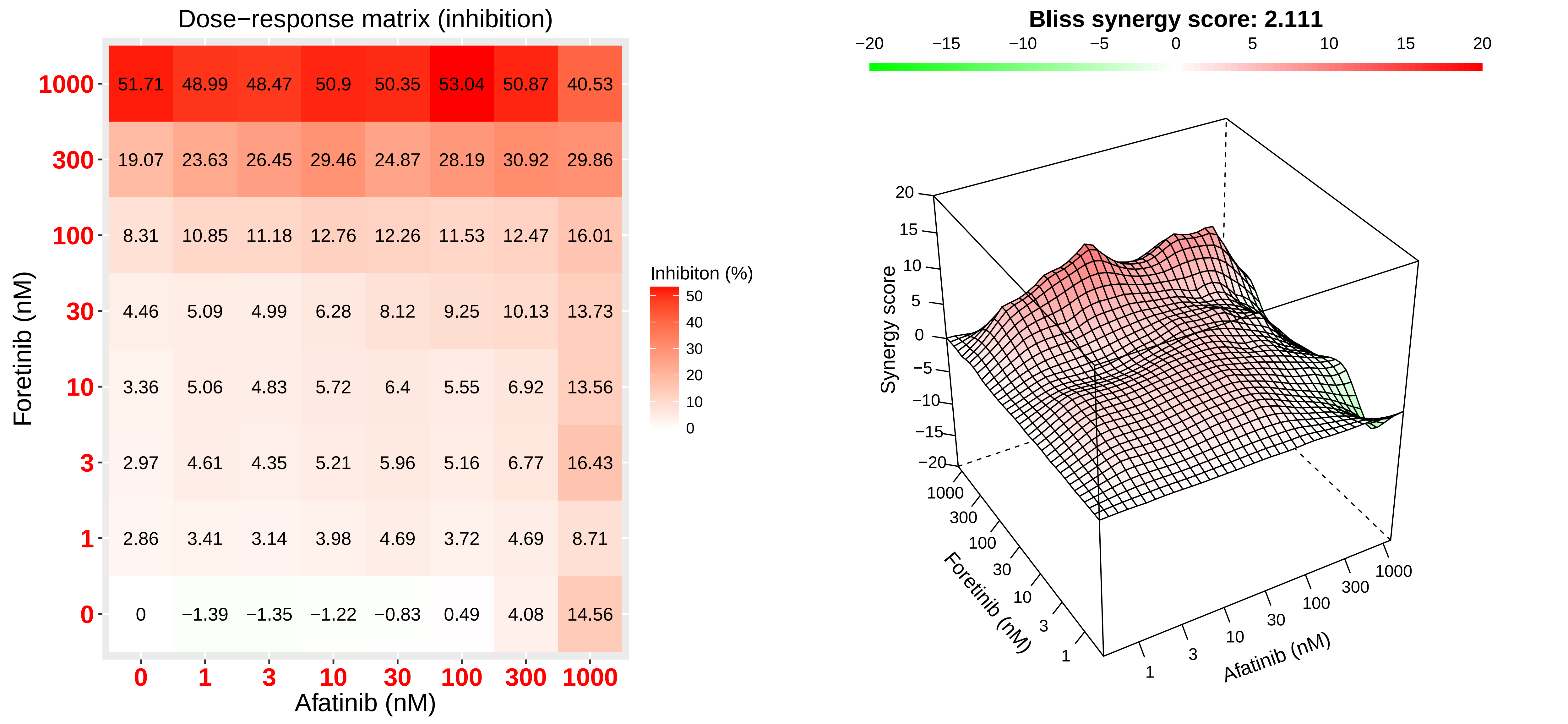
Dose−response matrix (inhibition)
1000 51.71 48.99 48.47 50.9 50.35 53.04 50.87 40.53
300 19.07 23.63 26.45 29.46 24.87 28.19 30.92 29.86
100 8.31 10.85 11.18 12.76 12.26 11.53 12.47 16.01
30 4.46 5.09 4.99 6.28 8.12 9.25 10.13 13.73
10 3.36 5.06 4.83 5.72 6.4 5.55 6.92 13.56
3 2.97 4.61 4.35 5.21 5.96 5.16 6.77 16.43
1 2.86 3.41 3.14 3.98 4.69 3.72 4.69 8.71
0 0 −1.39 −1.35 −1.22 −0.83 0.49 4.08 14.56
0 1 3 10 30 100 300 1000
Afatinib (nM)
Foretinib (nM)
Inhibiton (%)
50 40 30 20 10 0
Bliss synergy score: 2.111
−20 −15 −10 −5 0 5 10 15 20
Synergy score
20 15 10 5 0 −5 −10 −15 −20
1000 300 100 30 10 3 1
Foretinib (nM)
1 3 10 30 100 300 1000
Afatinib (nM)

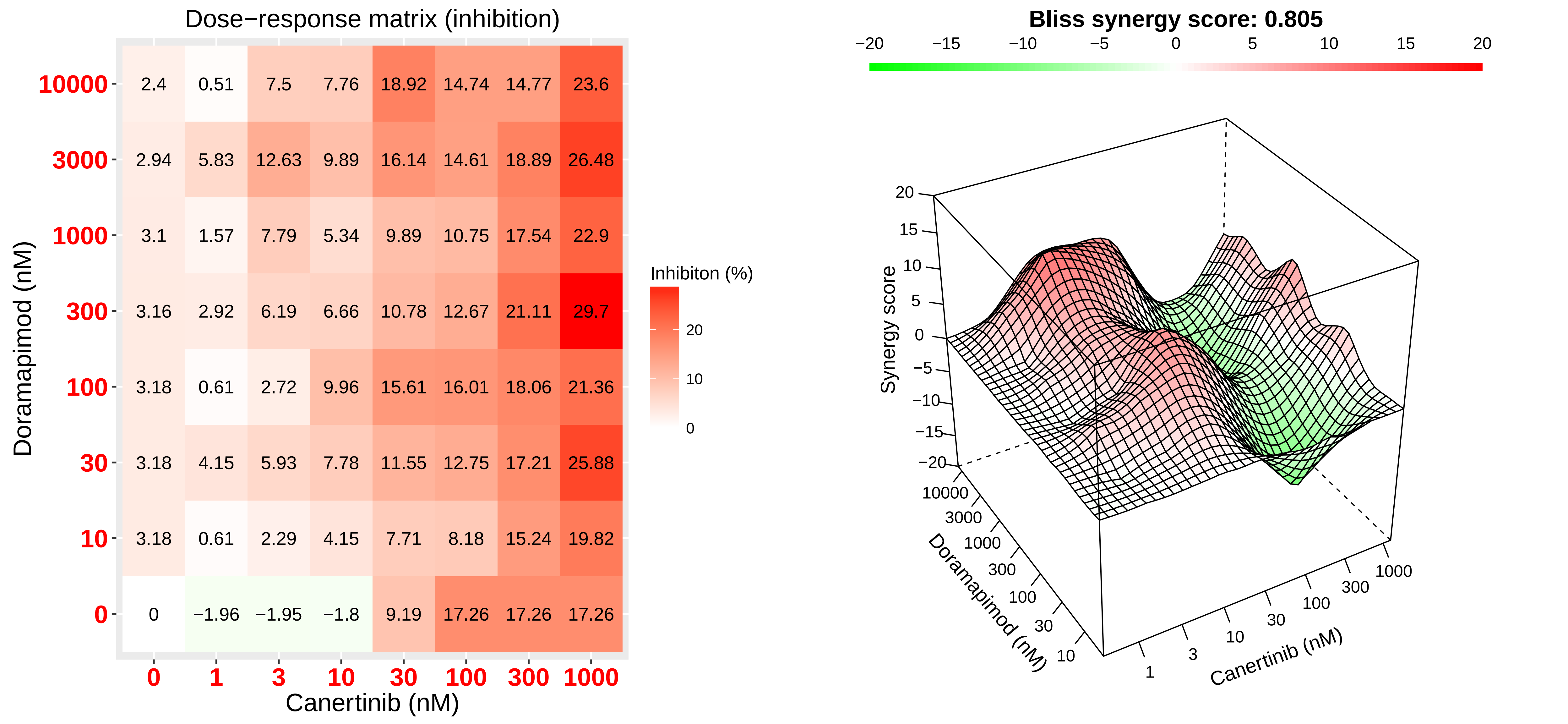
Dose−response matrix (inhibition)
10000 2.4 0.51 7.5 7.76 18.92 14.74 14.77 23.6
3000 2.94 5.83 12.63 9.89 16.14 14.61 18.89 26.48
1000 3.1 1.57 7.79 5.34 9.89 10.75 17.54 22.9
300 3.16 2.92 6.19 6.66 10.78 12.67 21.11 29.7
100 3.18 0.61 2.72 9.96 15.61 16.01 18.06 21.36
30 3.18 4.15 5.93 7.78 11.55 12.75 17.21 25.88
10 3.18 0.61 2.29 4.15 7.71 8.18 15.24 19.82
0 0 −1.96 −1.95 −1.8 9.19 17.26 17.26 17.26
0 1 3 10 30 100 300 1000
Canertinib (nM)
Doramapimod (nM)
Inhibiton (%)
20
10
0
Bliss synergy score: 0.805
−20 −15 −10 −5 0 5 10 15 20
Synergy score
20 15 10 5 0 −5 −10 −15 −20
10000 3000 1000 300 100 30 10
Doramapimod (nM)
1 3 10 30 100 300 1000
Canertinib (nM)

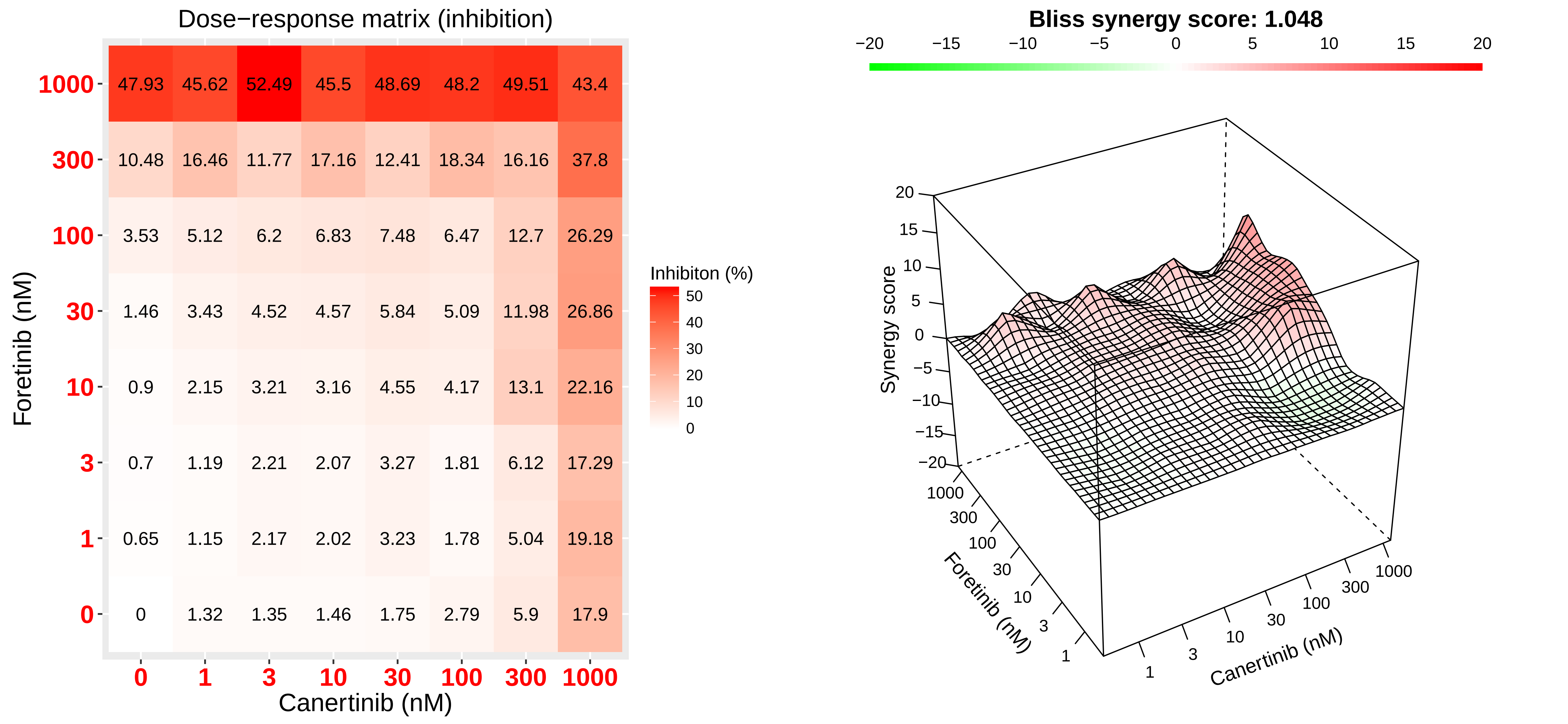
Dose−response matrix (inhibition)
1000 47.93 45.62 52.49 45.5 48.69 48.2 49.51 43.4
300 10.48 16.46 11.77 17.16 12.41 18.34 16.16 37.8
100 3.53 5.12 6.2 6.83 7.48 6.47 12.7 26.29
30 1.46 3.43 4.52 4.57 5.84 5.09 11.98 26.86
10 0.9 2.15 3.21 3.16 4.55 4.17 13.1 22.16
3 0.7 1.19 2.21 2.07 3.27 1.81 6.12 17.29
1 0.65 1.15 2.17 2.02 3.23 1.78 5.04 19.18
0 0 1.32 1.35 1.46 1.75 2.79 5.9 17.9
0 1 3 10 30 100 300 1000
Canertinib (nM)
Foretinib (nM)
Inhibiton (%)
50 40 30 20 10 0
Bliss synergy score: 1.048
−20 −15 −10 −5 0 5 10 15 20
Synergy score
20 15 10 5 0 −5 −10 −15 −20
Foretinib (nM)
1000 300 100 30 10 3 1
Canertinib (nM)
1 3 10 30 100 300 1000

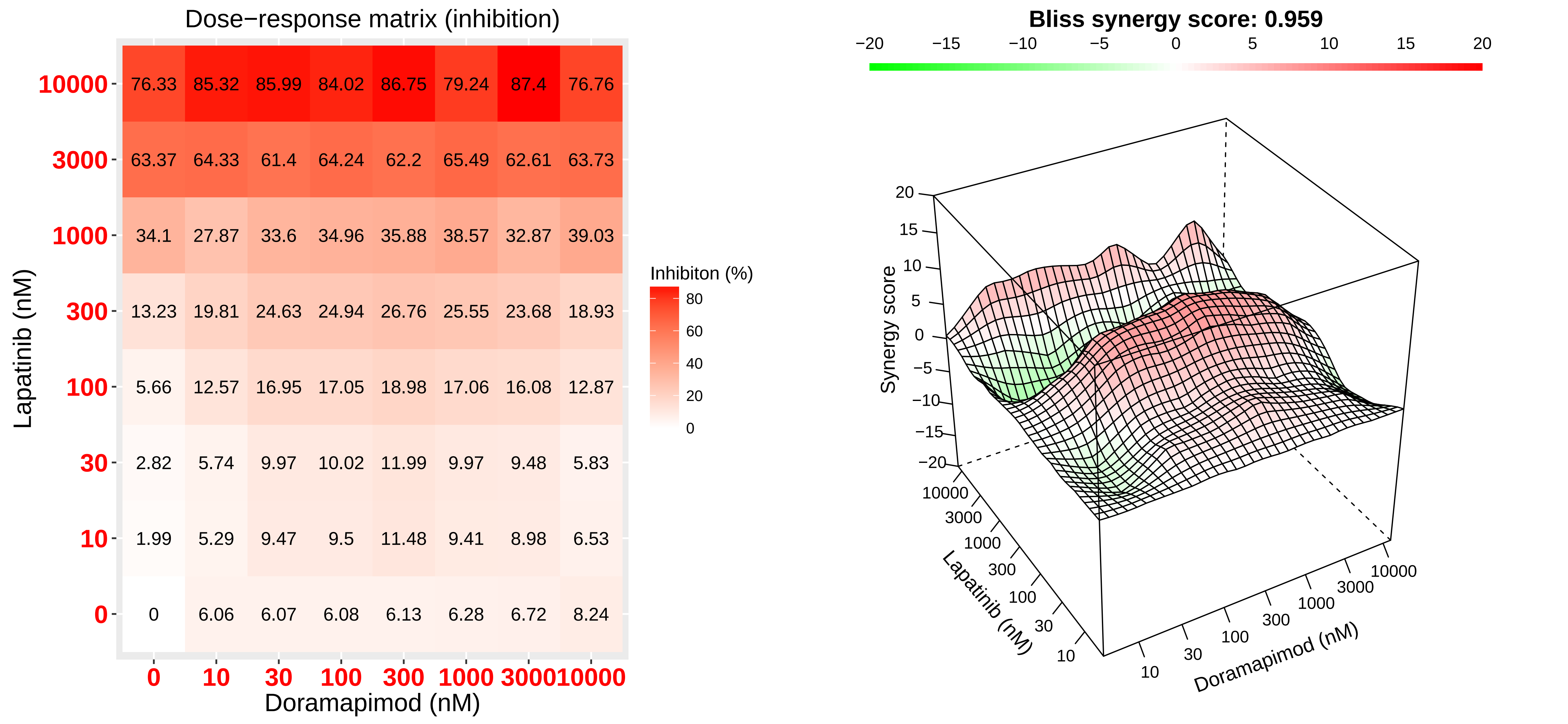

Dose−response matrix (inhibition)
10000 | 76.33 | 85.32 | 85.99 | 84.02 | 86.75 | 79.24 | 87.4 | 76.76
3000 | 63.37 | 64.33 | 61.4 | 64.24 | 62.2 | 65.49 | 62.61 | 63.73
1000 | 34.1 | 27.87 | 33.6 | 34.96 | 35.88 | 38.57 | 32.87 | 39.03
300 | 13.23 | 19.81 | 24.63 | 24.94 | 26.76 | 25.55 | 23.68 | 18.93
100 | 5.66 | 12.57 | 16.95 | 17.05 | 18.98 | 17.06 | 16.08 | 12.87
30 | 2.82 | 5.74 | 9.97 | 10.02 | 11.99 | 9.97 | 9.48 | 5.83
10 | 1.99 | 5.29 | 9.47 | 9.5 | 11.48 | 9.41 | 8.98 | 6.53
0 | 0 | 6.06 | 6.07 | 6.08 | 6.13 | 6.28 | 6.72 | 8.24
0 10 30 100 300 1000 3000 10000
Doramapimod (nM)
Lapatinib (nM)
Inhibiton (%)
80
60
40
20
0
Bliss synergy score: 0.959
−20 −15 −10 −5 0 5 10 15 20
Synergy score
20 15 10 5 0 −5 −10 −15 −20
10000 3000 1000 300 100 30 10
Lapatinib (nM)
10 30 100 300 1000 3000 10000
Doramapimod (nM)

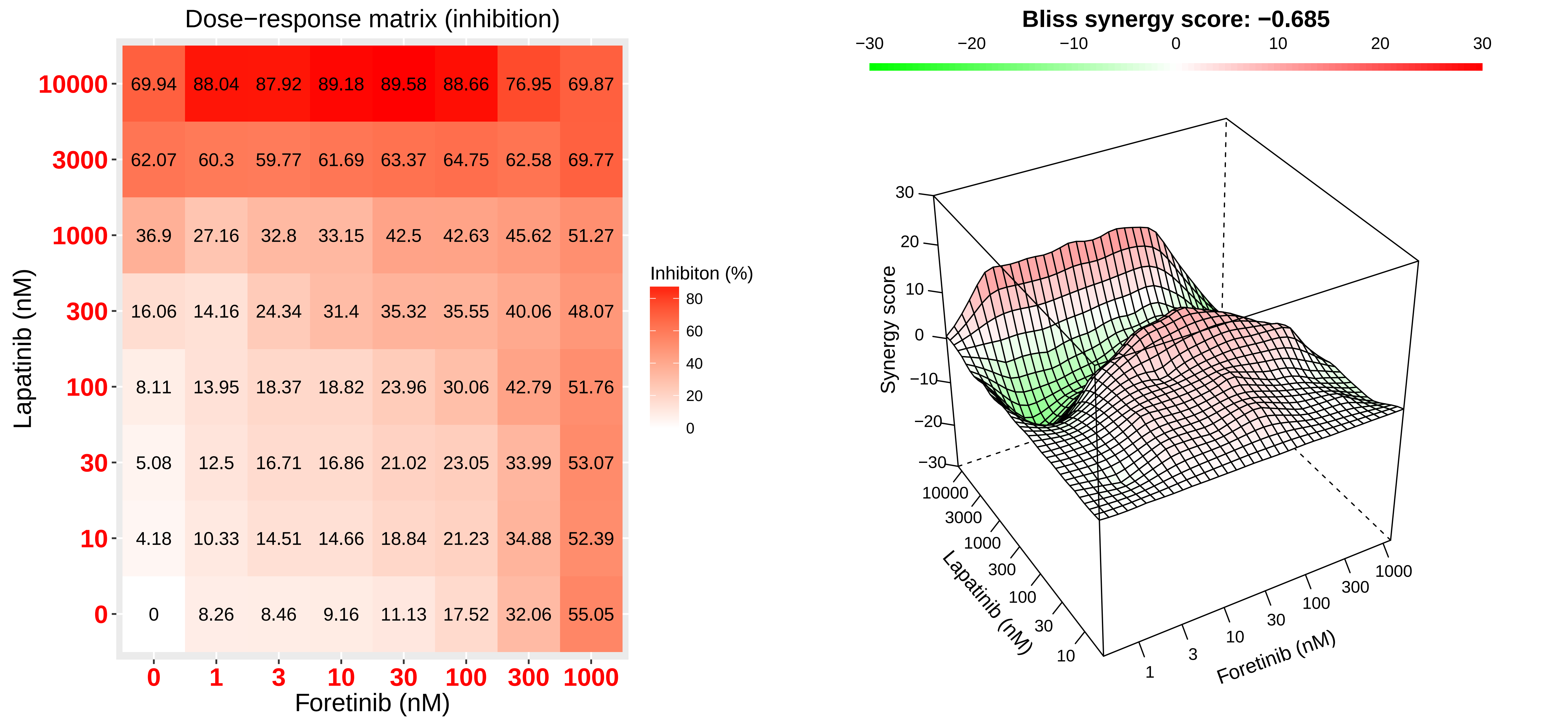

Dose−response matrix (inhibition)
69.94 88.04 87.92 89.18 89.58 88.66 76.95 69.87
62.07 60.3 59.77 61.69 63.37 64.75 62.58 69.77
36.9 27.16 32.8 33.15 42.5 42.63 45.62 51.27
16.06 14.16 24.34 31.4 35.32 35.55 40.06 48.07
8.11 13.95 18.37 18.82 23.96 30.06 42.79 51.76
5.08 12.5 16.71 16.86 21.02 23.05 33.99 53.07
4.18 10.33 14.51 14.66 18.84 21.23 34.88 52.39
0 8.26 8.46 9.16 11.13 17.52 32.06 55.05
10000
3000
1000
300
100
30
10
0
Lapatinib (nM)
0 1 3 10 30 100 300 1000
Foretinib (nM)
Inhibiton (%)
80
60
40
20
0
Bliss synergy score: −0.685
−30 −20 −10 0 10 20 30
Synergy score
30
20
10
0
−10
−20
−30
10000
3000
1000
300
100
30
10
Lapatinib (nM)
1
3
10
30
100
300
1000
Foretinib (nM)

Supplementary Figure 2

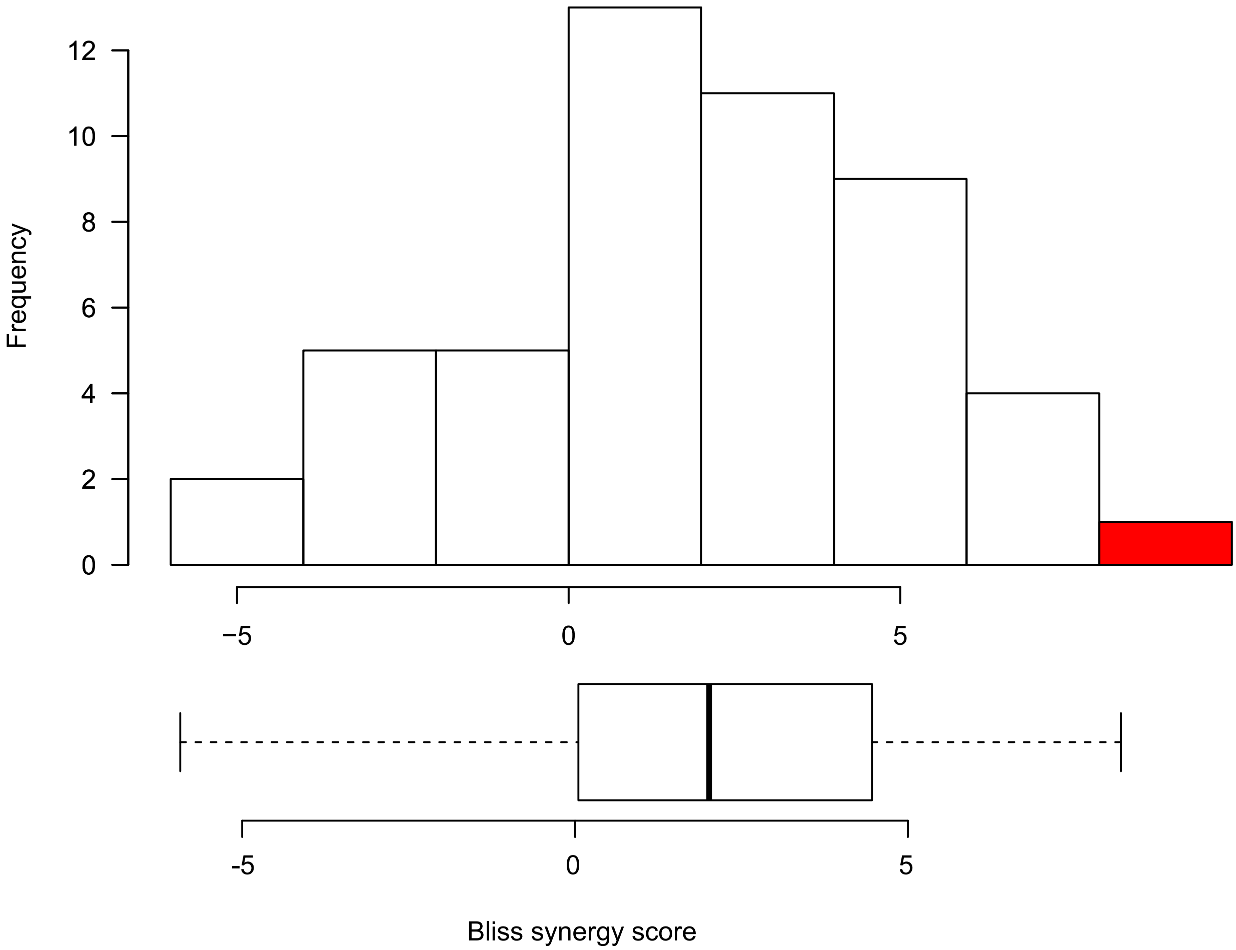

Supplementary Figure 3

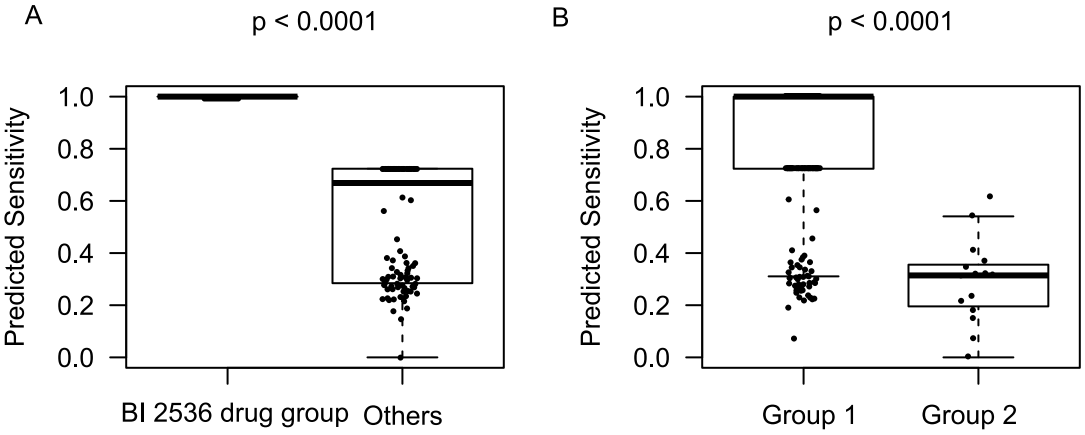

Supplementary Figure 4

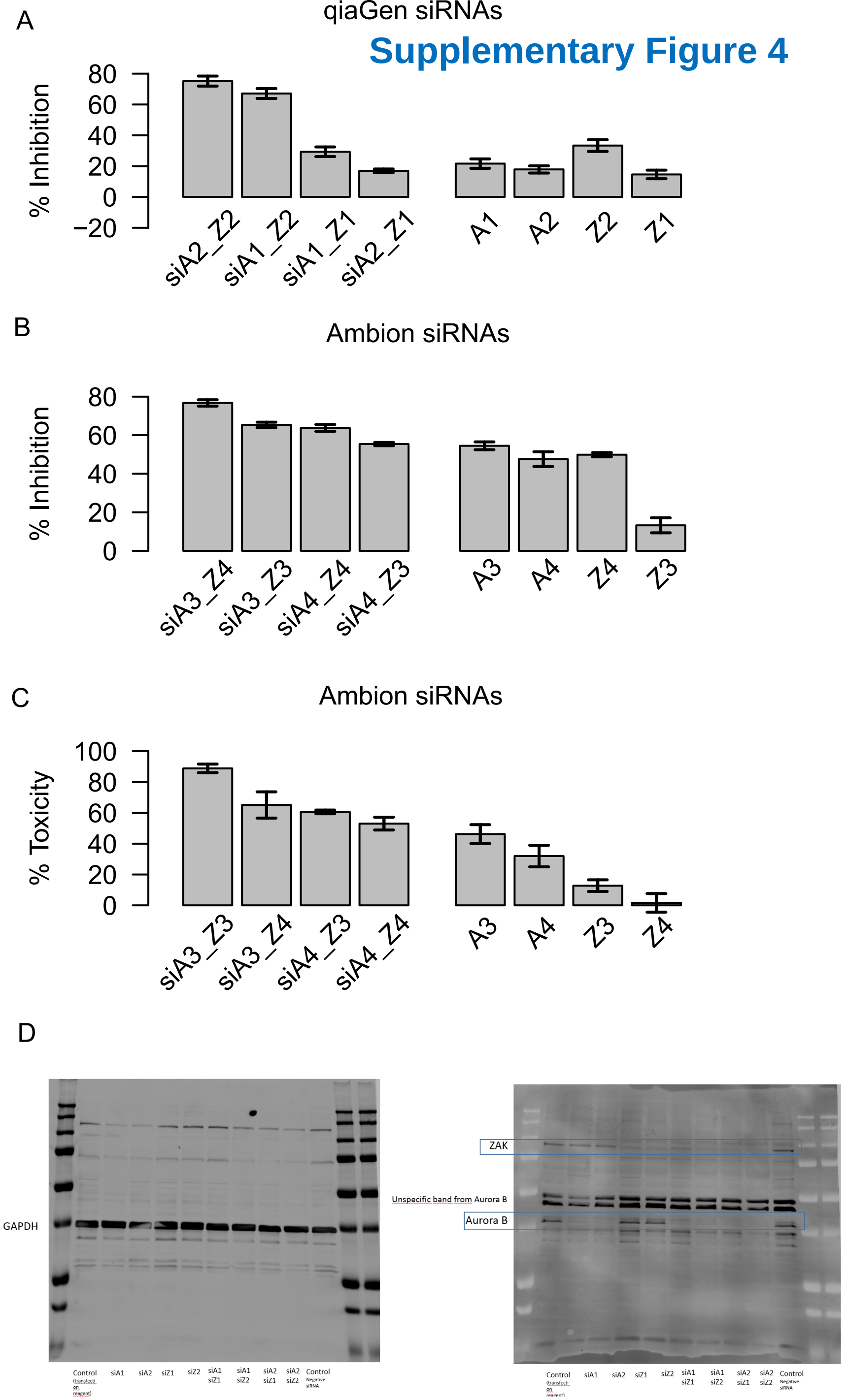

Supplementary Figure 5

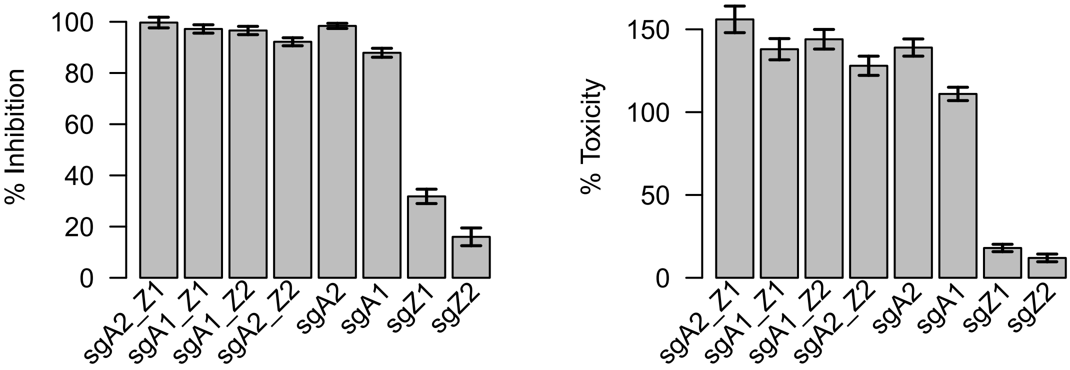

Supplementary Figure 6

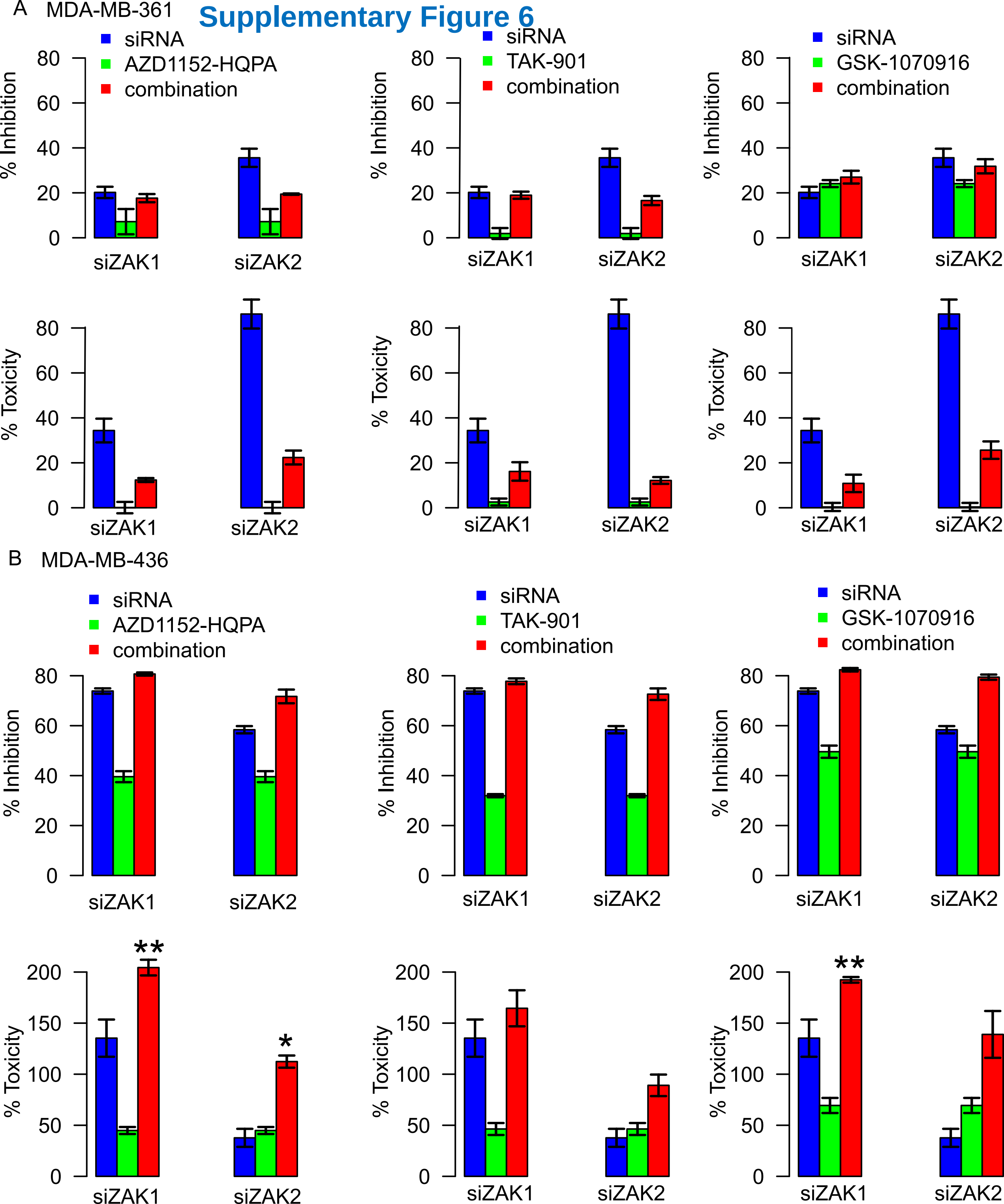

Supplementary Figure 7

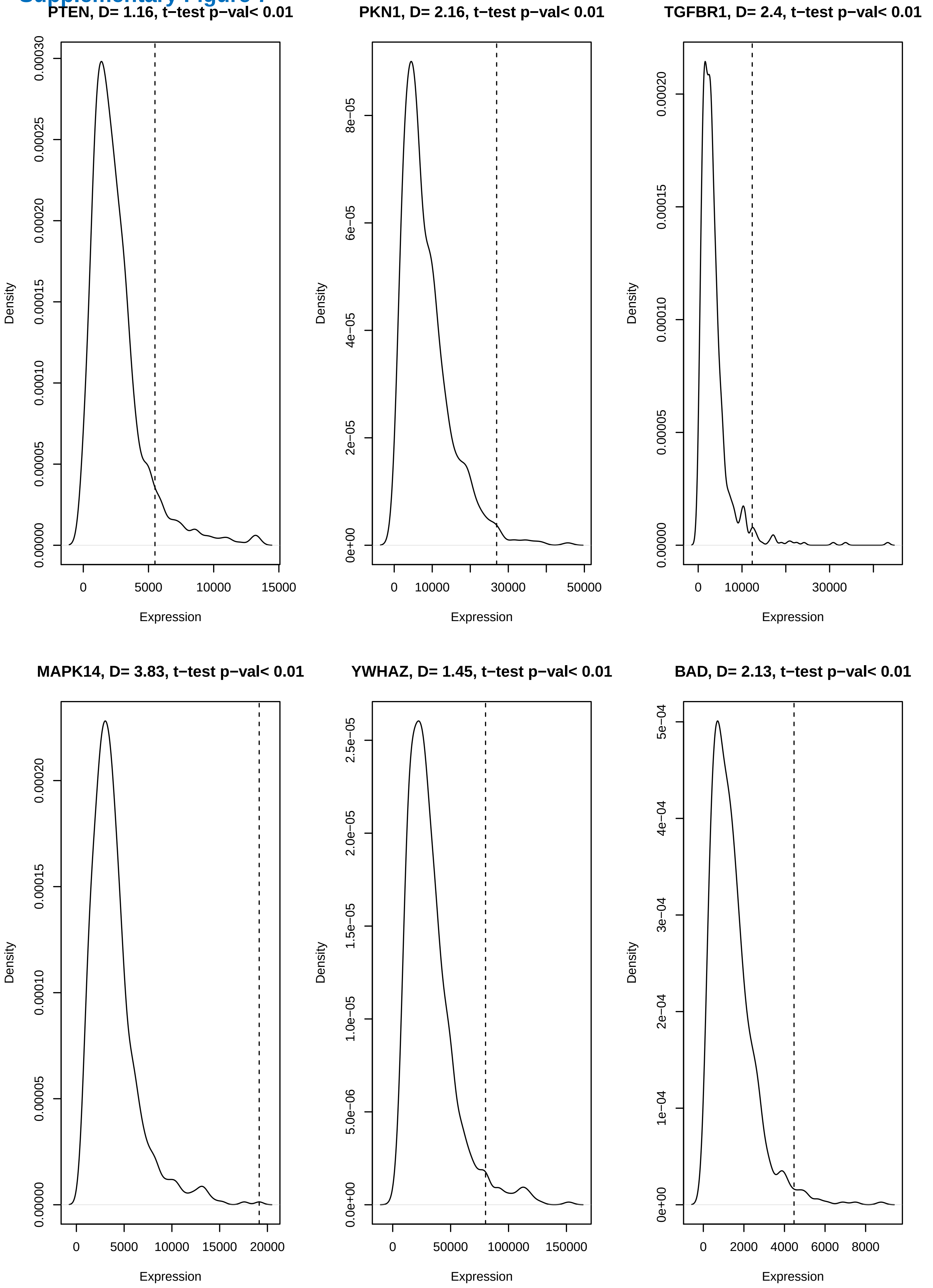

Supplementary Figure 8

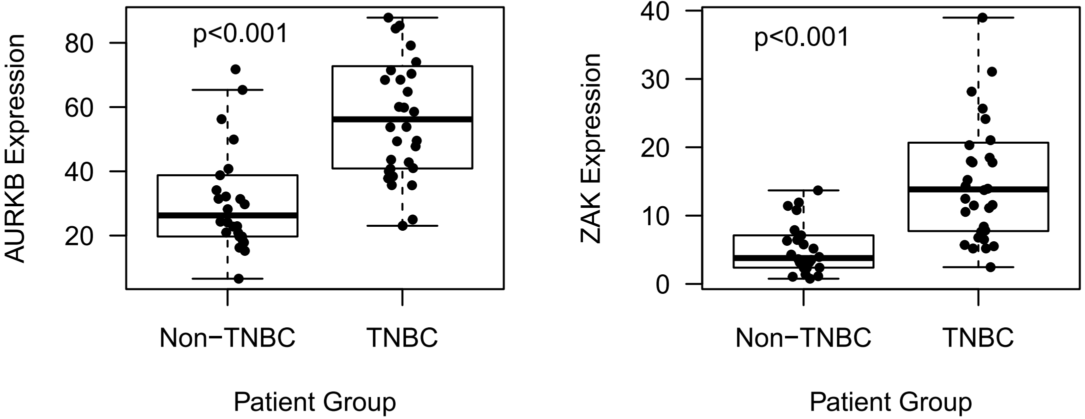

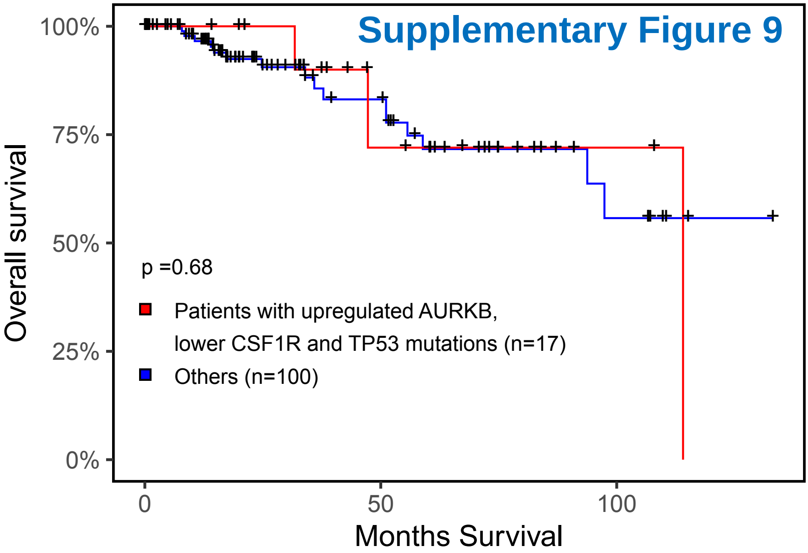
Supplementary Figure 9
Overall survival
100%
75%
50%
25%
0%
p =0.68
Patients with upregulated AURKB,
lower CSF1R and TP53 mutations (n=17)
Others (n=100)
0
50
100
Months Survival

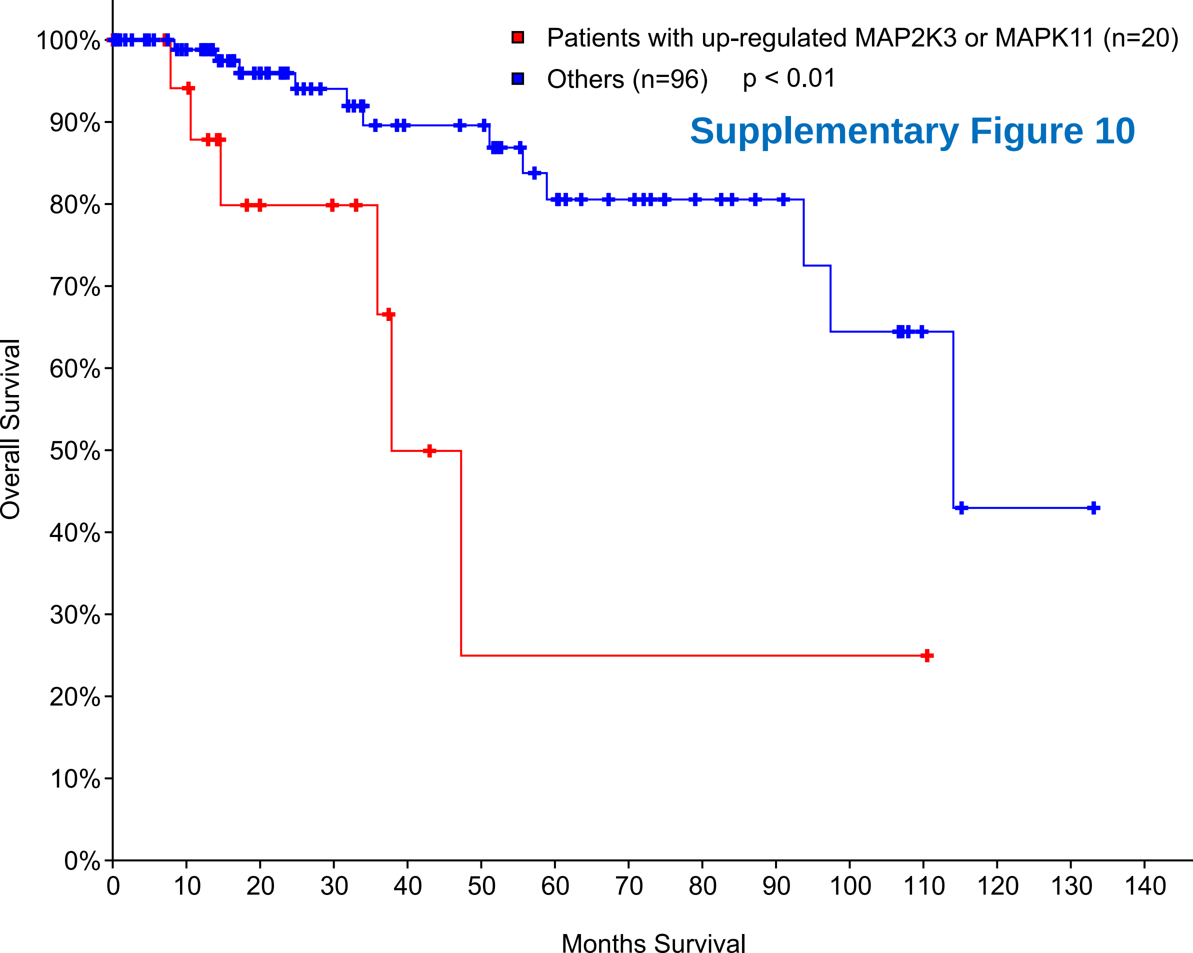
Patients with up-regulated MAP2K3 or MAPK11 (n=20)
Others (n=96)
p < 0.01
Supplementary Figure 10
Overall Survival
100%
90%
80%
70%
60%
50%
40%
30%
20%
10%
0%
0
10
20
30
40
50
60
70
80
90
100
110
120
130
140
Months Survival
