# Supplementary material for: Network pharmacology modeling identifies synergistic Aurora B and ZAK interaction in triple-negative breast cancer
Source: NPJ Syst Biol Appl. 2019 Jul 8;5:20. doi: 10.1038/s41540-019-0098-z (PMC6614366; doi:10.1038/s41540-019-0098-z)
Supplement: Supplementary file 18 — Supplementary Data 16 [file 41540_2019_98_MOESM18_ESM.zip › Supplementary Data 16 ReadMe.docx]

**Supplementary Data 16.** Folder 1: R and Matlab codes and data to repeat the TIMMA prediction results; Folder 2: model files for the stochastic simulation using the SGNS2 tool, and the ODE-based modeling using the COPASI tool.
